# STAMBPL1/TRIM21 Balances AXL Stability Impacting Mesenchymal Phenotype and Immune Response in KIRC

**DOI:** 10.1002/advs.202405083

**Published:** 2024-11-11

**Authors:** Shiyu Huang, Xuke Qin, Shujie Fu, Juncheng Hu, Zhengyu Jiang, Min Hu, Banghua Zhang, Jiachen Liu, Yujie Chen, Minghui Wang, Xiuheng Liu, Zhiyuan Chen, Lei Wang

**Affiliations:** ^1^ Department of Urology Renmin Hospital of Wuhan University Wuhan Hubei 430060 China; ^2^ Institute of Urologic Disease Renmin Hospital of Wuhan University Wuhan Hubei 430060 China; ^3^ Department of Cardiology Renmin Hospital of Wuhan University Wuhan Hubei 430060 China; ^4^ Hubei Key Laboratory of Digestive System Disease Wuhan 430060 China; ^5^ Central Laboratory Renmin Hospital of Wuhan University Wuhan Hubei 430060 China

**Keywords:** immunotherapy resistance, kidney renal clear cell carcinoma, mesenchymal phenotype, STAM binding protein like 1, ubiquitination

## Abstract

Kidney renal clear cell carcinoma (KIRC) is recognized as an immunogenic tumor, and immunotherapy is incorporated into its treatment landscape for decades. The acquisition of a tumor mesenchymal phenotype through epithelial‐to‐mesenchymal transition (EMT) is associated with immune evasion and can contribute to immunotherapy resistance. Here, the involvement of STAM Binding Protein Like 1 (STAMBPL1) is reported in the development of mesenchymal and immune evasion phenotypes in KIRC cells. Mechanistically, STAMBPL1 elevated protein abundance and surface accumulation of TAM Receptor AXL through diminishing the TRIM21‐mediated K63‐linked ubiquitination and subsequent lysosomal degradation of AXL, thereby enhancing the expression of mesenchymal genes while suppressing chemokines CXCL9/10 and HLA/B/C. In addition, STAMBPL1 enhanced PD‐L1 transcription via facilitating nuclear translocation of p65, and knockdown (KD) of STAMBPL1 augmented antitumor effects of PD‐1 blockade. Furthermore, STAMBPL1 silencing and the tyrosine kinase inhibitor (TKI) sunitinib also exhibited a synergistic effect on the suppression of KIRC. Collectively, targeting the STAMBPL1/TRIM21/AXL axis can decrease mesenchymal phenotype and potentiate anti‐tumor efficacy of cancer therapy.

## Graphical abstract

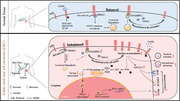

## 1 Introduction

Renal cell carcinoma (RCC) affects more than 400 000 individuals worldwide each year, with approximately 70% of cases being diagnosed as KIRC.^[^ ^1^ ^]^ Over the past decade, the treatment landscape of RCC has been advanced by targeted therapies and immune‐based therapies, more recently, combinations of immune checkpoint inhibitors (ICIs) with either other ICI or TKIs have demonstrated remarkable efficacy in patients with metastatic RCC (mRCC), which become the standard‐of‐care first‐line therapies for patients with this disease.^[^ ^2^ ^]^ Targeting immune evasion using ICIs has resulted in clinical responses in some RCC patients.^[^ ^3^ ^]^ Nevertheless, a number of patients eventually develop intrinsic or acquired resistance to ICIs, leading to treatment failure, hence it is urgently needed to identify the potential drivers of immune evasion in RCC and provide effective combination treatment strategies.^[^ ^2^, ^4^ ^]^

The acquisition of a mesenchymal phenotype via EMT is a crucial step for tumor metastasis. Increasing evidence suggests that immune evasion and immunotherapy resistance could occur as a consequence of EMT program.^[^ ^5^ ^]^ The immune‐suppressive tumor microenvironment (TME) can control both immune evasion and the response to ICIs, and the mesenchymal phenotype is known to be associated with TME features.^[^ ^6^ ^]^ Colorectal cancer (CRC) subtype with mesenchymal phenotype demonstrates aggressive behavior and unfavorable prognosis accompanied by an immunosuppressive TME.^[^ ^7^ ^]^ In non‐small cell lung cancer, EMT reprogrammed the immune response in the local tumor microenvironment, resulting in immune escape characterized by decreased CD3+ and CD8+ T‐cell infiltration and increased T‐cell exhaustion markers.^[^ ^8^ ^]^ Moreover, cancer cells that have acquired a mesenchymal phenotype could exhibit defective major histocompatibility complex class I (MHC‐I)‐mediated antigen presentation,^[^ ^5a^ ^]^ attenuated interferon (IFN) signaling^[^ ^9^ ^]^ and aberrant secretion of chemokines,^[^ ^10^ ^]^ all of which play a critical role in tumor immune response. Thus, targeting the mesenchymal phenotype to reshape the TME and modify the immune response of tumors would be a promising therapeutic approach for KIRC in clinical practice.

Ubiquitination is an essential type of post‐translational modification (PTM) and serves as a crucial regulator of the majority, if not all, signaling pathways, and dysfunctions in cellular signaling pathways significantly contribute to the progression of cancer.^[^ ^11^ ^]^ As isopeptidases involved in eukaryotic ubiquitylation, the deubiquitinating enzymes (DUBs) could remove the ubiquitin chain(s) from substrate proteins and regulate the degradation of selected proteins through the proteasome or lysosomal pathway, which have emerged as promising drug targets.^[^ ^12^ ^]^ Indeed, previous studies have suggested that the aberrant expression of DUBs was associated with mesenchymal phenotype and tumor progression.^[^ ^13^ ^]^ Wu et al. revealed that knockdown (KD) of deubiquitinating enzyme 3 suppressed EMT and tumor metastasis through destabilizing Snail1 in breast cancer.^[^ ^14^ ^]^ Li et al. reported ubiquitin‐specific peptidase 39 promoted EMT via inhibiting ZEB1 ubiquitination in hepatocellular carcinoma.^[^ ^15^ ^]^ DUBs and E3 ligases coordinate ubiquitin signaling and ubiquitination‐deubiquitination balance is of importance for protein quality control and biological activities.^[^ ^16^ ^]^ Additionally, disruption of this balance between E3 ubiquitin ligases and DUBs may implicate in the pathogenesis of a wide variety of diseases, including development of cancer.^[^ ^17^ ^]^ Nonetheless, the functional role for DUBs and ubiquitination‐deubiquitination balance in the mesenchymal phenotype regulation in KIRC remains incompletely understood.

Based on this background, STAMBPL1, a zinc dependent deubiquitinase, was identified as a regulator of the mesenchymal phenotype and immune response in KIRC. STAMBPL1 has been implicated in various cancer types by targeting different proteins.^[^ ^18^ ^]^ In prostate cancer, targeting the STAMBPL1 triggers apoptosis by promoting XIAP degradation.^[^ ^19^ ^]^ In breast cancer, STAMBPL1 contributes to cisplatin resistance by stabilizing MKP‐1 expression.^[^ ^20^ ^]^ Meanwhile, STAMBPL1 is also reported to be essential in the EMT process in lung and breast carcinomas.^[^ ^21^ ^]^ Here, it is demonstrated that STAMBL1/TRIM21 balances the level of AXL ubiquitination and thereby determines the expression of mesenchymal genes and immune evasion‐related genes. In addition, STAMBPL1 inhibition synergistically enhanced the antitumor effect of PD‐1 blockade as well as sunitinib, which provided a potential combination treatment strategy for patients with KIRC.

## 2 Results

### 2.1 STAMBPL1 Contributes to KIRC Cell Mesenchymal Phenotype and Immune Evasion

**Figure 1:**
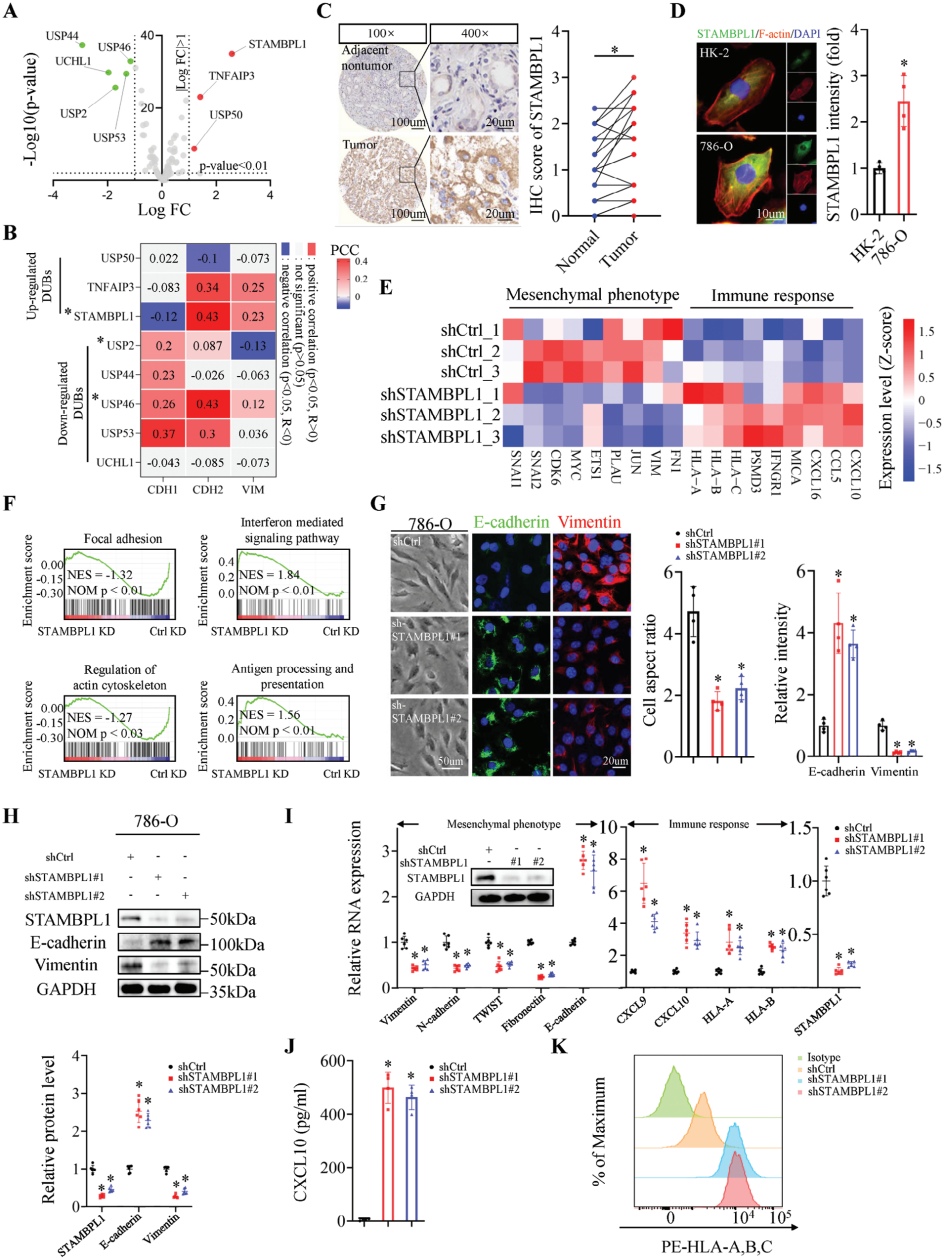
STAMBPL1 contributes to KIRC cell mesenchymal phenotype and immune evasion. A) Volcano plot of significantly upregulated (red) and downregulated (green) DUB genes in KIRC relative to the normal adjacent tissue. Differential analyses were performed using the R packages DESeq2 and cut off is | log2[fold change] | >1, P value < 0.01. B) Heatmap showing the correlation between the indicated DUB and CDH1, CDH2 or VIM, and correlation coefficients were calculated using the Pearson test. Two‐sided p‐value was given. C) Representative images and the quantitative results from IHC staining of STAMBPL1 in human KIRC tissues (n = 20) and matched adjacent normal tissues (n = 20). D) Representative IF images of STAMBPL1 (green) and F‐actin (red) in HK‐2 cells and 786‐O KIRC cells. DNA was stained with DAPI (blue). Relative intensities were assessed by Image J software (n = 4). E) Heatmap of mesenchymal and immune response genes from RNA‐seq for control and STAMBPL1 KD 786‐O cells (n = 3). F) GSEA enrichment plots for selected gene sets by RNA‐seq (STAMBPL1 KD versus control KD) were presented, displaying NES (Normalized Enrichment Score) and corresponding Nominal P‐values (NOM p). G) Representative brightfield images of control and STAMBPL1 KD 786‐O cells, and representative IF images of E‐cadherin (green) and Vimentin (red) (left panel). Cell aspect ratio and relative immunofluorescence intensities were quantified (right panel) (n = 4). H) Immunoblots and quantitative results of EMT‐related proteins (n = 6), GAPDH was used as loading control. I) RT‐qPCR analysis of mesenchymal and immune response genes in control and STAMBPL1 KD 786‐O cells (n = 6). J) Quantification of secreted CXCL10 from control and STAMBPL1 KD 786‐O cells was performed using ELISA (n = 4). K) Flow cytometry assays were used to analyze the effect of STAMBPL1 on HLA‐A/B/C surface expression in 786‐O cells (n = 4). All data are represented as mean ± SD, and analyzed using one‐way ANOVA followed by Tukey post hoc test. For the analysis in (C), a paired two‐tailed Student's t‐test was conducted. For the analysis in (D), an unpaired two‐tailed Student′s t test was performed. *p<0.05; PCC, Pearson's correlation coefficient; Ctrl, control.

Considering that a few of studies indicated the potential role of DUBs in regulating the mesenchymal phenotype and their promising prospects as drug targets for cancer therapy,^[^ ^21^, ^22^ ^]^ we focused our interest on DUBs. To systematically investigate the dysregulation of DUBs in KIRC patients, transcriptional levels of DUB genes were examined in The Cancer Genome Atlas (TCGA) cohort of human KIRC and our analysis revealed three DUB genes (USP50, TNFAIP3 and STAMBPL1) were significantly upregulated in tumors (fold change > 2, p < 0.01) (**Figure** **1**A). The mesenchymal phenotype and the EMT program are marked by E‐cadherin (encoded by CDH1) repression and N‐cadherin (encoded by CDH2) and Vimentin (encoded by VIM) induction.^[^ ^23^ ^]^ Following the correlation analysis between the upregulated DUB genes and key markers linked to the mesenchymal phenotype, it was found that among the three genes, only STAMBPL1 mRNA levels showed obvious inverse correlation with CDH1 in KIRC samples, while demonstrating a positive correlation with CDH2 and VIM (Figure 1B). Though TNFAIP3 lacked correlation with CDH1 (Figure S1A, Supporting Information), it demonstrated a substantial correlation with CDH2 and VIM (Figure 1B). Nevertheless, subsequent functional experiments revealed that knockdown of TNFAIP3 did not impact the mesenchymal phenotype of KIRC cells (Figure S1B–D, Supporting Information). Thus, STAMBPL1 was selected for further study.

To confirm STAMBPL1 overexpression in KIRC, a series of biological experiments were performed and the public transcriptomic datasets were interrogated. Consistent with the above‐mentioned results, STAMBPL1 was significantly upregulated in KIRC cell lines and KIRC tissues compared to normal kidney tubular epithelial cell HK‐2 and adjacent non‐tumor tissues at mRNA and protein level (Figure 1C,D; Figure S2A–D, Supporting Information). Then 786‐O cells infected with lentiviruses carrying shCtrl or shSTAMBPL1#1 were harvested for RNA sequencing (RNA‐seq). Knockdown efficiency of short hairpin RNAs (shRNAs) for STAMBPL1 in KIRC cells was shown in Figure S3A,B (Supporting Information). RNA‐seq analysis revealed that STAMBPL1 KD led to downregulation of EMT‐associated genes (e.g., SNAI1, SNAI2, CDK6, MYC, ETS1, PLAU, JUN, VIM and FN1) and upregulation of immune stimulatory genes (e.g., HLA‐A, HLA‐B, HLA‐C, PSMD3, IFNGR1, MICA, CXCL16, CCL5 and CXCL10) (Figure 1E). Additionally, Gene set enrichment analysis (GSEA) of STAMBPL1 KD RNA‐seq showed downregulation of pathways related to cell movement, such as focal adhesion and regulation of actin cytoskeleton, while immune stimulatory pathways, including interferon mediated signaling pathways and antigen processing and presentation, were upregulated (Figure 1F). Interrogation of TCGA database also revealed that STAMBPL1 expression correlated with antitumor immune response pathways (Figure S3C, Supporting Information). The functional consequences of silencing STAMBPL1 were evaluated in two KIRC cell lines (786‐O and 769‐P) using two independent shRNAs. Immunofluorescence (IF) and immunoblotting assays demonstrated that STAMBPL1 KD in KIRC cells led to reduced mesenchymal markers and increased epithelial markers (Figure 1G,H; Figure S5A,C, Supporting Information). The morphological observations exhibited a flattened, cuboidal cellular phenotype, characteristic of cells undergoing a mesenchymal‐to‐epithelial transition (MET) in response to STAMBPL1 KD (Figure 1G; Figure S5B, Supporting Information). Furthermore, quantitative RT‐PCR (RT‐qPCR) analysis verified that STAMBPL1 KD led to the downregulation of multiple mesenchymal markers, including Vimentin, N‐cadherin, TWIST, and Fibronectin and upregulation of E‐cadherin, chemokines CXCL9 and CXCL10, and HLA‐A and HLA‐B (Figure 1I; Figure S5D, Supporting Information). Increased CXCL10 secretion and enhanced surface expression of HLA‐A/B/C in STAMBPL1 KD KIRC cells were confirmed by ELISA and flow cytometry, respectively (Figure 1J,K; Figure S5E,F, Supporting Information). Conversely, STAMBPL1 overexpression in 786‐O cells resulted in enhancements in mesenchymal and immune evasion phenotypes (Figure S4A–D, Supporting Information). Taken together, these data suggest STAMBPL1 as a modulator of mesenchymal maintenance and immune response in KIRC cells.

### 2.2 STAMBPL1 Inhibition Enhances AXL Degradation via the Ubiquitin‐Lysosome Pathway

**Figure 2:**
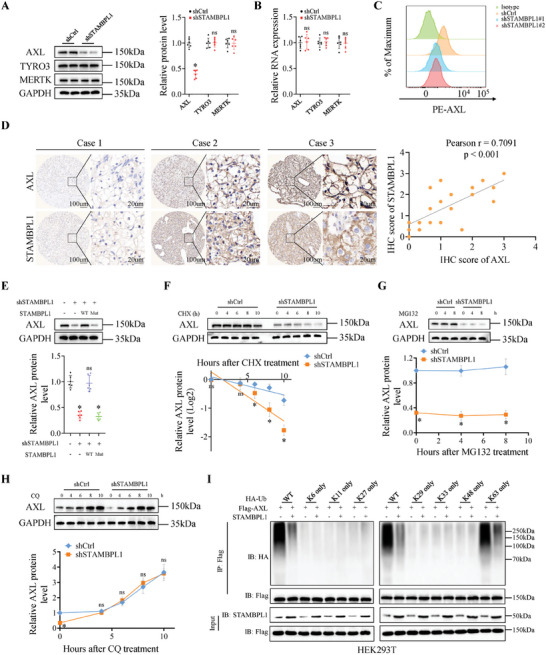
STAMBPL1 inhibition enhances AXL degradation via the ubiquitin‐lysosome pathway. A) Immunoblots and quantitative results of TAM receptors proteins in control and STAMBPL1 KD 786‐O cells (n = 6), GAPDH was used as loading control. B) RT‐qPCR analysis of TAM receptors genes in control and STAMBPL1 KD 786‐O cells (n = 6). C) Flow cytometry analysis of AXL surface expression on control and STAMBPL1 KD 786‐O cells (n = 4). D) Tumor tissues of KIRC samples were subjected to IHC staining for AXL and STAMBPL1 (left panel) (n = 20). Scatter plot illustrating the correlation of the IHC scores corresponding to AXL and STAMBPL1 (right panel). Pearson's correlation coefficient r with P‐value are shown. E) Immunoblotting analysis of whole‐cell lysates (WCL) derived from control and STAMBPL1 KD 786‐O cells transfected with indicated constructs (top panel). Quantitative results (bottom panel) (n = 6). F) Immunoblotting analysis of WCL derived from control and STAMBPL1 KD 786‐O cells treated with 100 µg mL^−1^ CHX at indicated time points (top panel). AXL band intensity was normalized to GAPDH and then to the t = 0 time point (bottom panel) (n = 6). G,H) Control and STAMBPL1 KD 786‐O cells were treated with 10 µM MG132 or 50 µM CQ for the indicated hours, the WCL were immunoblotted for AXL or GAPDH (top panel). AXL band intensity was normalized to GAPDH and then to the t = 0 time point (bottom panel) (n = 6). I) HEK293T cells were transfected with the indicated plasmids, and the ubiquitination of AXL was detected by Co‐IP and immunoblotting (n = 3). All data are represented as mean ± SD, and analyzed using an unpaired two‐tailed Student′s t test. For the analysis in (D), Pearson correlation test was conducted. For the analysis in (E), one‐way ANOVA followed by Tukey post hoc test was performed. *p<0.05; ns, not significant; Ctrl, control; WT, wild type; Mut, mutant; CHX, cycloheximide; CQ, chloroquine.

Based on previous reports,^[^ ^24^ ^]^ the significance of TAM (TYRO3/AXL/MERTK) receptors in mesenchymal and immune evasion phenotypes prompted us to examine their potential as downstream mediators of STAMBPL1 in KIRC. TAM family of receptor tyrosine kinases have critical roles in a range of diseases including cancer, myocardial infarction and viral infections.^[^ ^25^ ^]^ Specifically, the aberrant activity of TAM receptors could accelerate tumor development by modulating various oncogenic pathways, encompassing cancer cell mesenchymal phenotype maintenance,^[^ ^26^ ^]^ immune microenvironment regulation,^[^ ^27^ ^]^ and resistance to immunotherapy.^[^ ^28^ ^]^ To validate our hypothesis, we analyzed TAM receptors protein levels in control and STAMBPL1 KD cells and discovered that protein abundance of AXL, but not other TAM receptors, dramatically decreased in STAMBPL1 KD cells (**Figure** **2**A). However, we did not observe any change in AXL mRNA level (Figure 2B). Cell surface AXL on KIRC cells was also significantly decreased with STAMBPL1 KD (Figure 2C), whereas STAMBPL1 KD had no effect on the cell surface levels of TYRO3 and MERTK (Figure S6A,B, Supporting Information). Of note, immunohistochemical (IHC) staining results exhibited a positive correlation between STAMBPL1 and AXL in human KIRC specimens (n = 20; Pearson's correlation coefficient r = 0.7091, P < 0.001), further supporting the notion that STAMBPL1 could positively regulate AXL protein abundance (Figure 2D). Due to the significant decrease in total and membrane AXL protein abundance upon STAMBPL1 downregulation, while AXL mRNA levels remained largely unchanged (Figure 2A–C), we reasoned that STAMBPL1 might regulate the degradation of AXL protein or its mRNA cellular distribution and translation. Notably, using RNA fluorescence in situ hybridization (RNA FISH) and cell fractionation analysis, we found that STAMBPL1 could not affect the subcellular location of AXL mRNAs (Figure S6C–F, Supporting Information). In addition, we assessed ribosome density on AXL mRNA fractions through sucrose gradient centrifugation in 786‐O cells. The RT‐qPCR results indicated that STAMBPL1 KD did not impact the polysome density (> 80S) on the AXL mRNA (Figure S6G, Supporting Information). These data suggested that the reduction in AXL protein levels mediated by STAMBPL KD was not attributed to alterations in AXL mRNA cellular distribution and translation efficiency.

Given that STAMBPL1 plays an important role in protein stability regulation and cell signaling through its deubiquitinating activity.^[^ ^29^ ^]^ To examine whether the DUB activity of STAMBPL1 was crucial for the modulation of AXL, we mutated the water‐activating Glu 292 (E292A) and the Zn^2+^‐coordinating Asp 360 (D360A) residues in STAMBPL1, which could completely block the DUB activity of STAMBPL1 while preserving its binding capability^[^ ^30^ ^]^ (Figure S7A,B, Supporting Information). Re‐expression of WT STAMBPL1, but not the catalytically inactive STAMBPL1 mutant (E292A & D360A), led to the recovery of AXL protein levels in STAMBPL1 KD KIRC cells (Figure 2E). Previous studies have shown that the DUB activity of STAMBPL1 frequently results in the removal of ubiquitin chains from target protein substrates and changes in protein stability.^[^ ^20^ ^]^ Considering the fact that STAMBPL1 regulated AXL in a DUB activity‐dependent manner, it is tempting to speculate that STAMBPL1 might stabilize AXL protein via attenuating ubiquitination of AXL. Next, the half‐life of AXL protein in STAMBPL1 KD cells was examined by a cycloheximide (CHX) chase assay and the results showed that STAMBPL1 KD reduced the protein stability of AXL compared with the control (Figure 2F). Proteins are mainly degraded by the proteasomal or lysosomal pathway.^[^ ^31^ ^]^ To explore which pathway plays a major role in regulating the AXL protein stability, we treated KIRC cells with either MG132 (proteasome inhibitor) or chloroquine (CQ; lysosome inhibitor) and monitored AXL protein level over time. As shown in Figure 2G,H, AXL level was much lower in STAMBPL1 KD KIRC cells than that in control cells, CQ treatment elevated AXL protein to the similar level as in control cells, in contrast, MG132 treatment failed to affect AXL protein level, indicating STAMBPL1 mainly control the AXL protein stability through lysosome system. Subsequently, we examined whether STAMBPL1 could directly regulate the ubiquitination level of AXL and the result of ubiquitination assay showed that STAMBPL1 overexpression in HEK293T cells attenuated the ubiquitination of AXL, whereas deletion of the DUB activity of STAMBPL1 abrogated this effect (Figure S8A, Supporting Information). Moreover, inhibition of STAMBPL1 using shRNA also increased the endogenous ubiquitination on AXL in KIRC cells (Figure S8B, Supporting Information). In order to enhance the reliability of our conclusion regarding the STAMBPL1 KD‐mediated destabilization of AXL, primary cancer cells were isolated from KIRC tissues. It was observed that STAMBPL1 KD markedly reduced the abundance of AXL without affecting AXL RNA levels in primary cancer cells (Figure S7C,D, Supporting Information). Compared to the control cells, the protein half‐life of endogenous AXL was dramatically decreased and the ubiquitination of AXL was significantly increased in shSTAMBPL1 treated primary cancer cells (Figure S7E,F, Supporting Information).

Generally, ubiquitinated proteins undergo proteasomal‐dependent degradation pathway. However, emerging evidence indicates that ubiquitin‐dependent lysosomal pathway serves as an essential cellular mechanism for protein degradation as well.^[^ ^32^ ^]^ To identify the specific type of polyubiquitination through which STAMBPL1 impacts AXL degradation, we employed a panel of ubiquitin mutants (K6, K11, K27, K29, K33, K48, and K63) with a single preserved lysine residue while substituting the remaining lysine residues with arginine. The results showed that STAMBPL1 specifically inhibited the ubiquitination of AXL in the presence of HA‐tagged K63‐specific ubiquitin, but not other linkage‐specific ubiquitin (Figure 2I). Moreover, the in vivo ubiquitination assay demonstrated that STAMBPL1 could not suppress AXL ubiquitination in the presence of the K63R mutant ubiquitin, which harbored a lysine‐to‐arginine substitution at position 63 (Figure S8C, Supporting Information). These findings suggested that STAMBPL1 could directly remove the K63‐linked ubiquitin chain on AXL.

In eukaryotic cells, protein degradation via the proteasome and lysosome is a dynamic process regulated by ubiquitin.^[^ ^33^ ^]^ Distinct types of ubiquitin linkages can lead to different degradation pathways, with K63 chains closely associated with the lysosomal pathway.^[^ ^34^ ^]^ The endosomal sorting complex required for transport (ESCRT) machinery, consisting of HRS, STAM1 and STAM2, plays a crucial role in ubiquitin‐dependent lysosomal degradation.^[^ ^35^ ^]^ To confirm that STAMBPL1 stabilized AXL by inhibiting the ubiquitin‐lysosome pathway, we examined whether the ESCRT complexes were involved in the degradation of AXL. Using CHX to block the protein synthesis of AXL, we found that the protein half‐life of endogenous AXL was dramatically prolonged in shHRS, shSTAM1 or shSTAM2 cells (Figure S9A–D, Supporting Information). Consistently, our findings showed that the impact of STAMBPL1 KD on AXL protein level was significantly counteracted by shHRS, shSTAM1 or shSTAM2 (Figure S9E, Supporting Information). Next, we wanted to determine whether ubiquitinated AXL was detected by the ESCRT complexes. Endogenous and exogenous Co‐IP studies confirmed the interaction of AXL with core ESCRT components like HRS, STAM1, and STAM2 (Figure S10A,B, Supporting Information). Importantly, overexpressing K63‐ubiquitin in KIRC cells could increase the binding of ESCRT complexes to AXL (Figure S10C,D, Supporting Information), and this interaction between ESCRT complexes and the substrate is crucial for subsequent lysosomal degradation.^[^ ^34^, ^35^ ^]^ As expected, we also observed that STAMBPL1 KD enhanced K63‐linked ubiquitination of AXL, strengthening the interaction between AXL and the ESCRT complexes (Figure S10E, Supporting Information). Conversely, overexpression of STAMBPL1 inhibited K63‐linked ubiquitination of AXL, suppressing the ESCRT complexes recognition of AXL (Figure S10F, Supporting Information). In summary, these findings demonstrated that STAMBPL1 increased AXL protein stability by impeding K63‐linked ubiquitin‐mediated recruitment of the ESCRT complexes and subsequent lysosomal degradation.

### 2.3 STAMBPL1 Specifically Interacts with AXL and STAMBPL1 KD Effects Are Largely Rescued by AXL Expression

Consistent with previous reports,^[^ ^36^ ^]^ we observed targeting AXL phenocopied STAMBPL1 KD in suppressing mesenchymal phenotypes and promoting immune stimulatory genes in KIRC cells (Figure S11A–F, Supporting Information). Furthermore, STAMBPL1 could affect the stability of AXL through deubiquitination according to our data (Figure 2F–I). Therefore, we hypothesized that AXL was necessary for STAMBPL1‐induced enhancement of mesenchymal and immune evasion phenotypes of KIRC cells. Before addressing this issue, further investigation was needed to uncover the specific mechanism by which STAMBPL1 modulated AXL ubiquitination.

**Figure 3:**
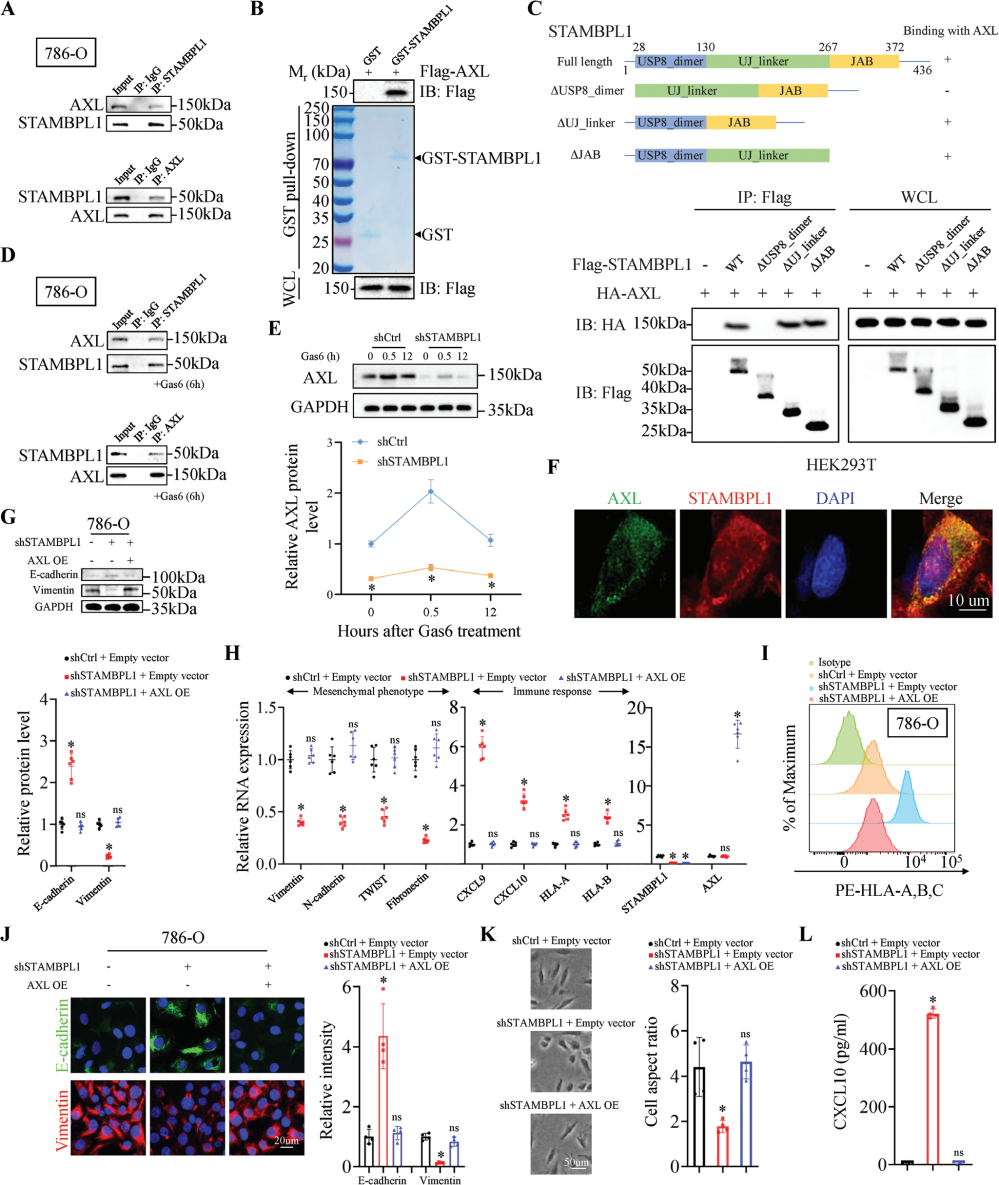
STAMBPL1 specifically interacts with AXL and STAMBPL1 KD effects are largely rescued by AXL expression. A) Co‐IP analysis of the endogenous STAMBPL1/AXL proteins interaction in the 786‐O cells (n = 3). IgG, IP control. B) Immunoblotting analysis of GST pull‐down precipitates from 786‐O cell lysates with ectopic expression of Flag‐AXL incubated with bacterially purified recombinant GST or GST‐STAMBPL1 protein (n = 3). C) Schematic representation of full length STAMBPL1 and its deletion mutants (top panel). HEK293T cells were co‐transfected with HA‐AXL and Flag‐tagged full length STAMBPL1 or its deletion mutants, and cell lysates were analyzed by IP with Flag antibody and protein A/G beads followed by immunoblotting analysis with antibodies against HA and Flag (bottom panel) (n = 3). D) 786‐O cells were stimulated with Gas6 (500 ng mL^−1^) for 6 h and immunoprecipitated with the indicated antibodies or normal IgG, and the precipitates were analyzed by immunoblotting (n = 3). E) Immunoblotting analysis of WCL derived from control and STAMBPL1 KD 786‐O cells treated with human recombinant Gas6 (500 ng mL^−1^) for the indicated times (top panel). AXL band intensity was normalized to GAPDH and then to the t = 0 time point (bottom panel) (n = 3). F) Cellular colocalization of AXL and STAMBPL1 proteins in 786‐O cells was analyzed by IF staining (n = 3). G) Immunoblots and quantitative results of EMT‐related proteins (n = 6), GAPDH was used as loading control. H) RT‐qPCR analysis of mesenchymal and immune response genes in 786‐O cells infected with the indicated lentiviral particles (n = 6). I) Cell surface HLA‐A/B/C expression was analyzed by flow cytometry (n = 4). J) Representative IF images and the quantitative results of EMT‐related proteins, including E‐cadherin and Vimentin, in indicated groups (n = 4). K) Representative brightfield images of 786‐O cells infected with the indicated lentiviruses (left panel). Cell aspect ratio was quantified (right panel) (n = 4). L) ELISA quantification of CXCL10 secreted from control and STAMBPL1 KD 786‐O cells transfected with indicated constructs (n = 4). All data are represented as mean ± SD, and analyzed using one‐way ANOVA followed by Tukey post hoc test. For the analysis in (E), an unpaired two‐tailed Student′s t test was performed. *p<0.05; ns, not significant; Ctrl, control; WT, wild type; WCL, whole‐cell lysates; OE, overexpressing.

DUBs employ diverse mechanisms and modes of interaction with their substrates to mediate the removal of mono‐ubiquitin or poly‐ubiquitin chains from the target protein.^[^ ^37^ ^]^ To ascertain the direct interaction between STAMBPL1 and AXL, Immunoprecipitation (IP) and in vitro pull‐down assays were performed. Indeed, endogenous binding between STAMBPL1 and AXL was observed in multiple KIRC cell lines (786‐O, 769‐P and Caki‐1), and the glutathione S‐transferase (GST) pull‐down experiments showed that AXL interacted with purified GST‐STAMBPL1, but not GST alone (**Figure** **3**A,B; Figure S12A, Supporting Information). Based on their interaction, we next identified the critical region within STAMBPL1 that binds to AXL. We generated three STAMBPL1 truncations lacking different domains: STAMBPL1‐ΔUSP8_dimer (deleting amino acid residues from 1 to 130), STAMBPL1‐ΔUJ_linker (deleting amino acid residues from 131 to 267) and STAMBPL1‐ΔJAB (deleting amino acid residues from 268 to 436) mutants. Then the STAMBPL1 deletion mutants, along with full‐length STAMBPL1 were transiently co‐transfected with HA‐tagged AXL plasmids into HEK293T cells. Our results showed truncated mutant lacking USP8_dimer domain exhibited much lower binding affinity with AXL comparing with STAMBPL1‐WT, STAMBPL1‐ΔUJ_linker and STAMBPL1‐ΔJAB, indicating that STAMBPL1 interacts with AXL largely through USP8_dimer domain (Figure 3C). AXL protein contains an extracellular (EC) domain, a transmembrane (TM) domain, and an intracellular (IC) domain,^[^ ^38^ ^]^ according to its structure, we constructed two AXL deletion mutants as indicated, as shown in Figure S12B (Supporting Information), mutants of AXL (amino acid 1–473 containing EC and TM domain) was unable to interact with STAMBPL1‐USP8_dimer, suggesting that the IC domain of AXL is the binding site for STAMBPL1. Extracellular growth arrest‐specific factor 6 (GAS6) is a ligand that facilitates AXL activation by receptor dimerization, thereby initiating signaling cascades including PI3K‐AKT, MAPK, STAT, and NF‐κB pathways.^[^ ^39^ ^]^ Thus, the potential effect of GAS6 on STAMBPL1 KD‐induced AXL protein downregulation, and whether STAMBPL1 and GAS6 were two independent regulators of AXL warrant further investigation. First, our data showed that the interaction between STAMBPL1 and AXL proteins in KIRC cells was not affected upon GAS6 stimulation (Figure 3D). Second, GAS6 treatment could cause transient activation of AXL in control KIRC cells or STAMBPL1 KD cells, nonetheless, AXL protein levels remained significantly higher in control cells than in STAMBPL1 KD cells following GAS6 stimulation (Figure 3E). Importantly, the GAS6‐induced phosphorylation of AXL was also unaffected by STAMBPL1, as evidenced in both STAMBPL1 KD KIRC cells and STAMBPL1‐overexpressing KIRC cells, despite this activation being transient (Figure S12C,D, Supporting Information). In addition, IF staining demonstrated a significant co‐localization of endogenous STAMBPL1 and AXL in KIRC cells (Figure 3F). Collectively, these findings suggest that STAMBPL1 directly binds to AXL IC domain, and this interaction remains unaffected by GAS6.

We then investigated the requirement for AXL in the STAMBPL1‐mediated regulation of mesenchymal and immune evasion phenotypes, STAMBPL1 KD KIRC cells were infected with the lentivirus to overexpress AXL and the efficiency was confirmed by immunoblotting assays (Figure S12E, Supporting Information). As expected, ectopic expression of AXL remarkably rescued the decreased mesenchymal phenotype and increased immune stimulatory genes expression in STAMBPL1 KD cells (Figure 3G,H,J,K), meanwhile, the upregulated HLA‐A/B/C surface expression and CXCL10 secretion in STAMBPL1‐silenced cells were largely rescued by AXL expression as well (Figure 3I,L), which was consistent with the previous hypothesis. Taken together, these data provided sufficient experimental evidence that genetic overexpression of AXL largely abolished the effects caused by STAMBPL1 KD in KIRC cells.

The suppressor of cytokine signaling 1 (SOCS1) and suppressor of cytokine signaling 3 (SOCS3), downstream effectors of AXL signaling, play crucial roles in modulating tumor immune responses and immunotherapy sensitivity.^[^ ^40^ ^]^ AXL KD decreased SOCS1 and SOCS3 in KIRC cells (Figure S13A,B, Supporting Information). Additionally, STAMBPL1 could also regulate the expression levels of SOCS1/3 through AXL (Figure S13C–F, Supporting Information). Next, we investigated the importance of SOCS1/3 in STAMBPL1‐mediated immune evasion. As depicted in Figure S14A–G (Supporting Information), expression of proinflammatory cytokine suppressor and AXL signaling effector SOCS1/3 rescued the increased CXCL9/10 and HLA‐A/B induced by STAMBPL1 KD. Collectively, these data indicated that STAMBPL1 promoted immune evasion phenotypes in KIRC cells, at least in part, by stimulating AXL mediated upregulation of SOCS1/3.

### 2.4 STAMBPL1 and E3 Ligase TRIM21 Balance the Level of AXL K63‐Linked Ubiquitination

**Figure 4:**
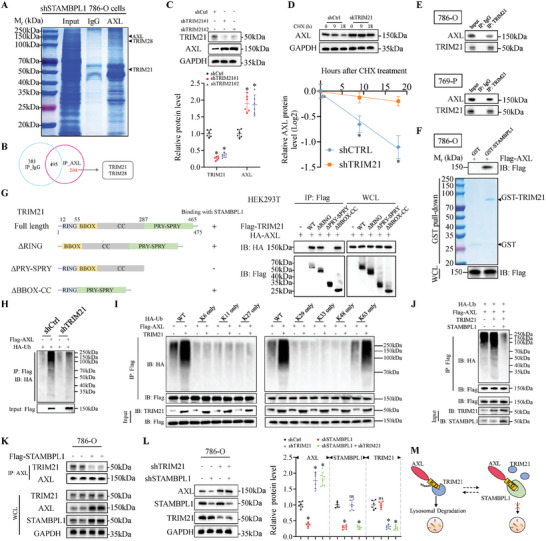
STAMBPL1 and E3 ligase TRIM21 balance the level of AXL K63‐linked ubiquitination. A,B) Coomassie blue staining and mass spectrometry analysis of AXL immunoprecipitation complex in STAMBPL1 KD 786‐O cells. C) Immunoblotting analysis of WCL derived from control and TRIM21 KD 786‐O cells (n = 6). D) Immunoblotting analysis of WCL derived from control and TRIM21 KD 786‐O cells treated with 100 µg mL^−1^ CHX at indicated time points (top panel). AXL band intensity was normalized to GAPDH and then to the t = 0 time point (bottom panel) (n = 6). E) Co‐IP analysis of the endogenous TRIM21/AXL proteins interaction in the 786‐O cells and 769‐P cells. IgG, IP control (n = 3). F) Immunoblotting analysis of AXL proteins in 786‐O cells lysates pulled down by GST or GST‐TRIM21 recombinant proteins (n = 3). G) A schematic illustration of full length TRIM21 and its deletion mutants (left panel). Immunoblotting analysis of WCL and anti‐Flag immunoprecipitates (IPs) from HEK293T cells co‐transfected with indicated constructs (right panel) (n = 3). H) HEK293T cells were transfected with the indicated shRNAs and constructs. Cell lysates were subjected to IP with Flag antibody, followed by immunoblotting analysis with antibodies against HA and Flag (n = 3). I) In vivo ubiquitination assay using HEK293T cells transfected with the indicated plasmids to detect ubiquitination of Flag‐AXL (n = 3). J) Immunoblotting analysis of WCL and anti‐Flag IPs derived from lysates of HEK293T cells co‐transfected with indicated constructs (n = 3). K) Co‐IP of TRIM21 with endogenous AXL in 786‐O cells transfected with the indicated empty vectors or Flag‐tagged STAMBPL1 vectors (n = 3). L) Immunoblots and quantitative results of AXL in 786‐O cells infected with lentiviruses carrying indicated shRNAs (n = 6). M) Model of STAMBPL1 inhibition of TRIM21‐mediated lysosomal degradation of AXL. All data are represented as mean ± SD, and analyzed using one‐way ANOVA followed by Tukey post hoc test. For the analysis in (D), an unpaired two‐tailed Student′s t test was performed. *p<0.05; ns, not significant; Ctrl, control; WT, wild type; WCL, whole‐cell lysates.

Ubiquitination, a reversible PTM, is coordinated by E3 ubiquitin ligases and DUBs,^[^ ^41^ ^]^ E3 ligases mediated the transfer of ubiquitin from an E2 ubiquitin‐conjugating enzyme to specific substrate proteins, in contrast, the attachment of ubiquitin signals could be directly antagonized by the action of DUBs.^[^ ^42^ ^]^ We have demonstrated that STAMBPL1 inhibits the ubiquitination of AXL in KIRC cells, nevertheless, the specific E3 ligase(s) responsible for destabilizing AXL and promoting its ubiquitination remains unidentified. E3 ubiquitin ligases typically facilitate ubiquitination by interacting with substrates, and the high specificity of ubiquitination relies on this interaction.^[^ ^43^ ^]^ STAMBPL1 KD could enhance AXL ubiquitination and strengthen the interaction between E3 ligases and AXL. Hence, to further investigate the molecular basis of AXL ubiquitination, co‐immunoprecipitation (Co‐IP) combined with mass spectrometry (MS) analysis was performed in STAMBPL1 KD 786‐O cells to determine AXL‐interacting E3 ligase candidates. Romain et al. reported that E3‐ubiquitin ligase CBL played a role in the ubiquitination of AXL,^[^ ^39^ ^]^ but the result of MS analysis did not detect the interaction between CBL and AXL, while two E3 ligases, TRIM21 and TRIM28, were identified (**Figure** **4**A,B; Figure S15A, Supporting Information). Then we examined whether this two E3 ligases could affect the level and half‐life of AXL protein. As shown in Figure 4C,D and Figure S15B,C (Supporting Information), AXL protein expression and stability were significantly increased by TRIM21 KD, whereas TRIM28 KD had no impact on AXL protein, indicating the E3 ligase TRIM21 might destabilize AXL in KIRC cells. In keeping with this notion, we demonstrated a negative correlation between TRIM21 and AXL levels in human KIRC specimens (n = 20) by IHC (Pearson's correlation coefficient r = −0.6613, P < 0.01; Figure S15E, Supporting Information). Additionally, a significant increase in membrane AXL protein abundance were also observed following TRIM21 KD, however, the AXL mRNA level remained unchanged (Figure S15D,F, Supporting Information). The results of Co‐IP and GST‐pull down assays in KIRC cell lines confirmed the interactions between TRIM21 and AXL (Figure 4E,F; Figure S16A, Supporting Information). Considering their interaction, we subsequently identified the binding regions between TRIM21 and AXL. We generated three deletion mutants of TRIM21 (TRIM21‐ΔRING, TRIM21‐ΔPRY‐SPRY and TRIM21‐ΔBBOX‐CC) and two deletion mutants of AXL (AXL‐ΔIC and AXL‐ΔEC+TM), the data showed that the PRY‐SPRY domain of TRIM21 (amino acid residues from 287 to 465) and IC domain of AXL (amino acid residues from 473 to 894) were necessary for their interaction (Figure 4G; Figure S16B, Supporting Information). Next, we found that the polyubiquitination level of AXL was markedly attenuated in TRIM21 KD cells (Figure 4H; Figure S16C, Supporting Information). These findings collectively indicate that the E3 ligase TRIM21 negatively regulates AXL at the post‐translational level mainly through promoting the ubiquitinated degradation of AXL.

Previous reports demonstrated that TRIM21 could catalyze K63‐ubiquitin chain formation, stimulating diverse pathways.^[^ ^44^ ^]^ Here, in vivo ubiquitination assay showed that ectopic expression of TRIM21 dramatically promoted the K63‐linked ubiquitination of AXL in cells (Figure 4I). We then designed His‐tagged ubiquitin mutants at 6/11/27/29/33/48/63 lysine of arginine (K6R, K11R, K27R, K29R, K33R, K48R, K63R) to specifically prevent the formation of K6‐, K11‐, K27‐, K29‐, K33‐, K48‐, or K63‐linked chain, and observed that lys63‐ubiquitin mutant (K63R), but not other ubiquitin mutants, significantly inhibited the formation of AXL polyubiquitylation chain, supporting that TRIM21 promotes AXL polyubiquitylation via the K63 linkage (Figure S16D, Supporting Information). Next, we need to determine whether TRIM21‐mediated K63‐linked polyubiquitylation would trigger lysosomal‐dependent degradation of AXL. As shown in Figure S17A,B (Supporting Information), TRIM21 overexpression markedly decreased the protein level of AXL, and this effect could be recovered by adding the lysosomal inhibitor CQ but not the proteasomal inhibitor MG132. In addition, TRIM21 increased K63‐linked ubiquitination of AXL and enhanced its interaction with core components of the ESCRT complexes (HRS, STAM1, and STAM2), whereas TRIM21 KD reduced ubiquitination of AXL, thereby weakening this interaction. These results suggested the involvement of the ESCRT complexes in TRIM21‐mediated AXL degradation (Figure S17C,D, Supporting Information). Consistent with a proposed role for TRIM21 in promoting AXL ubiquitination and degradation, TRIM21 KD KIRC cells exhibited increased mesenchymal and decreased immune response phenotypes compared with control cells (Figure S18A–D, Supporting Information).

Since STAMBPL1 could remove the ubiquitin chain on AXL, we subsequently investigate whether STAMBPL1 can diminish the TRIM21‐mediated K63‐linked ubiquitination on AXL. It was worth noting that the introduction of exogenous STAMBPL1 significantly reduced the TRIM21‐facilitated ubiquitination of AXL in cells (Figure 4J). E3 ligases and DUBs could balance substrate ubiquitination levels through competitive or non‐competitive binding mechanisms,^[^ ^45^ ^]^ for example, E3 ligase TRIM32 and the DUB USP7 could form a complex with c‐Myc to balance the level of c‐Myc ubiquitination.^[^ ^46^ ^]^ To gain insight into how STAMBPL1/TRIM21 balance the ubiquitination of AXL, the interaction of the three proteins (STAMBPL1, TRIM21 and AXL) was further explored. The aforementioned results have revealed the IC region of AXL interacting with STAMBPL1 USP8_dimer domain and TRIM21 PRY‐SPRY domain respectively (Figure S12B; Figure S16B, Supporting Information). However, the interaction of STAMBPL1 and TRIM21 was not detected in KIRC cell lines (786‐O, 769‐P and Caki‐1) (Figure S18E, Supporting Information). In addition, overexpression of STAMBPL1 in KIRC cells attenuated the interaction between TRIM21 and AXL, conversely, STAMBPL1 KD promoted TRIM21 binding to AXL (Figure 4K; Figure S18F, Supporting Information). Of note, STAMBPL1 silencing failed to downregulate AXL protein level in TRIM21 KD 786‐O cells, implying that STAMBPL1 KD‐induced AXL ubiquitination and degradation was dependent on TRIM21 (Figure 4L). Collectively, these data indicate that STAMBPL1 blocks TRIM21's recognition of AXL, thus protecting AXL from TRIM21‐dependent degradation (Figure 4M).

### 2.5 Targeting STAMBPL1 Suppresses Metastasis of KIRC Cells

**Figure 5:**
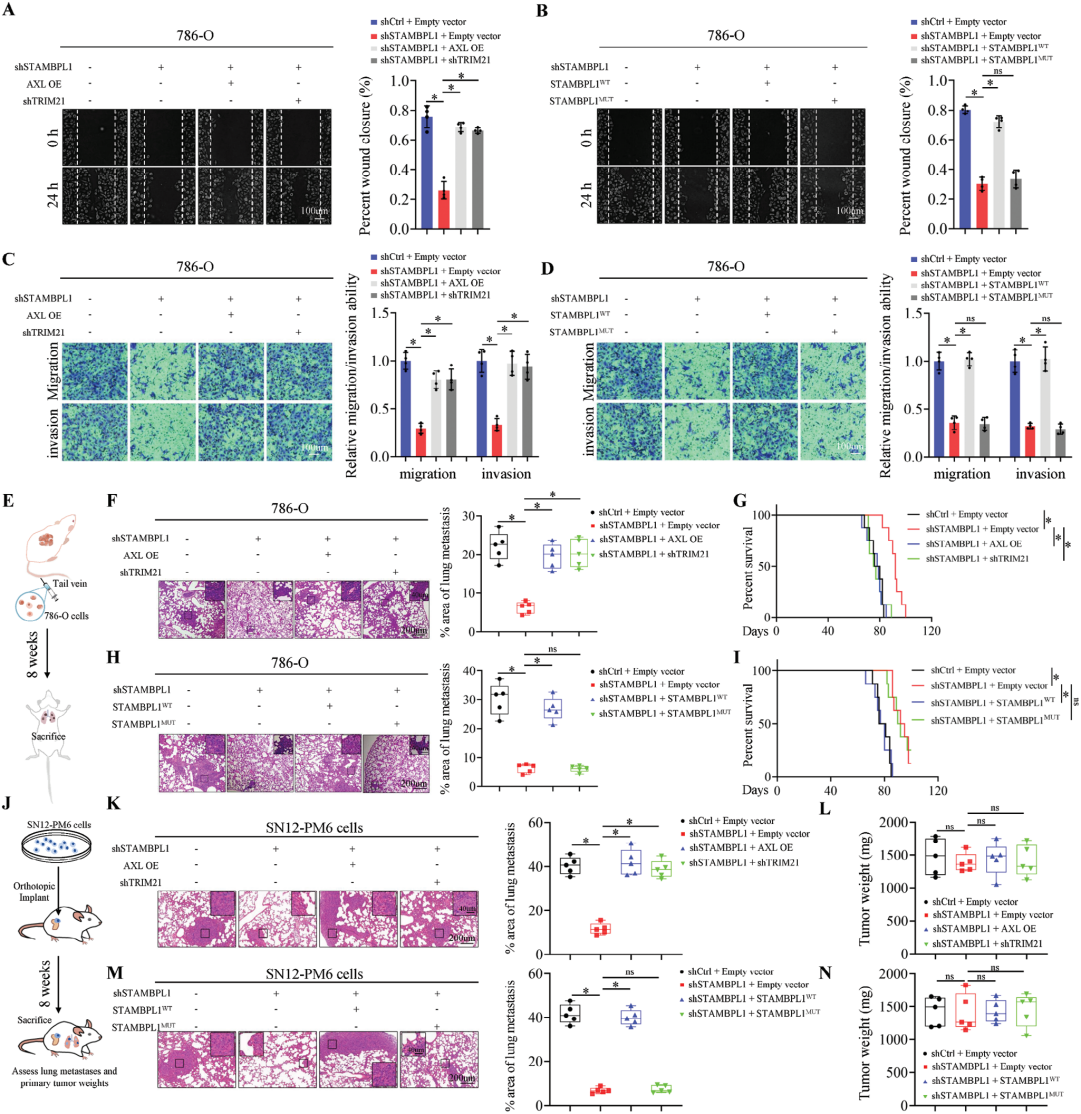
Targeting STAMBPL1 suppresses metastasis of KIRC cells. A,B) Representative images and the quantitative results of wound healing assay (n = 4). C,D) Representative images and the quantitative results of transwell assay (n = 4). E) Schematic diagram illustrating the establishment of a tail‐vein cancer metastasis model. F, H) Representative images and the quantitative results of lung metastases in mice from the different groups (n = 5). G,I) Kaplan–Meier survival curves for mice in the indicated groups (n = 8). J) Schematic diagram illustrating the establishment of an orthotopic xenograft tumor model. K,M) Representative images and the quantitative results of lung metastases in mice from the different groups (n = 5). L,N) The orthotopic tumor mass in the respective groups (n = 5). All data are represented as mean ± SD, and analyzed using one‐way ANOVA followed by Tukey post hoc test. For the analysis in (G,I), log‐rank test was conducted. *p<0.05; ns, not significant; WT, wild type; Mut, mutant; Ctrl, control; OE, overexpressing.

Metastasis, the primary contributor to mortality in kidney cancer, remains obstacle for the substantial treatment, and uncovering the underlying mechanism of this process will provide novel therapeutic strategy for mRCC patients.^[^ ^47^ ^]^ Previous studies have demonstrated that the EMT developmental program and mesenchymal phenotype could be hijacked by cancer cells to facilitate invasion and initiate metastasis.^[^ ^48^ ^]^ Given the significant role of STAMBPL1 in mesenchymal phenotype and immune evasion of KIRC cells, we next examined the impact of STAMBPL1 on tumor metastasis. As expected, STAMBPL1 KD diminished KIRC cells migration and invasion in vitro (**Figure** **5**A–D; Figure S19A–D, Supporting Information). In keeping with the antagonistic role of TRIM21, TRIM21 KD abolished the STAMBPL1 KD‐driven the reduced migration and invasion of KIRC cells, again, STAMBPL1 KD‐impeded invasiveness of KIRC cells were largely rescued by AXL expression as well (Figure 5A,C; Figure S19A,C, Supporting Information). Furthermore, overexpression of STAMBPL1 wild‐type but not the catalytically inactive STAMBPL1 (E292A&D360A) restored the impaired migration and invasion phenotype in STAMBPL1 KD KIRC cells (Figure 5B,D; Figure S19B,D, Supporting Information), which was consistent with our results that STAMBPL1‐E292A&D360A did not rescue AXL protein levels in STAMBPL1 KD cells (Figure 2E).

To further elucidate the impact of the STAMBPL1/TRIM21/AXL axis on metastasis in vivo, we constructed the tail‐vein cancer metastasis model using 786‐O sublines in BALB/c nude mice (Figure 5E). In agreement with in vitro findings, STAMBPL1 silencing dramatically inhibited the metastatic seeding ability of KIRC cells in nude mice (Figure 5F), and the mice injected with STAMBPL1 KD cells displayed better overall survival rates compared to those injected with control cells (Figure 5G). These STAMBPL1 KD effects could be largely rescued by either AXL expression or TRIM21 KD (Figure 5F,G). Consistently, the inhibitory effect of STAMBPL1 KD on tumor metastasis was also confirmed in NOD‐SCID mice using an orthotopic xenograft tumor model, in which AXL expression or TRIM21 KD reversed the effect (Figure 5J,K), meanwhile, the orthotopic tumor mass within the respective groups did not exhibit apparent differences (Figure 5L). Similarly, overexpression of STAMBPL1 wild‐type, but not the enzyme‐dead mutant significantly restored tumor metastasis inhibition by STAMBPL1 KD, indicating STAMBPL1 promoted tumor progression in a DUB activity‐dependent mechanism. (Figure 5H,I,M,N). Immunoblotting assay was performed to confirm the protein level of STAMBPL1, TRIM21 and AXL in excised orthotopic tumor (Figure S20A,B, Supporting Information). Taken together, these results suggest that STAMBPL1/TRIM21/AXL axis represents a promising therapeutic target to suppresses metastasis of KIRC cells.

### 2.6 STAMBPL1 Increases PD‐L1 Levels via Stimulating p65 Nuclear Translocation and Silencing STAMBPL1 Potentiates the Antitumor Efficacy of PD‐1 Blockade

**Figure 6:**
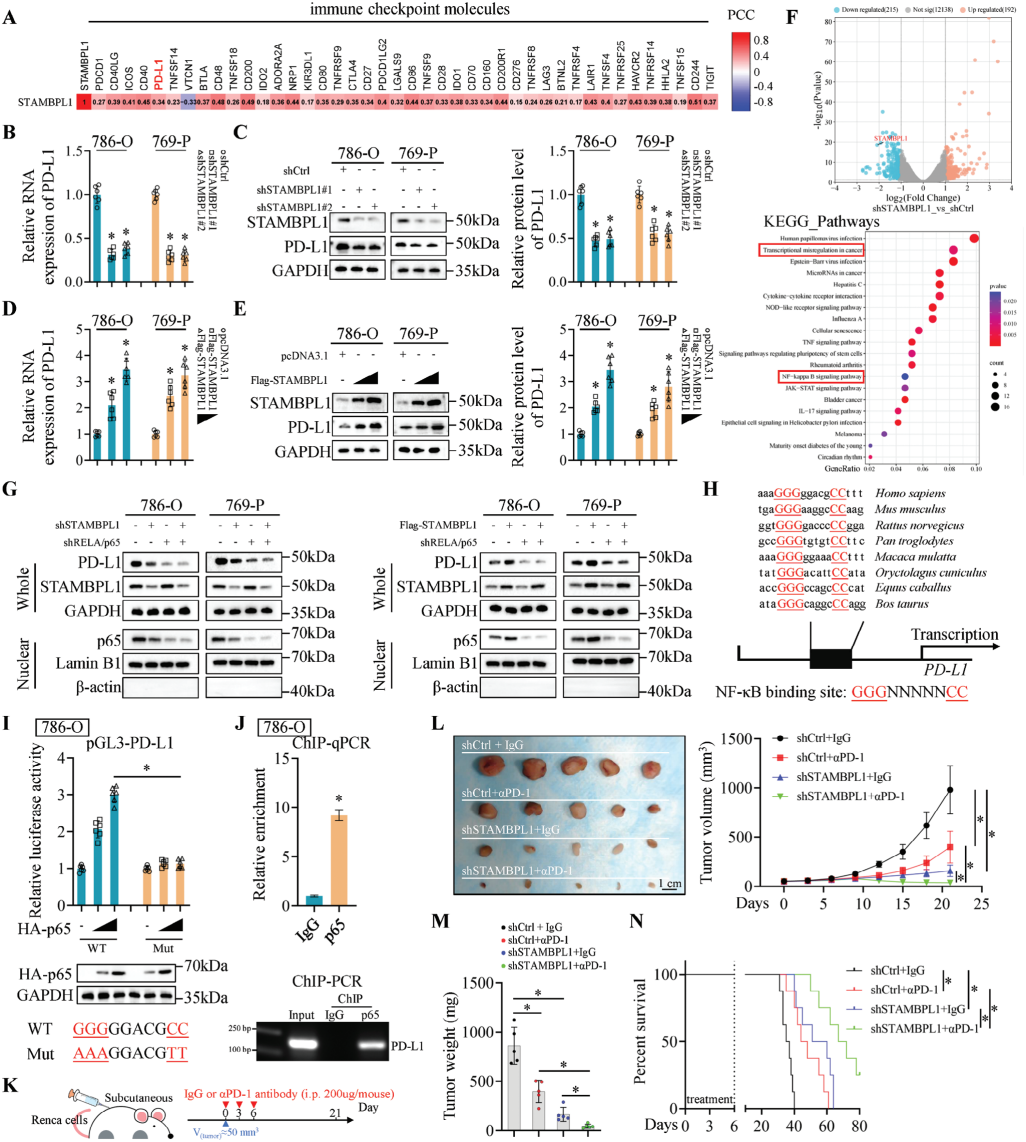
STAMBPL1 increases PD‐L1 levels via stimulating p65 nuclear translocation and silencing STAMBPL1 potentiates the antitumor efficacy of PD‐1 blockade. A) Pearson correlation analysis of STAMBPL1 expression and immune checkpoint molecules mRNA levels in TCGA‐KIRC cohort. B–E) RT‐qPCR and Immunoblotting assays were used to analyze the effect of STAMBPL1 on PD‐L1 expression levels in 786‐O and 769‐P cells (n = 6). F) Volcano plot of DEGs in control and STAMBPL1 KD 786‐O cells. Significantly upregulated (red) and downregulated (blue) genes in STAMBPL1 KD cells are indicated (top panel). KEGG pathway analysis of DEGs between control and STAMBPL1 KD 786‐O cells (bottom panel) (n = 3). G) Immunoblotting analysis of WCL and nuclear lysates derived from KIRC cells infected with the indicated lentiviruses (n = 3). H) A conserved NF‐κB binding site was identified in the PD‐L1 promoter across different species. I) Effects of p65 overexpression on relative luciferase activities of pGL3‐PD‐L1‐WT/Mut vectors in 786‐O cells (n = 6) (top panel). The protein level of p65 was analyzed by immunoblotting (bottom panel). J) ChIP‐qPCR and ChIP‐Semi‐quantitative PCR analysis of p65 occupancy on PD‐L1 promoter in 786‐O cells (n = 3). K) A schematic treatment plan for tumor‐bearing BALB/c mice. Renca cells infected with lentiviruses carrying shNC or shSTAMBPL1 were subcutaneously injected into the left flank of mice, and these mice were treated with anti‐PD‐1 or IgG at a dose of 200 µg per mouse via intraperitoneal injection on days 0, 3, and 6. L) Tumor image and tumor growth curve (n = 5). M) Tumor mass (n = 5). N) Kaplan–Meier survival curves for tumor‐bearing BALB/c mice with indicated treatments (n = 8). All data are represented as mean ± SD, and analyzed using one‐way ANOVA followed by Tukey post hoc test. For the analysis in (J), an unpaired two‐tailed Student′s t test was performed. For the analysis in (N), log‐rank test was conducted. *p<0.05; Ctrl, control; WT, wild type; Mut, mutant; i.p., intraperitoneal.

Our results above demonstrated that inhibition of STAMBPL1 dramatically upregulated multiple immune response genes that might enhance anti‐tumor immunity. Accordingly, we hypothesized that STAMBPL1 could be involved in the regulation of cancer immunotherapy. The use of ICIs in cancer immunotherapy has revolutionized the management of advanced‐stage cancers,^[^ ^49^ ^]^ and ICIs that specifically target the programmed cell death‐1 receptor (PD‐1) have shown significant survival benefits in a subset of patients with KIRC.^[^ ^50^ ^]^ However, the mechanisms driving resistance to PD‐1/PD‐L1 blockade are poorly understood. Interestingly, interrogation of TCGA‐KIRC cohort revealed that STAMBPL1 expression significantly correlated with several immune checkpoint molecules, including PD‐L1 (**Figure** **6**A). To confirm the role of STAMBPL1 in PD‐L1 expression in KIRC, we examined the mRNA level and protein abundance of PD‐L1 after STAMBPL1 KD in 786‐O cells and 769‐P cells. As shown in Figure 6B,C and Figure S22A (Supporting Information), stable STAMBPL1 KD led to decreases in the PD‐L1 mRNA and protein levels. However, STAMBPL1 had no effect on the expression of other immune inhibitory ligands (Figure S21A,B, Supporting Information). Cell surface PD‐L1 on 786‐O or 769‐P cells was also significantly downregulated with STAMBPL1 KD (Figure S22B, Supporting Information). Conversely, ectopic expression of STAMBPL1 resulted in a dramatic upregulation of PD‐L1 in KIRC cell lines (Figure 6D,E; Figure S22A,B, Supporting Information). Importantly, as a member of DUBs, STAMBPL1 failed to affect PD‐L1 ubiquitination levels and no direct STAMBPL1–PD‐L1 interaction was observed in KIRC cells (Figure S23A,B, Supporting Information). These results suggest that STAMBPL1 may not regulate PD‐1 expression via deubiquitination at the post‐translational level.

To determine the molecular basis through which STAMBPL1 regulates PD‐L1 in KIRC cells, we re‐analyzed RNA‐seq results for control and STAMBPL1 KD 786‐O cells. Kyoto Encyclopedia of Genes and Genomes (KEGG) pathway enrichment analysis revealed that STAMBPL1 was involved in modulating transcriptional misregulation in cancer and NF‐kappa B signaling pathway (Figure 6F). As several lines of study have suggested that NF‐kappa B signaling could participate the gene transcription of PD‐L1 to stimulate its expression,^[^ ^51^ ^]^ we wondered whether STAMBPL1 promoted PD‐L1 expression through the NF‐kappa B signaling at the transcriptional level. NF‐κB (nuclear factor kappa‐light‐chain‐enhancer of activated B cells) is a transcriptional factor supporting the neoplastic process, and nuclear translocation of the subunit RELA/p65 is essential for NF‐κB signaling.^[^ ^52^ ^]^ As depicted in Figure 6G, the silencing of STAMBPL1 resulted in a decrease in the nuclear translocation of RELA/p65, whereas the overexpression of STAMBPL1 facilitated the accumulation of nuclear RELA/p65. Furthermore, we also discovered that RELA/p65 silencing diminished the changes in PD‐L1 expression induced by KD or overexpression of STAMBPL1, which confirmed the significance of NF‐kappa B signaling in STAMBPL1‐mediated increase of PD‐L1 levels in KIRC cells (Figure 6G). Intriguingly, AXL stimulated RELA/p65 nuclear translocation and PD‐L1 expression as well, and AXL KD could abolished the ability of STAMBPL1 to initiate NF‐kappa B signaling, demonstrating the requirement for AXL in STAMBPL1‐induced NF‐kappa B signaling activation, which was consistent with the recent report showing that NF‐kappa B signaling activity was associated with AXL expression^[^ ^53^ ^]^ (Figure S23C–E, Supporting Information). Next, we need to figure out the specific mechanism by which AXL regulated NF‐κB activity, determining if it could promote RELA/p65 nuclear translocation by interacting with p65 or its upstream molecules. Co‐IP confirmed AXL interacted with PI3K but not with other NF‐κB signaling proteins, including IKKα, IKKβ, NEMO, TRAF2, TRAF5, TAK1, TAB1, and AKT (Figure S24A,B, Supporting Information). Then, we revealed that PI3K‐inhibitor LY294002 treatment diminished the changes in PD‐L1 expression and RELA/p65 nuclear translocation induced by overexpression of STAMBPL1 in KIRC cells (Figure S24C, Supporting Information). As a classical downstream target of PI3K, AKT was found to interact with the IKKα, with the phosphorylation of IKKα being a crucial process for the activation of the NF‐κB signaling pathway and the nuclear translocation of RELA/p65^[^ ^54^ ^]^ (Figure S24D, Supporting Information). To confirm the role of the PI3K/AKT signaling axis in the regulation of the NF‐κB activity by STAMBPL1/AXL, we used LY294002 or the AKT inhibitor MK2206 to treat cells and found that STAMBPL1 overexpression facilitated the phosphorylation of IKKα at threonine 23 rather than serine 176, while LY294002 or MK2206 treatment could abolish the enhancing effect of STAMBPL1 on the phosphorylation of IKKα at threonine 23 (Figure S24E, Supporting Information). In aggregate, the above results revealed that the NF‐κB activation and upregulation of PD‐L1 induced by STAMBPL1/AXL were primarily achieved through the PI3K/AKT/IKKα axis.

To examine our hypothesis, our subsequent investigation will focus on how STAMBPL1 and NF‐kappa B signaling influence PD‐L1 levels at the transcriptional level. Sequence analysis identified a conserved consensus sequence across multiple species, a well‐accepted NF‐κB binding site,^[^ ^55^ ^]^ in the PD‐L1 gene promoter (Figure 6H). To identify whether p65 specifically binds to the PD‐L1 promoter for direct transcriptional activation, we constructed luciferase reporter plasmids containing the wild‐type or mutant human PD‐L1 promoter, and HA‐tagged p65‐expressing constructs enhanced the luciferase reporter activity of wild‐type PD‐L1 promoter but not mutated promoter, in which the putative binding site for NF‐κB was mutated (Figure 6I; Figure S25A, Supporting Information). Chromatin immunoprecipitation (ChIP)‐qPCR and ChIP‐Semi‐quantitative PCR assays further confirmed that endogenous p65 was recruited to the regions containing the binding site on the PD‐L1 promoter in KIRC cells (Figure 6J; Figure S25B, Supporting Information). The data suggested that p65 promoted PD‐L1 gene transcription by binding to its gene promoter. Consistent with the prior observations that STAMBPL1/AXL activated PD‐L1 transcription by promoting RELA/p65 nuclear translocation, silencing of STAMBPL1 or AXL reduced the enrichment of p65 in the PD‐L1 promoter region, and a remarkable increase in the recruitment of p65 was observed on the region of PD‐L1 promoter in STAMBPL1‐ or AXL‐overexpressing KIRC cells (Figure S25C,D, Supporting Information). Notably, overexpression of AXL could restore p65 enrichment on the PD‐L1 promoter in STAMBPL1 KD cells (Figure S25E, Supporting Information). Therefore, we concluded that STAMBPL1 influenced the PD‐L1 transcription via STAMBPL1/AXL/p65 axis.

To assess the role of STAMBPL1 in the efficacy of immunotherapy in renal cancer, we constructed subcutaneous tumor‐bearing mice models using control Renca cells or STAMBPL1 KD Renca cells and those mice were administrated the anti‐PD‐1 antibody (αPD‐1) (Figure 6K). Strikingly, STAMBPL1 silencing synergistically augmented the antitumor effect of PD‐1 blockade, since immunocompetent mice bearing STAMBPL1 KD‐Renca tumor exhibited suppressed tumor growth and improved overall survival rates upon αPD‐L1 therapy, compared to the mice bearing control‐Renca tumor following αPD‐L1 treatment (Figure 6L–N). Moreover, the body weight of mice was detected every 3 days to evaluate toxicity of αPD‐L1 or STAMBPL1 KD, and there was no substantial weight loss in the treated mice (Figure S25F, Supporting Information). Consistent with the in vitro findings, silencing STAMBPL1 also resulted in AXL degradation, suppression of NF‐κB signaling, and upregulation of CXCL9/10 and HLA‐A/B in vivo (Figure S26A–D, Supporting Information). IF analyses showed that STAMBPL1 silencing stimulated the intratumoral infiltration of CD8+ T cells, which was further enhanced by αPD‐1 treatment (Figure S27A,B, Supporting Information). There were no significant changes in CD4+T cells (marked by CD4), macrophages (marked by F4/80), and Tregs (marked by FOXP3) after STAMBPL1 inhibition (Figure S27A,B, Supporting Information). Notably, the tumor cell and peripheral blood mononuclear cells (PBMCs) coculture assay demonstrated that decreased STAMBPL1 expression could lead to enhanced CD8+ T cell–mediated cytotoxicity (Figure S27C,D, Supporting Information). Collectively, these results revealed that STAMBPL1 could transactivate PD‐L1 expression by facilitating nuclear translocation of RELA/p65 and STAMBPL1 inhibition might sensitize RCC to the anti‐PD‐1/PD‐L1 immunotherapy.

### 2.7 STAMBPL1 Promotes Sunitinib Resistance in an AXL‐Dependent Manner in KIRC

**Figure 7:**
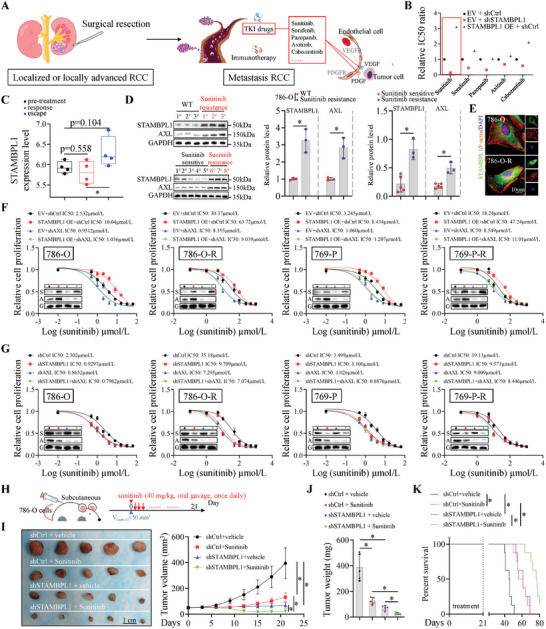
STAMBPL1 promotes sunitinib resistance in an AXL‐dependent manner in KIRC. A) Treatment strategy for RCC. B) The 786‐O cells were infected with lentiviruses carrying specified shRNAs for 72 h, followed by transfection with STAMBPL1 plasmids or empty vector for 24 h. Then the cells were treated with a series of concentrations of TKIs as indicated and the IC50 values of indicated TKIs in each group were measured using the CCK‐8 assay (n = 3). C) Expression levels of STAMBPL1 during sunitinib pretreatment (n = 4), response (n = 4), and resistance (escape; n = 4) phases. D) Immunoblotting analysis of the WCL of 786‐O and 786‐O‐R cells (top left panel) (n = 3). The protein levels of STAMBPL1 and AXL from KIRC patients with (n = 3) or without (n = 5) sunitinib resistance were examined by immunoblotting (bottom left panel). Quantitative results (right panel). E) STAMBPL1 protein level in 786‐O cell line or 786‐O‐R cell line was further examined by IF staining (n = 3). F, G) 786‐O, 786‐O‐R, 769‐P, and 769‐P‐R cells infected with the indicated lentiviruses were treated with a serial dose of sunitinib for 24 h and the IC50 values of sunitinib in each group were measured using the CCK‐8 assay (n = 3) (S: STAMBPL1; A: AXL; G: GAPDH). H) A schematic treatment plan for tumor‐bearing BALB/c nude mice. Control and STAMBPL1 KD 786‐O cells were subcutaneously injected into the left flank of nude mice, and these mice were treated with vehicle or sunitinib (40 mg kg^-1^ per day) by oral gavage. I) Tumor image and tumor growth curve (n = 5). J) Tumor mass (n = 5). K) Kaplan–Meier survival curves for tumor‐bearing BALB/c nude mice with indicated treatments (n = 8). All data are represented as mean ± SD, and analyzed using one‐way ANOVA followed by Tukey post hoc test. For the analysis in (D), an unpaired two‐tailed Student′s t test was performed. For the analysis in (K), log‐rank test was conducted. *p<0.05; Ctrl, control; WT, wild type; EV, empty vector; OE, overexpressing.

Surgical resection remains the standard of care for patients with low‐risk localized RCC (**Figure** **7**A), however, a significant number of RCC patients are usually accompanied by either regional or distant metastases. Currently, first‐line management for mRCC includes immunotherapy (eg, nivolumab) and TKIs targeted therapy (eg, sunitinib)^[^ ^56^ ^]^ (Figure 7A). The above findings have indicated that STAMBPL1 ablation was related to the efficacy of immunotherapy, and we then investigated whether STAMBPL1 modulated the sensitivity of KIRC to TKIs targeted therapy. A drug‐screening assay in 786‐O cells was performed and we discovered that the half maximal inhibitory concentration (IC50) value of sunitinib is most significantly influenced by STAMBPL1 (Figure 7B). In addition, transcriptome analysis (using the GSE76068 dataset) showed that STAMBPL1 was upregulated in a TKI‐resistant RCC patient‐derived xenograft (PDX) mouse model compared to the PDX model response to sunitinib (Figure 7C). In keeping with this notion, sunitinib‐resistant KIRC tissues derived from patients exhibited elevated levels of STAMBPL1 and AXL proteins compared with sunitinib‐sensitive KIRC tissues (Figure 7D). To further characterize sunitinib resistance in KIRC, sunitinib‐resistant KIRC cell lines (786‐O‐R, 769‐P‐R and Caki‐1‐R) were established by chronic exposure to an increasing dose of sunitinib (2‐10 µmol L^−1^) for ten months (Figure S28A–D; Figure S29A–D, Supporting Information). Remarkably, among the drug‐resistant KIRC cell lines we generated, 786‐O‐R cells displayed highest resistance to sunitinib treatment, as indicated by higher IC50 values (Figure S29E, Supporting Information). Consistently, 786‐O‐R cells exhibited enhanced proliferation in response to sunitinib treatment compared to the parental cells, meanwhile, there was no significant difference in proliferation between 786‐O‐R cells and 786‐O cells not treated sunitinib (Figure S28B,E,F, Supporting Information). As expected, upregulated STAMBPL1 and AXL were also observed in sunitinib‐resistant KIRC cell line (Figure 7D,E). To elucidate the effect of STAMBPL1 on sunitinib resistance, sunitinib‐sensitive and resistant KIRC cell lines were applied to loss or gain of function study. Importantly, we noticed that STAMBPL1 KD contributed to enhancing the sensitivity of sunitinib in these cell lines, while ectopic overexpression of STAMBPL1 increased sunitinib resistance of the tumor cells (Figure 7F,G; Figure S30A,B, Supporting Information). Nonetheless, the changes in sunitinib IC50 values induced by STAMBPL1 in KIRC cells could be attenuated by AXL KD (Figure 7F,G; Figure S30A,B, Supporting Information), indicating STAMBPL1 modulated the sensitivity of renal cancer cells to sunitinib through AXL, which was consistent with a previous report that the inhibition of AXL receptor overcomes resistance to sunitinib therapy in renal cell carcinoma.^[^ ^57^ ^]^ Analogous results were observed in vivo xenograft tumor model, STAMBPL1 silencing synergistically enhanced the antitumor effect of sunitinib without obvious toxicity (Figure 7H–K; Figure S31A, Supporting Information). Similarly, shRNA‐mediated STAMBPL1 KD also induced AXL degradation, inhibition of NF‐kB signaling and upregulation of CXCL9/10 and HLA‐A/B in vivo, corroborating in vitro data (Figure S31B–D, Supporting Information). Analysis of infiltrated immune cells demonstrated that STAMBPL1 ablation could significantly increase the infiltration of CD8+ T cells, but not the CD4+ cells, macrophages and Tregs (Figure S32A,B, Supporting Information). All these findings suggest that STAMBPL1 maintains sunitinib resistance via an AXL‐dependent mechanism and that silencing STAMBPL1 may increase the efficacy of sunitinib therapy.

Targeting the STAMBPL1/AXL axis significantly enhances the efficacy of sunitinib treatment and immunotherapy, hence it is worth exploring whether STAMPBP1 expression is associated with the prognosis in patients with renal cancer. As shown in Figure S32C,D (Supporting Information), high STAMBPL1 expression was associated with reduced overall survival and disease‐free survival of these patients.

## 3 Discussion

The advent of immunotherapy has advanced the treatment landscape of RCC and ICIs plus TKIs targeted therapy combinations were approved as the first‐line setting for mRCC. Despite these approvals, a considerable proportion of patients with RCC exhibit limited responsiveness to immunotherapy, highlighting the need for advancements in combination approaches that specifically target immune evasion.^[^ ^2b^ ^]^ Previous studies have revealed that the mesenchymal phenotypes associate with immune suppression and resistance to immunotherapy.^[^ ^58^ ^]^ Thus, a comprehensive understanding of the regulation of mesenchymal phenotypes in KIRC may help elucidate immune evasion mechanisms and develop more effective combination treatment for mRCC. As a member of the JAMM family of DUBs, STAMBPL1 (also known as AMSH‐LP) specifically cleaves K63‐linked polyubiquitin on the substrates and plays a role in the regulation of mesenchymal phenotypes.^[^ ^19^, ^21^ ^]^ Consistently, in this study, STAMBPL1 was identified as a potential mesenchymal maintenance regulator in KIRC cells, whose silencing decreased mesenchymal markers level and induced immune response genes expression.

Mechanistically, we found that STAMBPL1 positively modulated mesenchymal and immune evasion phenotypes of cancer cells largely through removing ubiquitination on AXL, one member of TAM receptors. In contrast to previous reports that ubiquitin E3 ligase CBL mediates AXL ubiquitination and degradation,^[^ ^39^ ^]^ we identified TRIM21 interacted with AXL and promoted the K63‐linked ubiquitination of AXL in KIRC cells. STAMBPL1 binds to AXL IC region, which also harbors the TRIM21 recognition domain. Thus, STAMBPL1 binding to AXL will sterically block TRIM21 from recognizing the AXL and inhibit the subsequent ubiquitination of AXL. Our research and previous studies consistently demonstrate the crucial role of AXL in the tumor mesenchymal phenotype and immune evasion,^[^ ^36^, ^59^ ^]^ indeed, the small molecule inhibitors targeting AXL are currently under clinical evaluation, however, Markus et al. showed that AXL inhibitors impaired ubiquitination and degradation of AXL leading to accumulation of the AXL receptor on the cellular surface, and this might increase tumor proliferation.^[^ ^60^ ^]^ To address this challenge, Rui et al. reported the discovery of new AXL Proteolysis targeting chimera (PROTAC) degraders to efficiently downregulated AXL protein without causing surface accumulation of AXL receptor.^[^ ^61^ ^]^ PROTACs represent exogenous molecular tools that consist of chemically synthesized compounds,^[^ ^62^ ^]^ we herein identified an endogenous molecular target, STAMBPL1, which affects the degradation of AXL. Targeting STAMBPL1 promotes AXL ubiquitination and suppresses its surface accumulation. Nevertheless, whether degradation of AXL endogenously would be a superior strategy for KIRC patients comparing with the AXL inhibitor or PROTAC degraders therapy requires further investigation.

Multiple mechanisms exist through which RCC evades the immune system and immune checkpoint signaling represents a crucial subsection among them.^[^ ^2b^ ^]^ Our results showed that STAMBPL1 led to an increase in mRNA and protein level of PD‐L1 in KIRC cells mainly through promoting RELA/p65 nuclear translocation and NF‐kappa B signaling activation, as nuclear p65 could directly bind to PD‐L1 promoter to stimulate its transcription. Moreover, our study identified a combined therapeutic strategy that STAMBPL1 silencing synergistically enhanced the antitumor effect of PD‐1 blockade. In contrast, findings from Wenjun et al. indicated that elevated PD‐L1 protein levels enhanced the efficacy of anti‐PD‐1/PD‐L1 immunotherapy,^[^ ^22^ ^]^ whereas Wei et al. revealed that RRM2 inhibition reduced PD‐L1 levels and augmented the antitumor efficacy of αPD‐1 in renal cancer.^[^ ^63^ ^]^ Notably, the exact association between PD‐L1 levels and PD‐1/PD‐L1 blockade therapy response in KIRC remains unclear. Initial trials suggested that pretreatment tumor PD‐L1 expression was correlated with response to anti‐PD‐1/PD‐L1 therapies in RCC, yet, a majority of patients with PD‐L1(+) tumors did not respond to the treatment and a considerable number of patients classified as PD‐L1‐negative still benefit from the treatment.^[^ ^64^ ^]^ Therefore, many observations have questioned the prospect of using PD‐L1 expression for predicting therapeutic activity of PD‐1/PD‐L1 blockade. Here, we defined STAMBPL1 as a modulator for the efficacy of anti‐PD‐1/PD‐L1 immunotherapy in KIRC, however, further investigation is required to elucidate the mechanisms by which STAMBPL1 regulates αPD‐1 treatment sensitivity. Additionally, the role of decreased PD‐L1 expression levels in the process of STAMBPL1 inhibition enhancing αPD‐1 therapeutic efficacy warrants further exploration as well.

Considering STAMBPL1's involvement in PD‐L1 transcription and immune evasion, targeting the STAMBPL1/AXL axis holds significant clinical implications for KIRC therapy. Specifically, disruption of the STAMBPL1/AXL axis will provide therapeutic benefits by reducing mesenchymal phenotypes and enhancing tumor immunogenicity. Importantly, STAMBPL1 inhibition could effectively mitigate AXL signaling and associated immune escape pathways, thus enhancing antitumor immune responses and overcoming immunotherapy resistance.

TKIs, including sunitinib, were approved as a first‐line therapy for patients with mRCC.^[^ ^65^ ^]^ Nonetheless, a substantial proportion of patients ultimately develop sunitinib resistance after 6–15 months of treatment.^[^ ^66^ ^]^ Sunitinib, an oral multitarget RTK inhibitor, exerts potent anti‐angiogenic effects and direct anti‐tumor activities by targeting vascular endothelial growth factor receptor (VEGFR), platelet‐derived growth factor receptor (PDGFR), stem cell growth factor receptor, and FMS‐like tyrosine kinase 3.^[^ ^67^ ^]^ The results from our study suggested that STAMBPL1 was upregulated in sunitinib‐resistant KIRC cell line and sunitinib‐resistant KIRC tissues. Inhibition of STAMBPL1 could overcome sunitinib resistance in KIRC through AXL. Zhou et al. reported that AXL activated its downstream kinases AKT and ERK to maintain sunitinib resistance in RCC.^[^ ^57^ ^]^ Apart from this, there must be other novel downstream target proteins of STAMBPL1/AXL contributing to the regulation of sunitinib sensitivity in KIRC. Hence, the further investigation elucidating the mechanism by which STAMBPL1/AXL regulates resistance to sunitinib in renal cancer was needed in the future.

The RNA‐sequencing analysis of STAMBPL1 KD KIRC cells revealed additional targets that might contribute to mesenchymal and immune evasion phenotypes (Figure 1E). Further investigation is warranted to determine whether these targets interact with the STAMBPL1/TRIM21/AXL axis in KIRC. Previous studies reported that AXL activated AKT pathway to enhance MYC protein accumulation while MYC could reciprocally upregulate AXL expression in breast cancer cells, suggesting a potential interaction between MYC and the STAMBPL1/TRIM21/AXL axis through the AKT signaling pathway in renal cancer.^[^ ^68^ ^]^ Andrea et al. reported that in multiple myeloma cells, AXL could regulate the expression of MICA (an immune response‐related gene) through the NF‐κB signaling pathway, implying a possible similar interaction in renal cancer.^[^ ^69^ ^]^ A comprehensive analysis of the downstream targets of STAMBPL1 presents a challenging yet meaningful endeavor. Further studies to explore the interaction between the additional targets and the STAMBPL1/TRIM21/AXL axis will expand the understanding of the function of STAMBPL1 in tumor progression.

**Figure 8:**
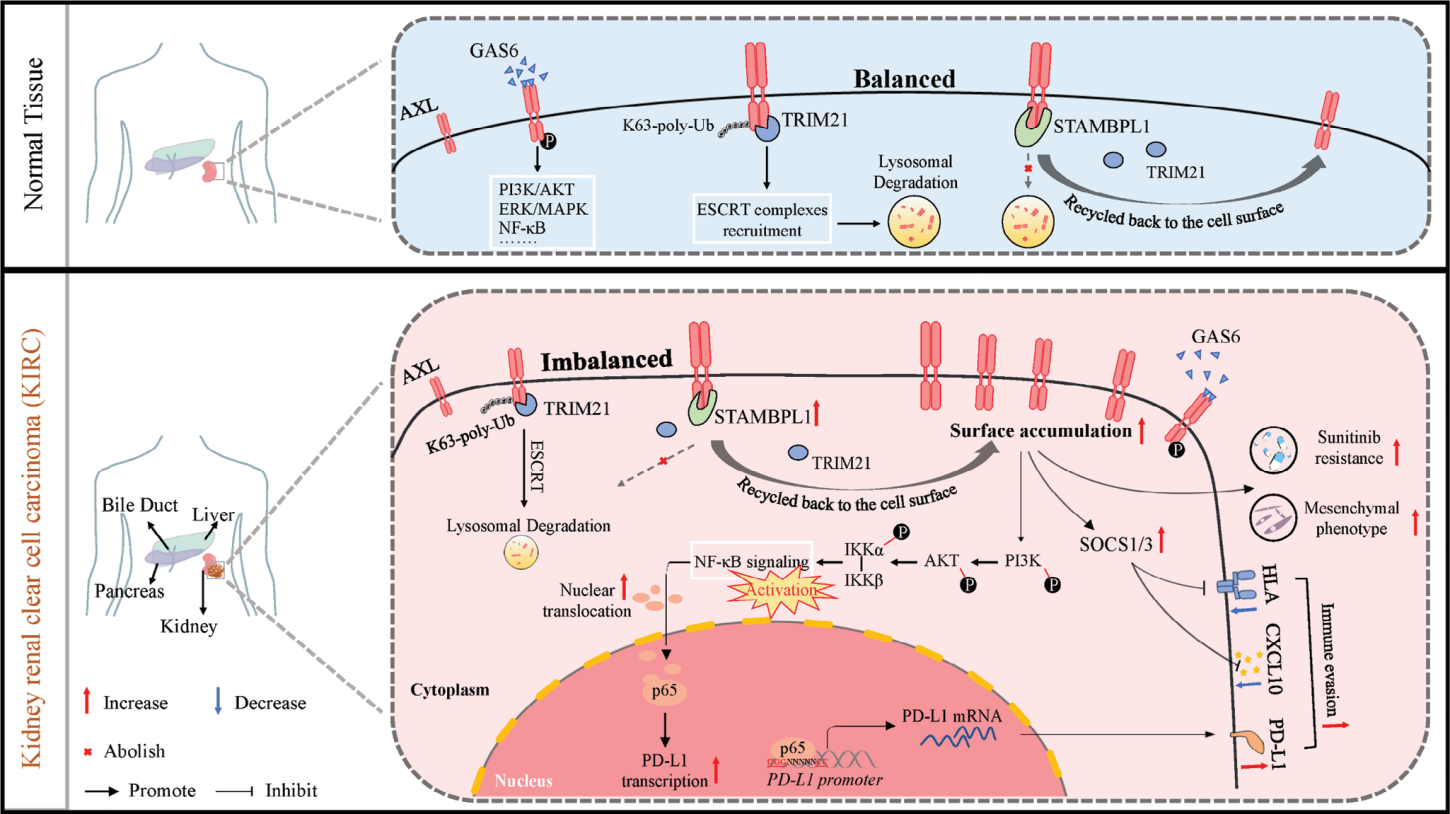
A schematic model where STAMBPL1 elevates protein abundance and surface accumulation of TAM Receptor AXL through protecting AXL from TRIM21‐dependent K63‐linked ubiquitination and subsequent lysosomal degradation, thus enhancing the mesenchymal and immune evasion phenotypes and promoting sunitinib resistance.

Lastly, when investigating the synergistic effects of STAMBPL1 inhibition with αPD‐1 or sunitinib in renal cancer, it is preferable to utilize small molecule inhibitors targeting STAMBPL1 rather than employing STAMBPL1 KD tumor cells. However, potent STAMBPL1 inhibitors have yet to be reported. We believe that our research could provide a preliminary foundation and insights for the development of STAMBPL1 inhibitors. In summary, our study demonstrates that STAMBPL1 protects AXL from TRIM21‐mediated K63‐linked ubiquitination, thereby maintaining mesenchymal phenotypes and immune evasion of KIRC cells (**Figure** **8**). Furthermore, STAMBPL1 inhibition enhances the antitumor effect of anti‐PD‐1 immunotherapy or sunitinib in KIRC. These findings not only provide a molecular insight into STAMBPL1/TRIM21/AXL axis but also reveal a potential therapeutic strategy for mRCC patients.

## 4 Experimental Section

### Cell Culture, Compounds and Antibodies

HEK293T, murine renal cancer cell line Renca and normal kidney tubular epithelial cell line HK‐2 were purchased from American Type Culture Collection (ATCC). Human KIRC cell lines were obtained from the Institute of Basic Medical Sciences Chinese Academy of Medical Sciences, China Infrastructure of Cell Line Resources (Beijing, China). HEK293T, HK‐2 and SN12‐PM6 cells were cultivated in a standard medium comprising DMEM (Procell Life Science & Technology, Wuhan, China), 10% fetal bovine serum (FBS) (GIBCO, Grand Island, NY, USA), and 1% penicillin–streptomycin (Biosharp, Anhui, China). 786‐O, Caki‐1 and 769‐P cells were maintained in RPMI 1640 medium (Procell Life Science & Technology, Wuhan, China) containing 10% FBS, and culture medium of Renca cells was further supplemented with 1% glutamine and 1 mM sodium pyruvate. All cells were maintained at 37 °C with 5% CO2 in a humidified incubator. To establish sunitinib‐resistant KIRC cells, 786‐O, 769‐P and Caki‐1 cells were cultured with an increasing dose of sunitinib (2‐10 µmol L^−1^). Following ten months of culture, sunitinib‐resistant KIRC cells were generated.^[^ ^70^ ^]^ CHX (HY‐12320) and MG132 (HY‐13259) were purchased from MedChemExpress. Chloroquine (#S6999), Sunitinib (#S7781), LY294002 (#S1105) and MK2206 (#S1078) were obtained from Selleck Chemicals. Details on the antibodies used in the study were documented in the Table S1 (Supporting Information).

### Plasmids, shRNAs, and Lentiviruses

Prokaryotic plasmids encoding GST‐STAMBPL1 or GST‐TRIM21 fusion protein were constructed in pGEX‐4T. The PD‐L1 wild‐type or mutant promoter sequence was constructed into pGL3‐basic firefly luciferase reporter vector. Human full‐length or truncated STAMBPL1, TRIM21 and AXL were cloned into the 3×Flag‐tagged pcDNA3.1 vector or 3×HA‐tagged pcDNA3.1 vector (Invitrogen, Carlsbad, CA, USA). Ubiquitin and corresponding derivatives were packed into the pcDNA3.1 plasmid with a HA tag (Thermo Fisher Scientific). Flag‐HRS, STAM1, and STAM2 plasmids were purchased from Sangon Biotech. The p65 coding region was cloned into the vector pCMV with a HA tag (TaKaRa, Shiga, Japan). shRNAs for STAMBPL1, TRIM21, AXL, TRIM28, RELA/p65, TNFAIP3, HRS, STAM1 and STAM2 were cloned into lentiviral pLKO.1 vector. The sequences of the shRNAs were listed in the Table S2 (Supporting Information). The human STAMBPL1 cDNA (wild‐type or E292A&D360A mutant), TRIM21 cDNA, AXL cDNA, SOCS1 cDNA and SOCS3 cDNA were subcloned in lentiviral pCDH vector. The virus particles were generated by co‐transfecting HEK293T cells with recombinant lentivirus vectors and helper plasmids (pMD2.G and psPAX2) following the lentivirus packaging protocol provided by Addgene, and were used to infect target cells in the presence of 8 µg ml^−1^ polybrene.

### RT‐qPCR

RT‐qPCR assay was performed as the previous study.^[^ ^71^ ^]^ Total RNA was extracted with TRIzol (Invitrogen, Carlsbad, CA, USA) and reverse transcribed with the PrimeScript RT Reagent Kit (TaKaRa, Shiga, Japan). RT‐qPCR was performed using SYBR Green PCR master mix (Applied Biosystems) and the Roche LightCycler 480 detection system. Relative gene expression was calculated by the 2^−ΔΔCT^ method with GAPDH as an internal control. The specific primer sequences for RT‐qPCR were listed in Table S3 (Supporting Information).

### Total and Nuclear Proteins Exaction and Immunoblotting

Total proteins were obtained using RIPA buffer (Epizyme, Shanghai, China; PC104), and nuclear proteins were isolated following the instructions provided by the commercial kit (Beyotime, Shanghai, China; P0028). The protein concentration was determined using the BCA protein assay kit (Epizyme, Shanghai, China; ZJ101). Then proteins were separated on 10% SDS‐PAGE gels (50 mg per lane) and transferred to PVDF membranes (Millipore). After blocking with 5% non‐fat milk for 1 h, the membranes were incubated overnight at 4 °C with primary antibodies. Following incubation with HRP‐linked secondary antibodies for 1 h at room temperature, the protein bands were detected by chemiluminescence (Bio‐Rad) and the images were analyzed using Image Lab software.

### RNA Fish

RNA FISH was performed using the specific fluorescently labeled anti‐AXL probes, which were designed and synthesized by Servicebio (Wuhan, China). In brief, cells were seeded onto glass slides and then fixed using 4% paraformaldehyde for 10 min at room temperature. Following fixation, the cells were permeabilized with 0.5% Triton X‐100 for 5 min at 4 °C. After three washes for 5 min, cells were subjected to blocking with a pre‐hybridization buffer for 30 min at 37 °C. Subsequently, the cells were incubated in a hybridization buffer containing AXL probes at 37 °C overnight in darkness. After three times with Wash Buffer I (4 × SSC with 0.1% Tween‐20), once with Wash Buffer II (2 × SSC), Wash Buffer III (1 × SSC) at 42 °C in the dark for 5 min and one wash with 1 × PBS at room temperature, the cells were counterstained with DAPI for 5 min at 37 °C. The images were acquired using a confocal scanning microscope (OLYMPUS, FV1200).

### Cytoplasmic and Nuclear RNA Analysis

Cytoplasmic and nuclear fractions were extracted using the Nuclear and Cytoplasmic Protein Extraction Kit (Beyotime, Shanghai, China) in accordance with the manufacturer's guidelines. RNA was extracted from the separated fractions. The subcellular localization of AXL was determined through RT‐qPCR analysis. U1 was employed as the nuclear RNA marker, while GAPDH served as the cytoplasmic RNA marker.

### Polysome Profiling

Polysome fractionations were conducted as described previously.^[^ ^72^ ^]^ STAMBPL1 KD or control 786‐O cells were treated with 100 µg mL^−1^ CHX for 10 min at 37 °C. Then, cells were harvested and 1 mL of cytoplasmic extract was layered onto 11 mL of 10%–50% sucrose gradient, followed by centrifugation at 36000 rpm for 2.5 h at 4 °C. Gradients were fractionated and monitored at absorbance 254 nm. Collected fractions were then analyzed by RT‐qPCR.

### CHX, MG132, and CQ Treatment

For protein half‐life analysis, the transfected KIRC cells were treated with 100 µg mL^−1^ CHX, collected at the indicated time, and lysed in 2 × SDS buffer. The cell lysates were analyzed with immunoblotting. For proteasome inhibitor MG132 and lysosome inhibitor CQ treatment, the transfected KIRC cells were treated with 10 µM MG132 or 50 µM CQ and harvested at the indicated time points.

### Collection of Clinical Specimens and IHC Analysis

This study was approved by The Ethics Committee of Renmin Hospital of Wuhan University. Clinical specimens of KIRC (with or without sunitinib resistance) were obtained from patients undergoing urological surgery in Renmin Hospital of Wuhan University. Participants had written informed consent and the privacy rights of human subjects always been observed. All procedures were performed in accordance with the principles expressed in 1964 Helsinki declaration. For IHC, the tissue sections were subjected to standard dewaxing and rehydration, followed by treatment with citrate buffer. After blocking with 3% hydrogen peroxide and 10% goat serum, the sections were incubated with specific antibodies targeting STAMBPL1, TRIM21, or AXL at 4 °C overnight and HRP‐conjugated secondary antibodies at 37 °C for an additional 1h. After being visualized with diaminobenzidine, the immunostaining intensity was evaluated and scored by three independent pathologists who were blinded to the clinical information. The staining intensity was scored as follows: 1 = weak staining at 100× magnification with little or no staining at 40× magnification; 2 = moderate staining at 40× magnification; 3 = strong staining at 40× magnification.

### Primary Cancer Cell

Primary cancer cells were isolated from fresh human KIRC tissues according to the procedures in previous studies.^[^ ^73^ ^]^ KIRC tissue specimens were collected into cold DMEM/F12 medium post‐nephrectomy and processed within 12 h. Cancerous tissues were sectioned into 1 mm^3^ fragments and digested using 10 mg mL^−1^ collagenase type II (Gibco; 17 101 015) in 10 mL HBSS (Gibco; 14 025 092) media, supplemented with 10 µM Y‐27632 (Selleckchem; S1049) and 1% penicillin‐streptomycin (PS), for approximately 1 h at 37 °C, actively vortexed every 15 min. After washed 3 times with cold PBS, the cell clumps were digested with TrypLE Express (Gibco; 12 605 010), supplemented with 10 µM Y‐27632 and 1% PS, for another 5 min at 37 °C with constant shaking. Next, primary cancer cells were filtered with 70‐µm cell strainer and plated in 10‐cm Petri dishes with 20% FBS of culture medium. The Petri dishes were incubated with 1.5% Matrigel in 37 °C incubator for 2 h in advance. The culture medium for primary cells comprised DMEM/F12 (Gibco; 11 330 032) supplemented with 10% FBS, 1% GlutaMAX (Gibco; 35 050 061), 1% HEPES (Gibco; 15 630 080), 5 ng mL^−1^ FGF2 (PeproTech; 100–18B), 5 ng mL^−1^ EGF (PeproTech; AF‐100‐15), 5 µg mL^−1^ insulin (Sigma; I9278), 5 µg mL^−1^ transferrin (Sigma; T8158), 25 ng mL^−1^ hydrocortisone (Apexbio LLC; B1951), 10 µM Y‐27632, and 1% PS. All experiments were conducted on cells at the first passage.

### Processing of Peripheral Blood Samples

PBMCs from healthy human donors were isolated using Human Lymphocyte Separation tube (Beijing Dakewe Biotech Company Limited, China). Once PBMCs were obtained from healthy donor blood, CD8+ T cells were isolated using a magnet‐based CD8+ T‐cell isolation kit according to the manufacturer's protocol (StemCell Technologies; 19 053), and the cells were used immediately for experiments.

### Calcein Release Cytotoxicity Assay

Target KIRC cells labeled with Calcein‐AM (Beyotime, Shanghai, China) and human CD8+ T cells were mixed together at indicated target to effector ratios in RPMI medium without FBS and seeded into wells of a 96‐well U‐bottom plate. Saponin (0.1%) or Triton X‐100 (2%) were added to wells that contained only tumor cells to provide a value for “maximum calcein release.” Tumor cell–only wells without any treatment were included to quantify the spontaneous release of calcein, while media‐only wells were used to determine the background levels. The plate was briefly centrifuged at 1000 rpm for 30 s, then incubated at 37 °C for 4 h. After 4‐h incubation, the plate was centrifuged at 2000 rpm for 5 min. Hundred microliters of the 200 µL supernatant was then removed from each well and added to a new, opaque (black) 96‐well flat‐bottom plate. Calcein fluorescence was read using an automated fluorescence measurement system and percent cytotoxicity was calculated using the following formula: % Cytotoxicity = 100 × [(Experimental well release‐Spontaneous release well)/(Maximum release well‐Spontaneous release well)].

### IF Staining

KIRC cells were fixed with 4% paraformaldehyde, permeabilized with 0.1% Triton X‐100, blocked with 5% BSA and incubated with specific primary antibodies, followed by incubation in a solution of fluorescently labeled secondary antibody (1:100 dilution). Then the nuclei were stained with DAPI, and fluorescence images were captured using a confocal scanning microscope (OLYMPUS, FV1200).

### IP

To detect endogenous and exogenous protein interactions, whole‐cell extracts were immunoprecipitated with specific antibody or control serum. The immunoprecipitated complexes were then incubated with protein A/G beads (Epizyme, Shanghai, China). After washing four times with lysis buffer (20 mM Tris‐HCl, 125 mM NaCl, 5 mM MgCl2, 0.2 mM EDTA, and 0.1% NP‐40), the co‐precipitated proteins were analyzed by immunoblotting.

### GST Pull‐Down

The GST‐STAMBPL1 or GST‐TRIM21 fusion proteins were produced in Escherichia coli (BL21) cells and purified using a GST‐Tag Protein Purification Kit (Sangon Biotech, Shanghai, China). The purified GST or GST‐tagged recombinant protein bound to GST beads and incubated with KIRC cell lysates. After washing five times with GST buffer (1 mM EDTA, pH8.0 tris‐HCl, 200 mM NaCl and 1% Triton X‐100), the immobilized proteins were eluted and subjected to immunoblotting analysis.

### MS

STAMBPL1 KD 786‐O cells were lysed in IP buffer and processed for immunoprecipitation with anti‐STAMBPL1 or normal rabbit IgG for 6 h at 4 °C. The immune complexes were incubated with Pierce Protein A/G Magnetic Beads (Thermo Scientific) overnight at 4 °C and washed with IP buffer. After SDS‐PAGE and Coomassie brilliant blue staining, the gel was cut into sections and digested with trypsin. For each sample, 2 µg of total peptides were separated and analyzed with a nano‐UPLC (nanoElute2) coupled to a timsTOF Pro2 mass spectrometer (Bruker) with a nano‐electrospray ion source. Then the raw MS files were processed using PaSER software (Version 2023) and the built‐in DDA‐ProluCID‐GPU search engine.

### Ubiquitination Assay

Cells were transfected with HA‐ubiquitin (HA‐Ub) and indicated constructs. After transfection for 36 h, the cells were collected and lysed in a denaturing lysis buffer (50 mM Tris‐HCl pH 7.4, 150 mM NaCl, 1% NP‐40, and 1% SDS with protease and phosphatase inhibitors), vortexed vigorously for 15–30 min, boiled for 10 min. Then the cell lysates were diluted ten times with SDS‐free lysis buffer and subjected to IP with appropriate antibody and protein A/G magnetic beads followed by immunoblotting analysis.

### Analysis of Membrane Protein by the Flow Cytometry

For cell surface expression of HLA‐A/B/C, AXL and PD‐L1, 1 × 10^6^ KIRC cells were washed once in PBS, detached using Accutase^TM^ cell detachment solution (STEMCELL Technologies, Vancouver, Canada; 0 7920), and stained with 5 µl PE or ABflo® 488 conjugated primary antibody for 20 min at 4 °C in the dark. After staining, samples were washed and analyzed with Beckman CYTOFLEX and Beckman CytExpert Software 2.3.

### ELISA Assay

For the CXCL10 ELISA assay, media from KIRC cells infected with indicated lentivirus were collected and stored at −80 °C until use in subsequent assays. CXCL10 levels in the supernatants were quantitated using a commercially available IP‐10 (CXCL10) Human ELISA Kit (Invitrogen, Carlsbad, CA, USA; KAC2361) in clear flat‐bottomed 96‐well plates following the manufacturer's instructions, and expressed as the amount recovered per 10^6^ cells. Prior to analysis, the collected supernatant was thawed on ice and subjected to centrifugation at 4 °C for 10 min at 14000 rpm to remove cell debris.

### RNA‐seq and Bioinformatic Analysis

Total RNA was extracted from control or STAMBPL1 KD 786‐O cells by using RNAprep Pure Kit (TIANGEN Biotech, Peking, China; DP430) following protocol. The integrity of the total RNA was evaluated with RNA Nano 6000 Assay Kit for the Bioanalyzer 2100 system (Agilent Technologies, CA, USA). RNA‐seq libraries were prepared according to MGIEasy mRNA Library Prep Kit and the indexed libraries were sequenced using MGISEQ‐2000. For analysis of RNA‐seq results, RNA‐seq reads quality was assessed using FastQC (v0.11.9) and aligned to the Homo sapiens reference transcripts by HISAT2 (v2.2.1). DESeq2 R package was conducted to determine the differentially expressed genes (DEGs) with P value < 0.05 and | log2[fold change] | >1, and all DEGs between control and STAMBPL1 KD 786‐O cells were plotted as volcano plot using R package ggplot2 (v3.3.2) and subjected to KEGG enrichment analysis by the R packages clusterProfiler (v4.10.0). GSEA of STAMBPL1 KD RNA‐seq was performed using the GSEA software (v4.1.0).^[^ ^74^ ^]^ Gene sets with nominal P value < 0.05 and false discovery rate (FDR) <0.25 were considered of significance. Mesenchymal and immune response genes were extracted and annotated with their fragments per kilobase million (FPKM) values, z‐score method was used to generated heatmap.

TPM‐normalized mRNA expression data and corresponding clinical information for KIRC patients were obtained from TCGA database (http://www.tcga.org/), and three sets of microarrays were downloaded from the Gene Expression Omnibus (GEO) database (https://www.ebi.ac.uk/arrayexpress/).The prognostic analysis of renal cell carcinoma patients was conducted utilizing the GEPIA database (http://gepia.cancer‐pku.cn).

### ChIP

ChIP assay was performed using human KIRC 786‐O cells. These cells were fixed with 1% paraformaldehyde at room temperature for 10 min and subjected to a Bioruptor Pico Sonifier to shear chromatin DNA. Precleared chromatin was incubated overnight with anti‐p65 or normal rabbit IgG (Cell Signaling Technology, Boston, USA; 2729) at 4 °C. Then the protein G agarose/salmon sperm DNA beads (Roche, Mannheim, Germany) were applied to pull down the antibody–chromatin complexes. After washing thoroughly, the immunocomplexes were processed by reverse cross‐linking and proteinase K digestion. Immunoprecipitated DNA was purified and quantified by Semi‐quantitative PCR or RT‐qPCR analysis with specific primers. The primers 5′‐CATATGGGTCTGCTGCTGAC‐3′ and 5′‐CAACAAGCCAACATCTGAAC‐3′were used to amplify the PD‐L1 promoter sequence. The primers for GAPDH, 5′‐TACTAGCGGTTTTACGGGCG‐3′ and 5′‐TCGAACAGGAGGAGCAGAGAGCGA‐3′, were used as a negative control.

### Luciferase Reporter Assays

To detect luciferase reporter activity, KIRC cells were transfected with 0.8 µg PD‐L1 promoter‐luciferase reporter (pGL3‐PD‐L1‐Luc), 0.2 µg pCMV‐HA‐p65 plasmid, and 50 ng pRL‐TK Renilla in the presence of Lipofectamine 2000 (Invitrogen, Carlsbad, CA, USA). After transfection for 24 h, Dual Luciferase Reporter Assay System (Promega, Madison, WI, USA) was used to measure luciferase activity according to the manufacturer's instructions. Firefly luciferase activities were standardized using Renilla luciferase control value, and relative to values from the empty vector.

### Cell Proliferation Assays

The Cell Counting Kit‐8 (CCK‐8) assay, colony formation assay, and EdU assay were used for the in vitro cell proliferation assay. For CCK‐8 assay, KIRC cells under the indicated conditions were seeded into 96‐well plates. Prior to the assay, the medium was replaced with fresh medium, 10 µl CCK‐8 was added to each well and incubated for additional 2 h. The optical density values at 450 nm were detected via automatic plate reader (PerkinElmer, Waltham, Massachusetts, USA). IC50 was obtained by probit analysis and calculated using GraphPad Prism 8.0 software. For colony formation assay, 786‐O and 786‐O‐R cells were seeded in 6‐well plate (1000 cells per well) and exposed to 2 µM sunitinib. After 14‐day culture, colonies were fixed with 4% paraformaldehyde for 15 min and stained with 0.1% crystal violet for 15 min. The area of colonies was quantified using Image J software (Bio‐Rad Laboratories, California, Hercules, USA). The Click‐iT EdU‐594 Cell Proliferation Kit (Servicebio, Wuhan, China) was utilized for the EdU staining process. In brief, 786‐O and 786‐O‐R cells were plated in 24‐well culture plates at a density of 5 × 10^5^ cells per well and treated with DMSO or sunitinib (2 µM) for 24 h. After incubation with 10 µM EdU for 2 h, the cells were fixed with 4% paraformaldehyde and permeabilized with 0.1% Triton X‐100. Finally, cells were stained with Click Addictive Solution, and DNA was stained with DAPI. Fluorescence microscopy was used to observe the proportion of EdU‐positive cells.

### Cell Migration and Invasion Assays

The wound healing assay and transwell assay were used for the in vitro migration and invasion assays. KIRC cells utilized in the wound healing assay were cultured in six‐well plates until reaching 100% confluency. The cell monolayers were scratched using a pipette tip, and then cells were washed gently to remove cell debris and cultured in serum‐free medium containing 4 µg mL^−1^ mitomycin. The closure area was observed and quantified at 0 and 24 h after wounding. For transwell migration/invasion assays, a total of 1 × 10^5^ KIRC cells in 200 µl of FBS‐free medium were seeded in the upper 8‐µm transwell chamber (Corning, New York, USA) containing an uncoated or Matrigel‐coated membrane. The lower chamber was filled with 600 µl of 20% FBS medium. Twenty‐four hours later, the cells that crossed the inserts were fixed with 4% paraformaldehyde, stained with 0.1% crystal violet, and imaged and counted with an inverted microscope.

### In Vivo Mouse Studies

Animal studies were approved by ethics committee of Renmin Hospital of Wuhan University and carried out in strict accordance with the recommendations of the Guide for the Care and Use of Laboratory Animals of the National Institutes of Health. For the tail vein metastases model, 786‐O cells (1 × 10^6^) infected with the indicated lentiviral particles were injected into the tail vein of 6‐week‐old BALB/c nude mice (n  =  6 per group). After 8 weeks, the mice were euthanized, and their lung tissues were dissected and analyzed by H&E staining. To establish orthotopic xenograft tumor models, 6‐week‐old NOD‐SCID mice were anesthetized with isoflurane (2‐3%) and carefully placed in the left lateral decubitus position. Then a small incision was made in the right flank to expose the kidney. A 24‐gauge needle was used to slowly inject approximately 1 × 10^6^ suspended SN12‐PM6 cells under the kidney capsule. All mice were euthanized at 8 weeks post‐injection, and their kidneys and lungs were subsequently harvested. For xenograft tumor model, 6‐week‐old BALB/c nude mice were subcutaneously inoculated in the left flank with 100 µL of PBS containing 5 × 10^6^ control or STAMBPL1 KD 786‐O cells. Tumor volumes were monitored every 3 days and calculated using the formula (L × W^2^)/2. Upon reaching a tumor size of approximately 50 mm^3^, mice were randomly assigned to different treatment groups and administered either vehicle or sunitinib (40 mg kg^-1^ per day) via oral gavage. At the end of the 21‐day treatment period, the mice were euthanized, and the tumors were excised for further analysis. For in vivo kidney cancer Renca model, 5 × 10^6^ control or STAMBPL1 KD Renca cells were subcutaneously injected into the flank of 6‐week‐old BALB/c mice, respectively. When the tumor volume reached 50 mm^3^, tumor‐bearing mice were administered either anti‐PD‐1 (BioXcell, New Hampshire, USA; Clone RMP1‐14) or IgG (BioXcell, New Hampshire, USA; Clone 2A3) at a dose of 200 µg per mouse via intraperitoneal injection on days 0, 3, and 6. After 21 days, the mice were euthanized and their tumors were dissected. For survival studies, mice were observed daily following the initial injections, and the survival duration was documented. Notably, mice should be euthanized under the following conditions: 1) tumor volume exceeding 1500 mm^3^; 2) tumor had ulcers with diameter reached 1 cm; 3) body weight loss >20%; 4) decreasing behavioral conditions.^[^ ^22^, ^75^ ^]^

### Statistical Analysis

The results were reported as mean ± standard deviation (S.D.), and statistically analyzed with SPSS version 26.0. The n in the legends represents the times of independent experiments or the number of biological replicates. An unpaired two‐tailed Student′s t test was performed when comparing two groups with a normal distribution and homogeneity of variance, whereas comparisons among three or more groups were conducted using one‐way analysis of variance (ANOVA) followed by Tukey post hoc test. A paired two‐tailed Student's t‐test was used to assess the difference in STAMBPL1 expression between KIRC and the corresponding adjacent cancer tissues. The correlation was analyzed using a Pearson correlation test. Survival analysis was performed using the Kaplan‐Meier method, and the differences between survival curves were assessed using log‐rank tests. P values less than 0.05 were considered to be statistically significant.

### Ethics Approval and Consent to Participate

All experimental and research protocols in this study received approval from the Ethics Committee of Renmin Hospital, Wuhan University (WDRY2023‐K176; WDRM20221110A). All participants in this study provided written informed consent, and the privacy rights of human subjects were strictly observed in accordance with ethical guidelines. The methods employed in this study adhered to the principles outlined in the 1964 Helsinki Declaration. Additionally, all animal experiments were conducted in strict accordance with the recommendations stated in the Guide for the Care and Use of Laboratory Animals of the National Institutes of Health.

## Conflict of Interest

The authors declare no conflict of interest.

## Author Contributions

S.H., X.Q., S.F., and J.H. contributed equally to this work. S.H., J.H., and X.L. conceived the hypothesis and designed the study. S.H., S.F., and M.H. wrote the first draft of the manuscript. S.H., B.Z., J.L., Y.C., and M.W. performed the experiments. S.H., Z.J., and M.H. collected and analyzed the data. L.W. and Z.C. made critical revisions and provided professional advice about the research. X.L. supervised the project. All authors read and approved the final manuscript.

## Supporting information



Supporting Information

Supporting Table

## Data Availability

The data that support the findings of this study are available from the corresponding author upon reasonable request.
