# Supplementary material for: STAMBPL1/TRIM21 Balances AXL Stability Impacting Mesenchymal Phenotype and Immune Response in KIRC
Source: Adv Sci (Weinh). 2024 Nov 11;12(1):2405083. doi: 10.1002/advs.202405083 (PMC11714167; doi:10.1002/advs.202405083)
Supplement: Supplementary file 1 — Supporting Information [file ADVS-12-2405083-s002.pdf]

ADVANCED SCIENCE
Open Access

# Supporting Information

for *Adv. Sci.*, DOI 10.1002/advs.202405083

STAMBPL1/TRIM21 Balances AXL Stability Impacting Mesenchymal Phenotype and Immune Response in KIRC

*Shiyu Huang, Xuke Qin, Shujie Fu, Juncheng Hu, Zhengyu Jiang, Min Hu, Banghua Zhang, Jiachen Liu, Yujie Chen, Minghui Wang, Xiuheng Liu*, Zhiyuan Chen* and Lei Wang**

## Supporting Information

# STAMBPL1/TRIM21 Balances AXL Stability Impacting Mesenchymal Phenotype and Immune Response in KIRC

Shiyu Huang [1, 2†], Xuke Qin [1, 2†], Shujie Fu [1, 2†], Juncheng Hu [1, 2†], Zhengyu Jiang [1, 2], Min Hu [3], Banghua Zhang[1, 2, 4], Jiachen Liu[1, 2, 5], Yujie Chen[1, 2], Minghui Wang[1, 2], Xiuheng Liu [1, 2*], Zhiyuan Chen [1, 2*], Lei Wang [1, 2*]

[1] Department of Urology, Renmin Hospital of Wuhan University, Wuhan, 430060 Hubei, China

[2] Institute of Urologic Disease, Renmin Hospital of Wuhan University, Wuhan, 430060 Hubei, China

[3] Department of Cardiology, Renmin Hospital of Wuhan University, Wuhan 430060, Hubei, China

[4] Hubei Key Laboratory of Digestive System Disease, Wuhan 430060, China.

[5] Central Laboratory, Renmin Hospital of Wuhan University, 430060, Wuhan, Hubei, China.

**Running title:** STAMBPL1 protects AXL from TRIM21-dependent degradation

†These authors made equal contributions to this work.

*Corresponding author:

**Xiuheng Liu, Zhiyuan Chen** and **Lei Wang**

Tel/Fax: +86 027-88041911

E-mail: drliuxh@hotmail.com (Xiuheng Liu), chenzhiyuan163@163.com (Zhiyuan Chen) and drwanglei@whu.edu.cn (Lei Wang).

**Supplementary Figures 1-32 and Figure Legends**

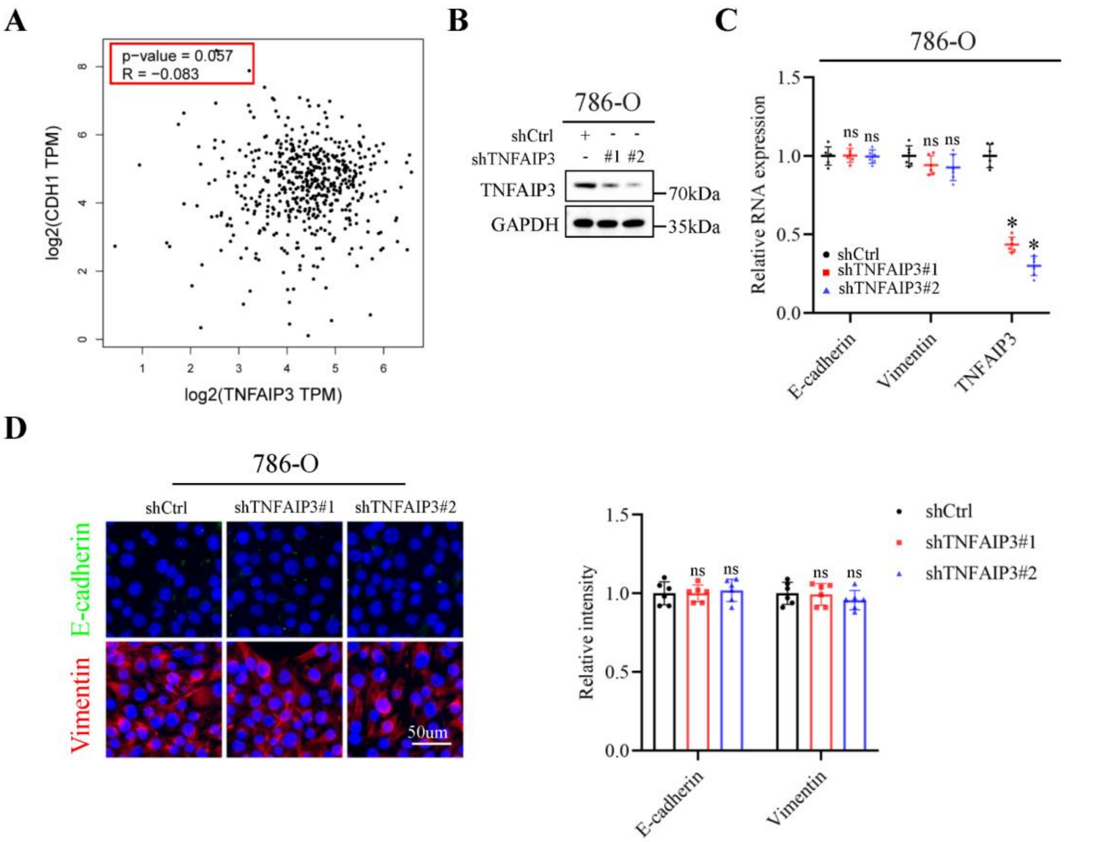


**Figure S1, related to Figure 1.** STAMBPL1 contributes to KIRC cell mesenchymal phenotype and immune evasion. (A) Scatter plot illustrating the correlation between TNFAIP3 and CDH1 in TCGA-KIRC cohort. Pearson's correlation coefficient R with p-value are shown. (B) The efficiency of TNFAIP3 knockdown in 786-O cells (n=3). (C) RT-qPCR analysis of E-cadherin, Vimentin and TNFAIP3 genes in control and TNFAIP3 KD 786-O cells (n=6). (D) Representative IF images and the quantitative results of EMT-related proteins, including E-cadherin and Vimentin, in indicated groups (n=6). All data are represented as mean ± SD, and analyzed using one-way ANOVA followed by Tukey post hoc test.

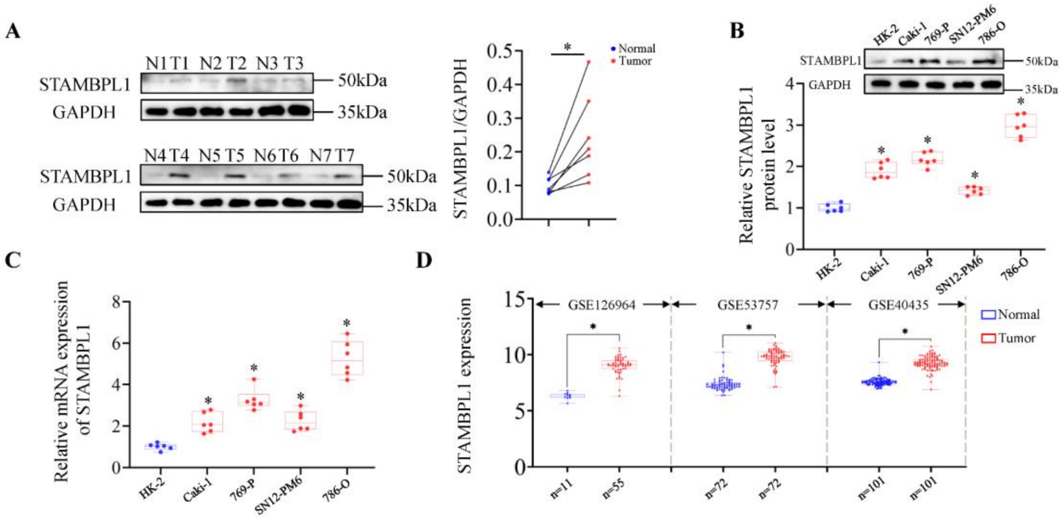


**Figure S2, related to Figure 1.** STAMBPL1 contributes to KIRC cell mesenchymal phenotype and immune evasion. (A) Analysis of STAMBPL1 protein levels in 7 paired KIRC specimens through immunoblotting (n=7). (B, C) Analysis of STAMBPL1 protein and mRNA levels in KIRC cell lines and HK-2 cell line through immunoblotting and RT-qPCR (n=6). (D) Expression level of STAMBPL1 in KIRC specimens from GSE126964 (n=66), GSE53757 (n=144) and GSE40435 (n=202). All data are represented as mean ± SD, and analyzed using one-way ANOVA followed by Tukey post hoc test. For the analysis in (A), a paired two-tailed Student's t-test was conducted. For the analysis in (D), an unpaired two-tailed Student′s t test was performed. *p<0.05.

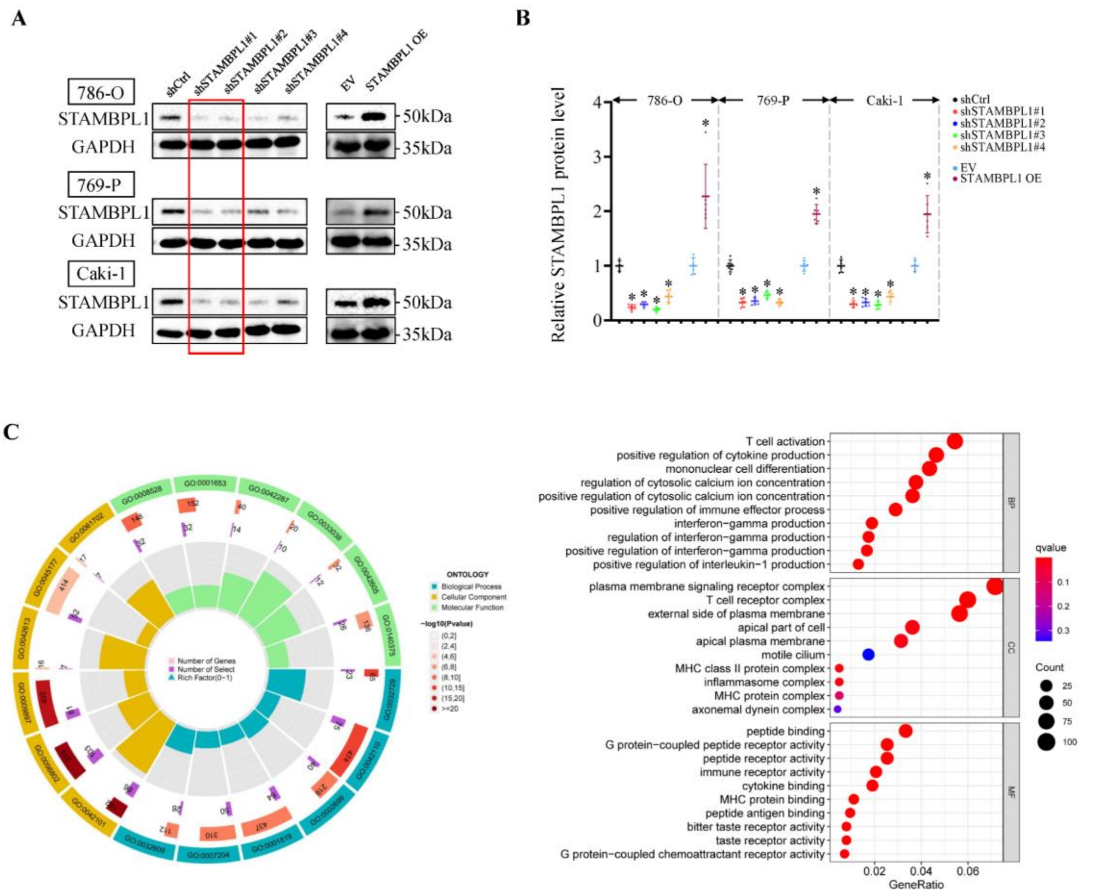


**Figure S3, related to Figure 1.** STAMBPL1 contributes to KIRC cell mesenchymal phenotype and immune evasion. (A, B) The efficiency of STAMBPL1 knockdown or overexpression in 786-O, 769-P cells and Caki-1 cells (n=6). The knockdown efficiency was highest for shSTAMBPL1#1 and shSTAMBPL1#2 (red rectangular box). (C) Gene Ontology (GO) enrichment analysis of co-expressed genes of STAMBPL1 in the TCGA-KIRC cohort was conducted by the R packages clusterProfiler (v4.10.0). All data are represented as mean ± SD, and analyzed using one-way ANOVA followed by Tukey post hoc test. *p<0.05.

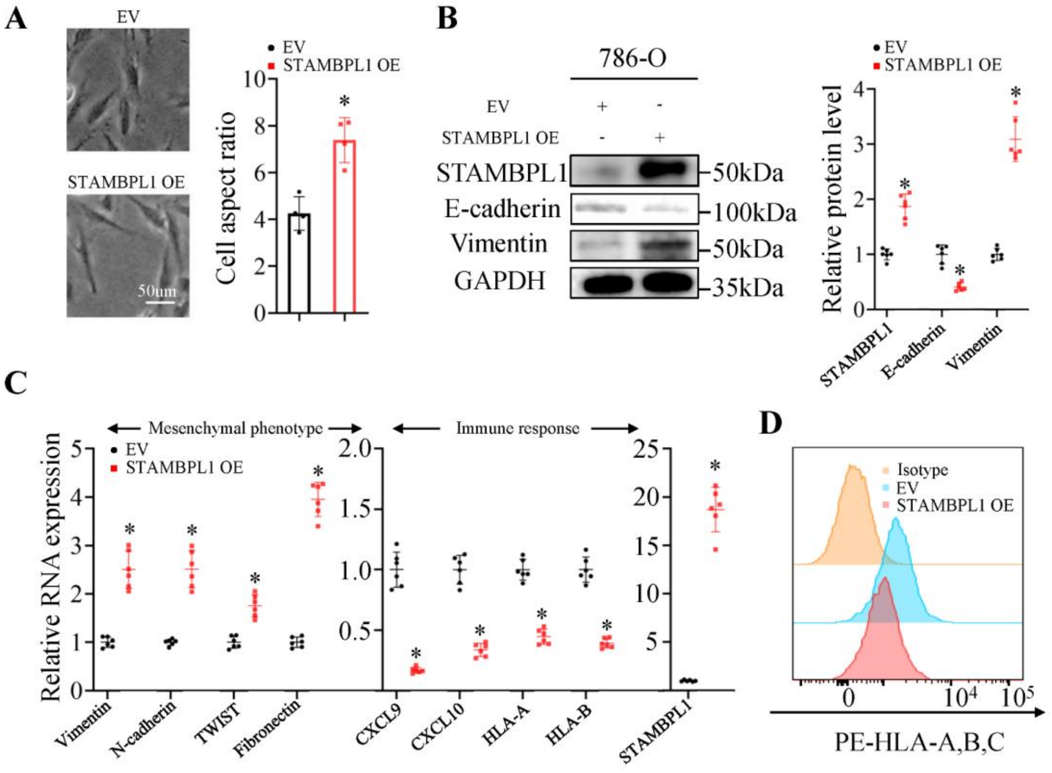


**Figure S4, related to Figure 1.** STAMBPL1 contributes to KIRC cell mesenchymal phenotype and immune evasion. (A) Representative brightfield images of 786-O cells infected with lentiviruses carrying empty vector (EV) or STAMBPL1 (left panel). Cell aspect ratio was quantified (right panel) (n=4). (B) Immunoblots and quantitative results of EMT-related proteins (n=6), GAPDH was used as loading control. (C) RT-qPCR analysis of mesenchymal and immune response genes in 786-O cells infected with lentiviruses carrying EV or STAMBPL1 (n=6). (D) Cell surface HLA-A/B/C expression on 786-O cells infected with the indicated lentiviruses was analyzed by flow cytometry (n=4). All data are represented as mean ± SD, and analyzed using an unpaired two-tailed Student′s t test. *p<0.05; EV, empty vector; OE, overexpressing.

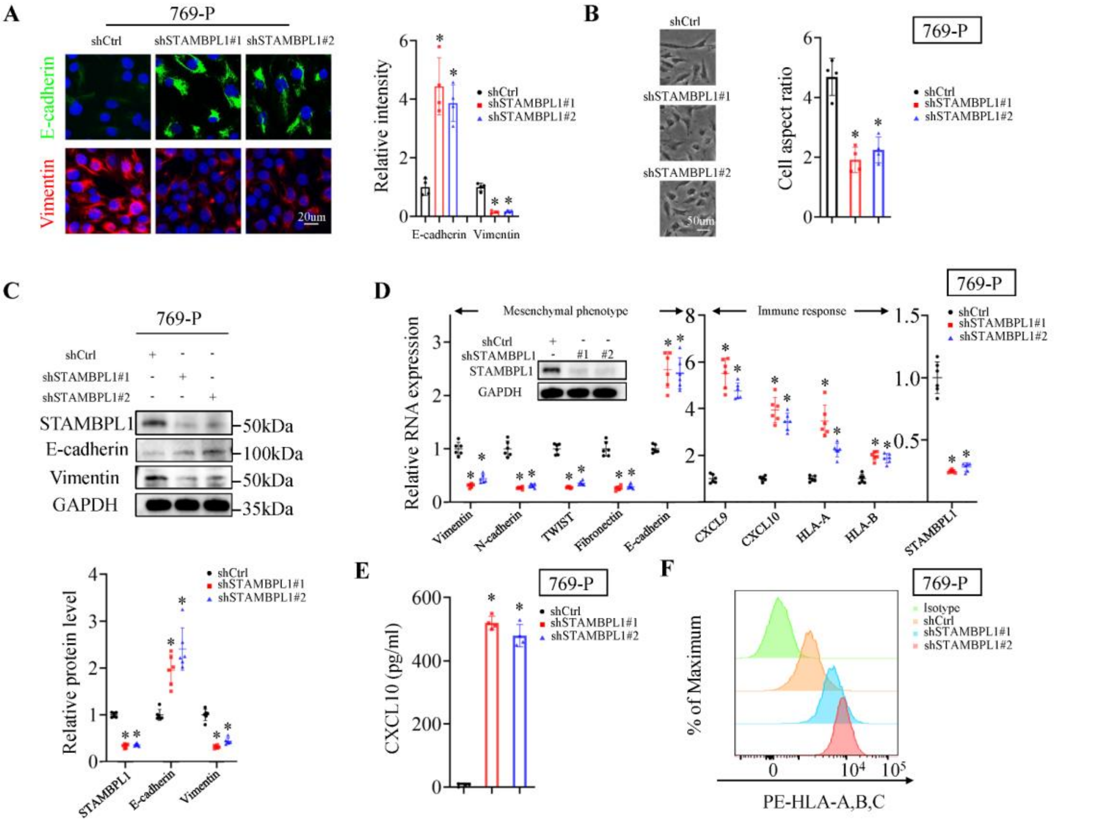


**Figure S5, related to Figure 1.** STAMBPL1 contributes to KIRC cell mesenchymal phenotype and immune evasion. (A) Representative IF images and the quantitative results of EMT-related proteins, including E-cadherin and Vimentin, in indicated groups (n=4). (B) Representative brightfield images of indicated groups (left panel). Cell aspect ratio was quantified (right panel) (n=4). (C) Immunoblots and quantitative results of EMT-related proteins (n=6), GAPDH was used as loading control. (D) RT-qPCR analysis of mesenchymal and immune response genes (n=6). (E) ELISA quantification of CXCL10 secreted from control and STAMBPL1 KD 769-P cells (n = 4). (F) Cell surface HLA-A/B/C expression was analyzed by flow cytometry (n=4). All data are represented as mean ± SD, and analyzed using one-way ANOVA followed by Tukey post hoc test. *$p<0.05$; Ctrl, control.

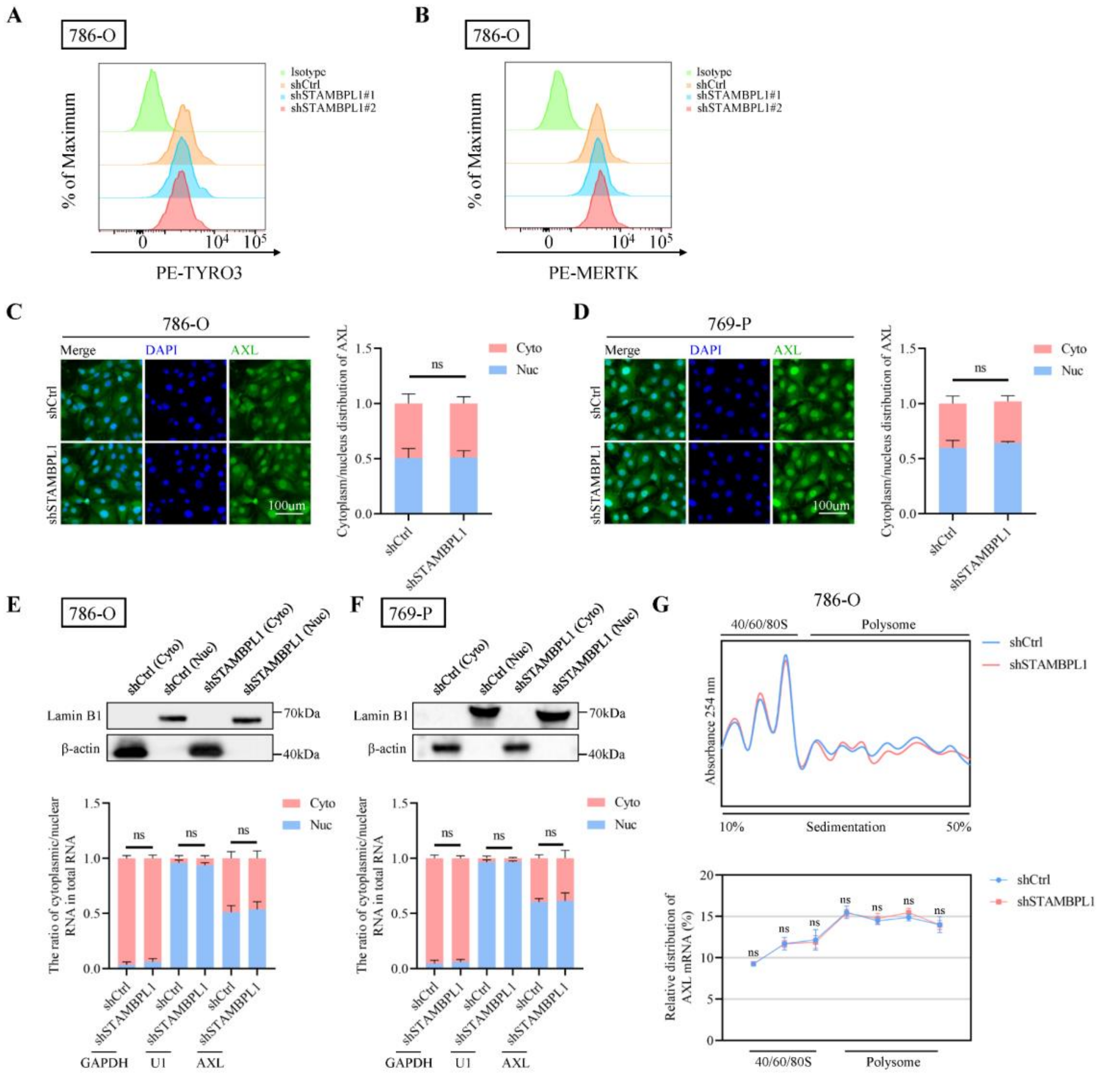


**Figure S6, related to Figure 2.** STAMBPL1 inhibition enhances AXL degradation via the ubiquitin-lysosome pathway. (A, B) Flow cytometry analysis of TYRO3 and MERTK surface expression on control and STAMBPL1 KD 786-O cells (n=4). (C, D) RNA FISH detection using probes targeting AXL in 786-O or 769-P cells infected with the indicated lentiviruses (n=5). (E, F) Cytoplasmic and nuclear fractionation analysis of KIRC cells infected with the indicated lentiviruses. Nuclear and cytosolic extracts were subjected to immunoblotting and RT-qPCR analysis (n=5). GAPDH and U1 served as markers of cytoplasm and nucleus in RT-qPCR, respectively. β-actin and Lamin B1 served as markers of cytoplasm and nucleus in immunoblotting, respectively. (G) The representation of polysome profiling of control and STAMBPL1 KD 786-O

cells, and relative levels of AXL mRNA in each ribosome fraction were quantified (bottom panel) (n=3). All data are represented as mean ± SD, and analyzed using an unpaired two-tailed Student's t test. *p<0.05; ns, not significant; Ctrl, control; Cyto, cytoplasm; Nuc, nucleus.

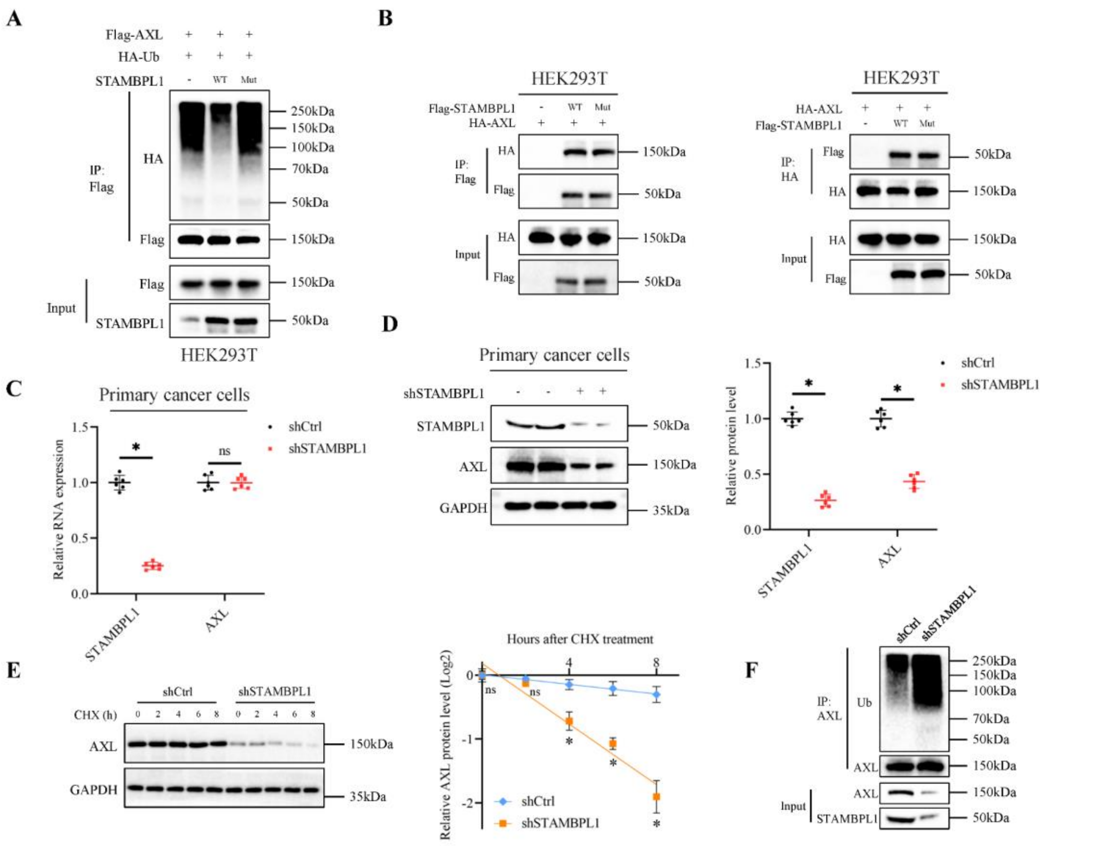


**Figure S7, related to Figure 2.** STAMBPL1 inhibition enhances AXL degradation via the ubiquitin-lysosome pathway. (A) Immunoblotting analysis of WCL and anti-Flag immunoprecipitates (IPs) derived from lysates of HEK293T cells transfected with the indicated constructs (n=3). (B) Co-IP assays of the interaction between STAMBPL1 and AXL in HEK293T cells transfected with the indicated plasmids (n=3). (C) RT-qPCR analysis of STAMBPL1 and AXL genes in control and shSTAMBPL1 treated primary cancer cells (n=6). (D) Immunoblots and quantitative results of STAMBPL1 and AXL proteins (n=6). (E) Immunoblotting analysis of WCL

derived from control and STAMBPL1 KD primary cancer cells treated with 100 µg/mL CHX at indicated time points (left panel). AXL band intensity was normalized to GAPDH and then to the t = 0 time point (right panel) (n=3). (F) Immunoblotting analysis of WCL and anti-AXL IPs derived from lysates of control and shSTAMBPL1 treated primary cancer cells (n=3). All data are represented as mean ± SD, and analyzed using an unpaired two-tailed Student's t test. *p<0.05; ns, not significant; Ctrl, control; CHX, cycloheximide.

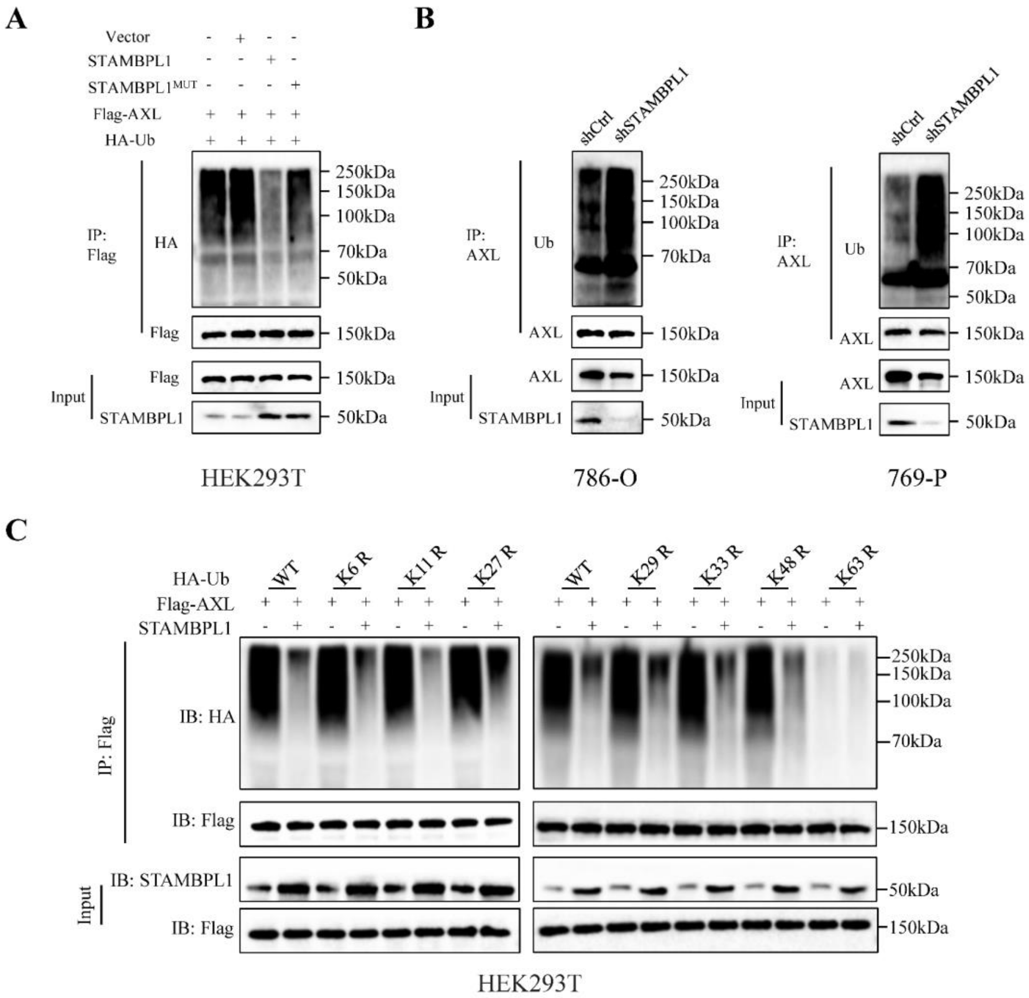


**Figure S8, related to Figure 2.** STAMBPL1 inhibition enhances AXL degradation via the ubiquitin-lysosome pathway. (A) Immunoblotting analysis of WCL and anti-Flag IPs derived from lysates of HEK293T cells transfected with the indicated constructs (n=3). (B) Immunoblotting

analysis of WCL and anti-AXL IPs derived from lysates of control and shSTAMBPL1 KIRC cell lines (n=3). (C) HEK293T cells were transfected with the indicated plasmids, and the ubiquitination of AXL was detected by Co-IP and immunoblotting (n=3). Ctrl, control; WT, wild type; Mut, mutant.

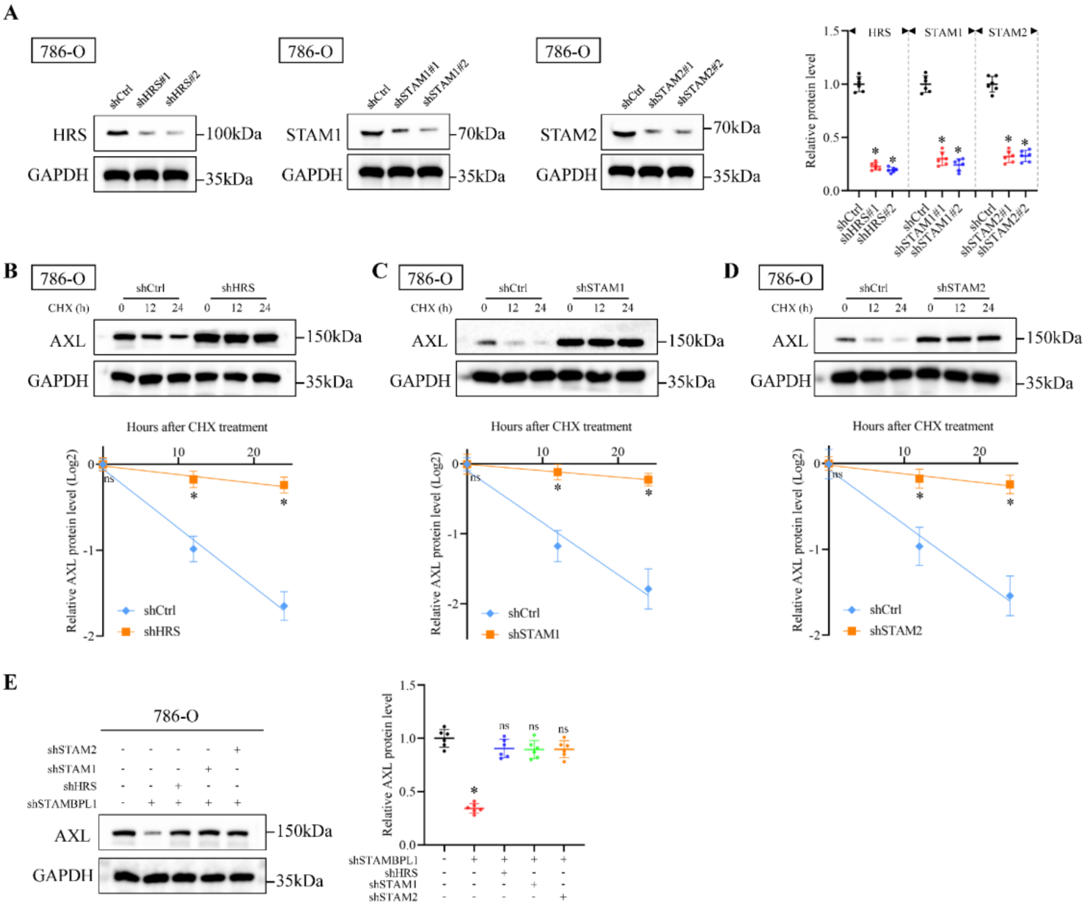


**Figure S9, related to Figure 2.** STAMBPL1 inhibition enhances AXL degradation via the ubiquitin-lysosome pathway. (A) The efficiency of HRS, STAM1 and STAM2 knockdown in 786-O cells (n=6). (B-D) Immunoblotting analysis of WCL derived from 786-O cells infected with the indicated lentiviruses (top panel). Cells were treated with 100 μg/mL CHX at indicated time points. AXL band intensity was normalized to GAPDH and then to the t = 0 time point (bottom panel) (n=3). (E) Immunoblotting analysis of WCL derived from 786-O cells infected with the indicated lentiviruses (left panel). Quantitative results (right panel) (n=6). All data are represented as mean ±

SD, and analyzed using an unpaired two-tailed Student′s t test. For the analysis in (A, E), one-way ANOVA followed by Tukey post hoc test was performed. *p<0.05; ns, not significant; Ctrl, control; CHX, cycloheximide.

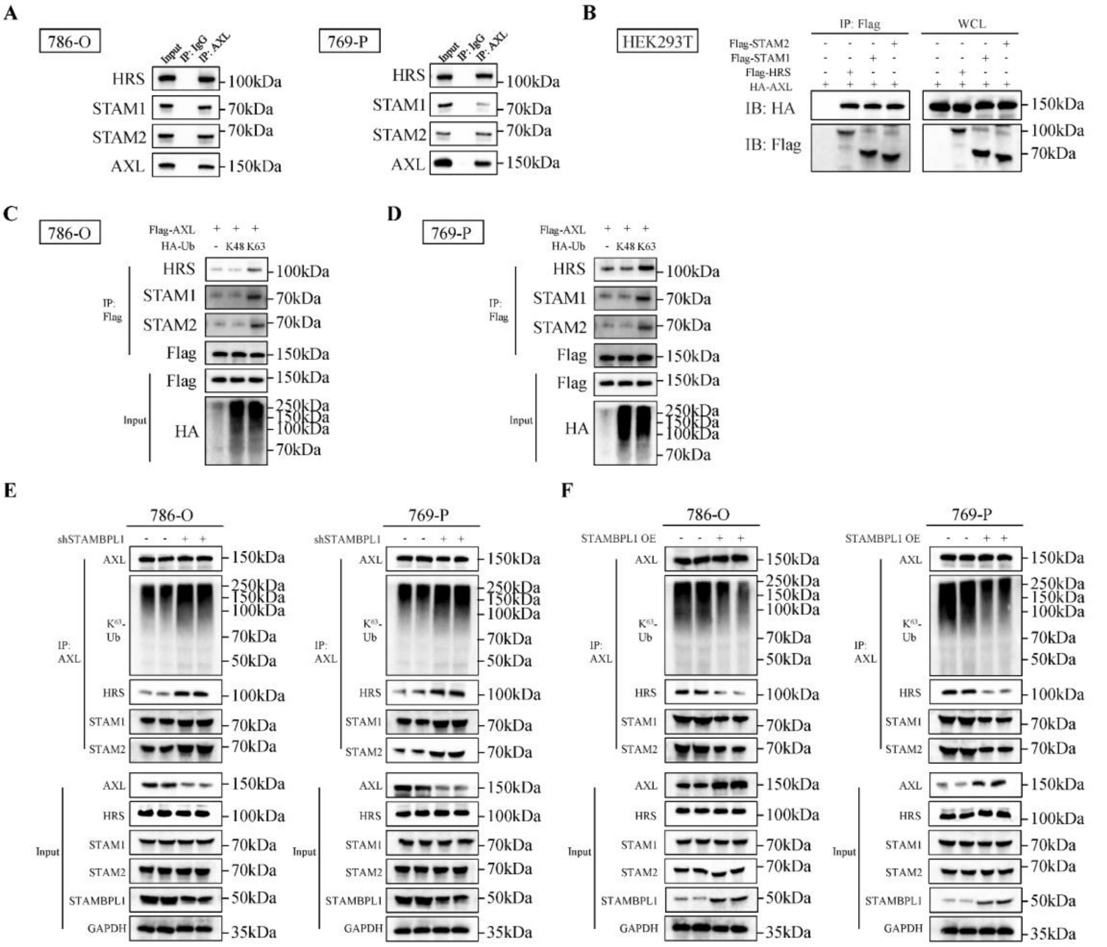


**Figure S10, related to Figure 2.** STAMBPL1 inhibition enhances AXL degradation via the ubiquitin-lysosome pathway. (A) Endogenous Co-IP analysis of the interaction of AXL and ESCRT complexes (including HRS, STAM1 and STAM2) in KIRC cell lines using anti-IgG or anti-AXL antibodies (n=3). (B) Immunoblotting analysis of WCL and anti-Flag IPs derived from lysates of HEK293T cells transfected with the indicated constructs (n=3). (C, D) KIRC cells were transfected with the indicated plasmids, followed by Co-IP and immunoblotting (n=3). (E) Immunoblotting

analysis of WCL and anti-AXL IPs derived from lysates of shCtrl- or shSTAMBPL1-treated KIRC cells (n=3). (F) Immunoblotting analysis of WCL and anti-AXL IPs derived from lysates of KIRC cells infected with lentiviruses carrying EV or STAMBPL1 (n=3). Ctrl, control; OE, overexpressing.

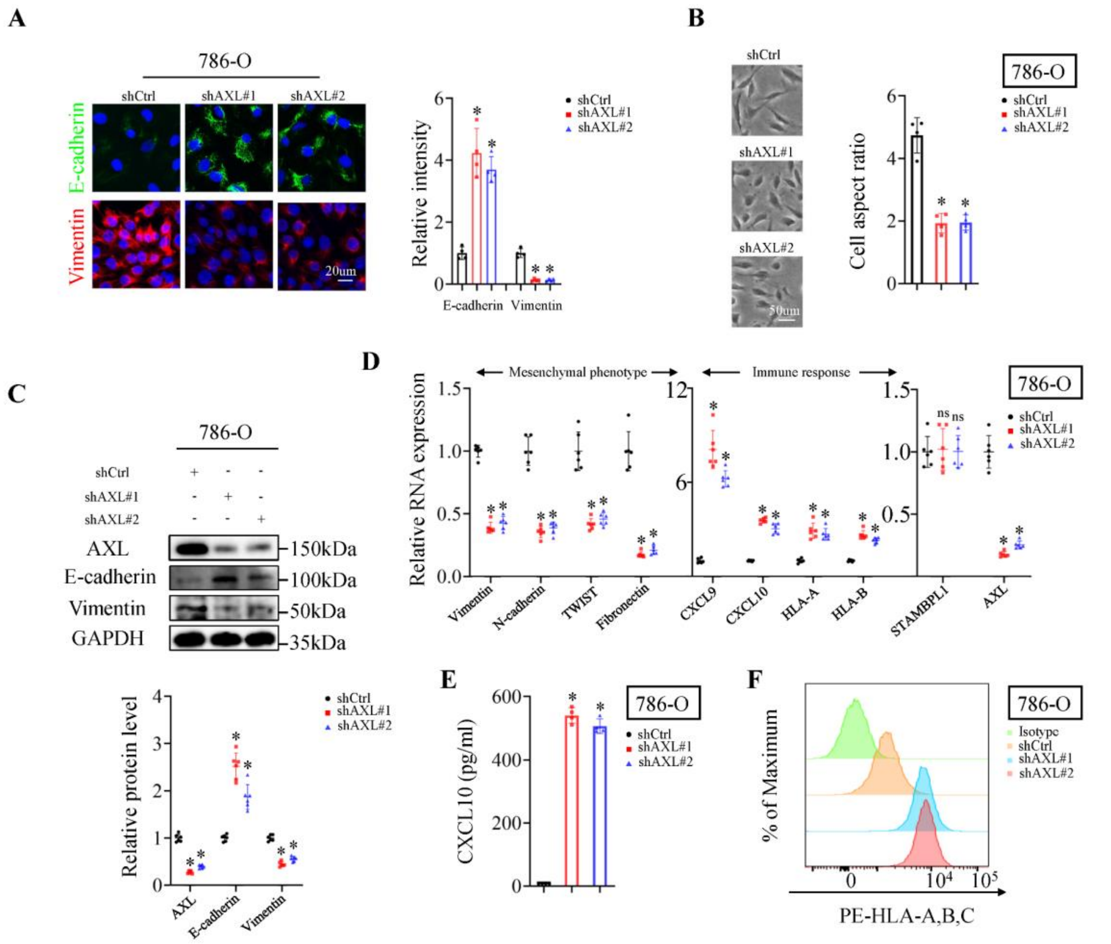


**Figure S11, related to Figure 3.** STAMBPL1 specifically interacts with AXL and STAMBPL1 KD effects are largely rescued by AXL expression. (A) Representative IF images and the quantitative results of EMT-related proteins, including E-cadherin and Vimentin, in indicated groups (n=4). (B) Representative brightfield images of indicated groups (left panel). Cell aspect ratio was quantified (right panel) (n=4). (C) Immunoblots and quantitative results of EMT-related proteins (n=6). (D) RT-qPCR analysis of mesenchymal and immune response genes (n=6). (E) ELISA quantification of CXCL10 secreted from control and AXL KD 786-O cells (n=4). (F) Cell surface HLA-A/B/C

expression was analyzed by flow cytometry (n=4). All data are represented as mean ± SD, and analyzed using one-way ANOVA followed by Tukey post hoc test. *p<0.05; Ctrl, control.

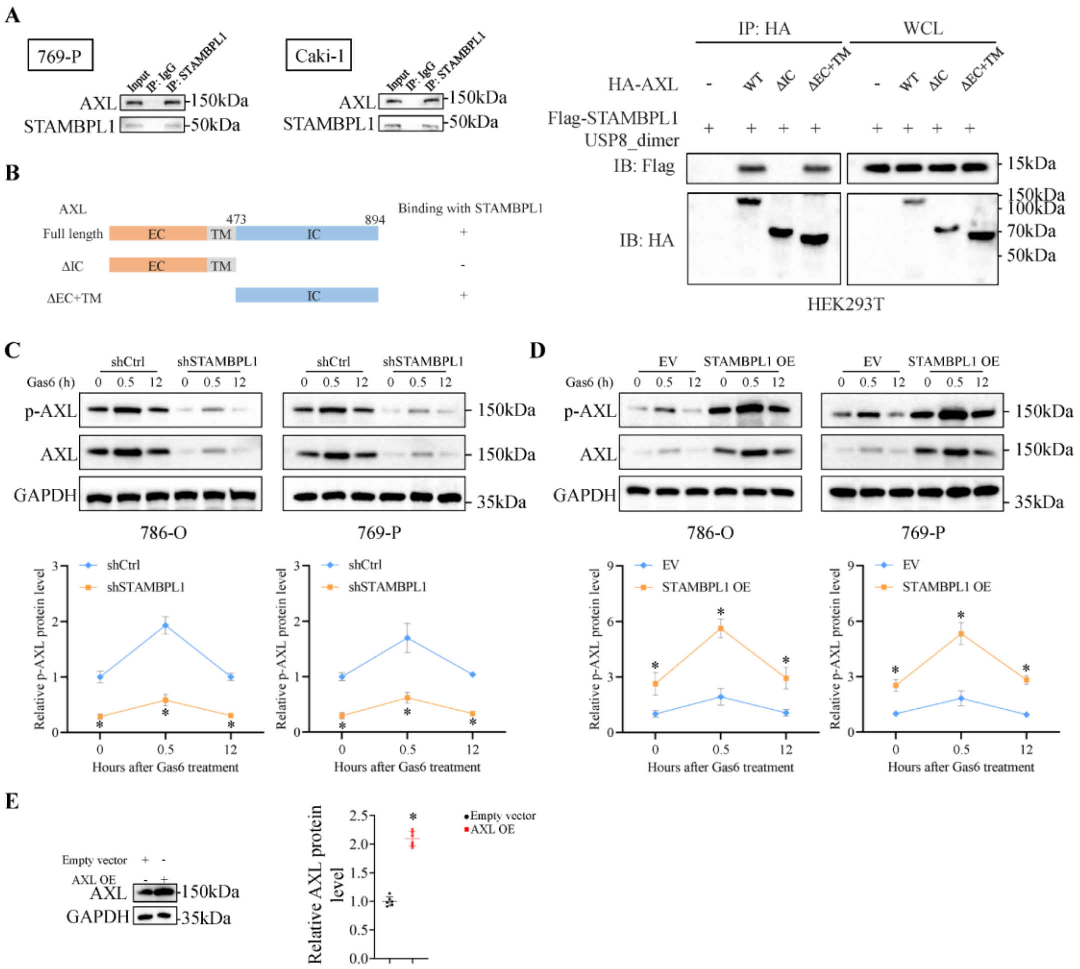


**Figure S12, related to Figure 3.** STAMBPL1 specifically interacts with AXL and STAMBPL1 KD effects are largely rescued by AXL expression. (A) Co-IP analysis of the endogenous STAMBPL1/AXL proteins interaction in the 769-P cells and Caki-1 cells (n=3). IgG, IP control. (B) Schematic representation of full length AXL and its deletion mutants (top panel). HEK293T cells were co-transfected with Flag-STAMBPL1-USP8_dimer and HA-tagged full length AXL or its deletion mutants, and cell lysates were analyzed by IP with HA antibody and protein A/G beads followed by immunoblotting analysis with antibodies against Flag and HA (bottom panel) (n=3).

(C, D) Immunoblotting analysis of WCL derived from KIRC cells infected with the indicated lentiviruses (top panel). Cells were treated with human recombinant Gas6 (500 ng/mL) for the indicated times. p-AXL band intensity was normalized to GAPDH and then to the t = 0 time point (bottom panel) (n=3). (E) The efficiency of AXL overexpression in 786-O cells (n=6). All data are represented as mean ± SD, and analyzed using an unpaired two-tailed Student′s t test. *p<0.05; WT, wild type; WCL, whole-cell lysates; Ctrl, control; OE, overexpressing.

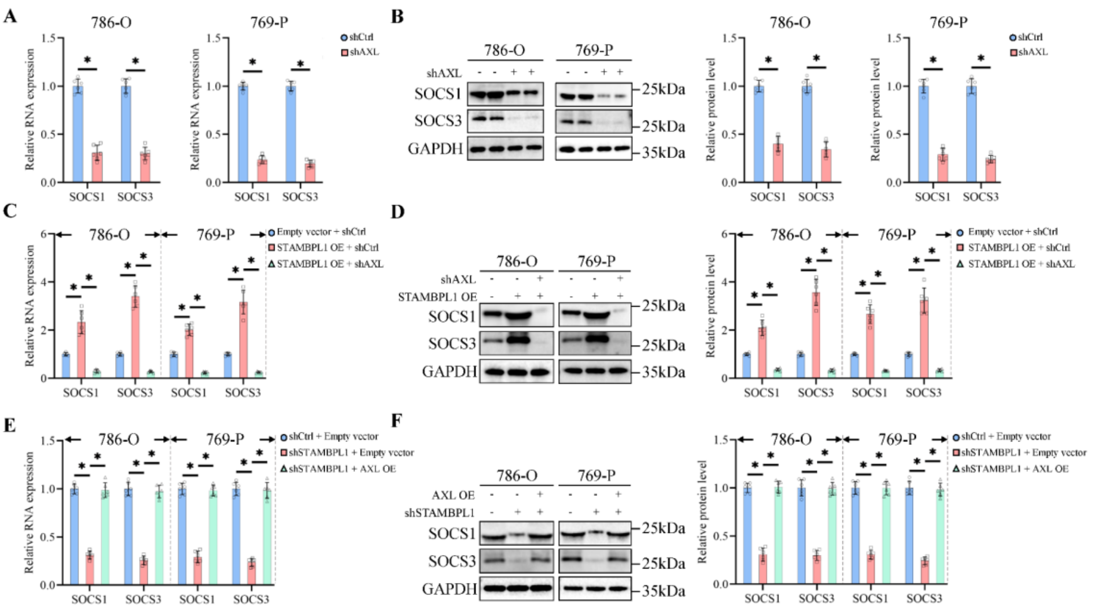


**Figure S13, related to Figure 3.** STAMBPL1 specifically interacts with AXL and STAMBPL1 KD effects are largely rescued by AXL expression. (A) RT-qPCR analysis of SOCS1 and SOCS3 expression in control and AXL KD KIRC cells (n=6). (B) Immunoblotting analysis of WCL derived from control and AXL KD KIRC cells (left panel). Quantitative results (right panel) (n=6). (C, E) RT-qPCR analysis of SOCS1 and SOCS3 expression in KIRC cells infected with the indicated lentiviral particles (n=6). (D, F) Immunoblots and quantitative results of indicated proteins (n=6), GAPDH was used as loading control. All data are represented as mean ± SD, and analyzed using

one-way ANOVA followed by Tukey post hoc test. For the analysis in (A, B), an unpaired two-tailed Student's t test was performed. *p<0.05; Ctrl, control; OE, overexpressing.

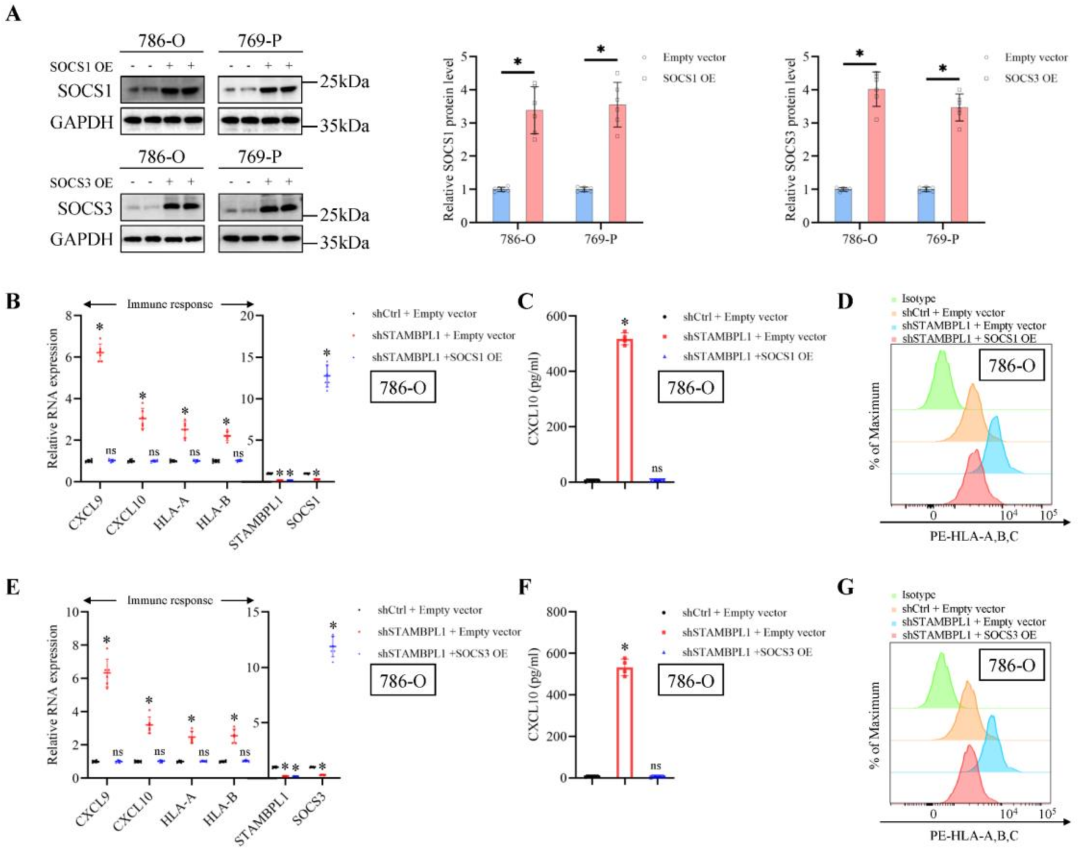


**Figure S14, related to Figure 3.** STAMBPL1 specifically interacts with AXL and STAMBPL1 KD effects are largely rescued by AXL expression. (A) Immunoblotting analysis was used to determine the overexpression efficiency of SOCS1 and SOCS3 in KIRC cells (n=6). (B, E) RT-qPCR analysis of immune response genes in 786-O cells infected with the indicated lentiviral particles (n=6). (C, F) ELISA quantification of CXCL10 secreted from control and STAMBPL1 KD 786-O cells transfected with indicated constructs (n=4). (D, G) Cell surface HLA-A/B/C expression was analyzed by flow cytometry (n=4). All data are represented as mean ± SD, and analyzed using one-way ANOVA followed by Tukey post hoc test. For the analysis in (A), an unpaired two-tailed Student's t test was performed. *p<0.05; ns, not significant; Ctrl, control; OE, overexpressing.

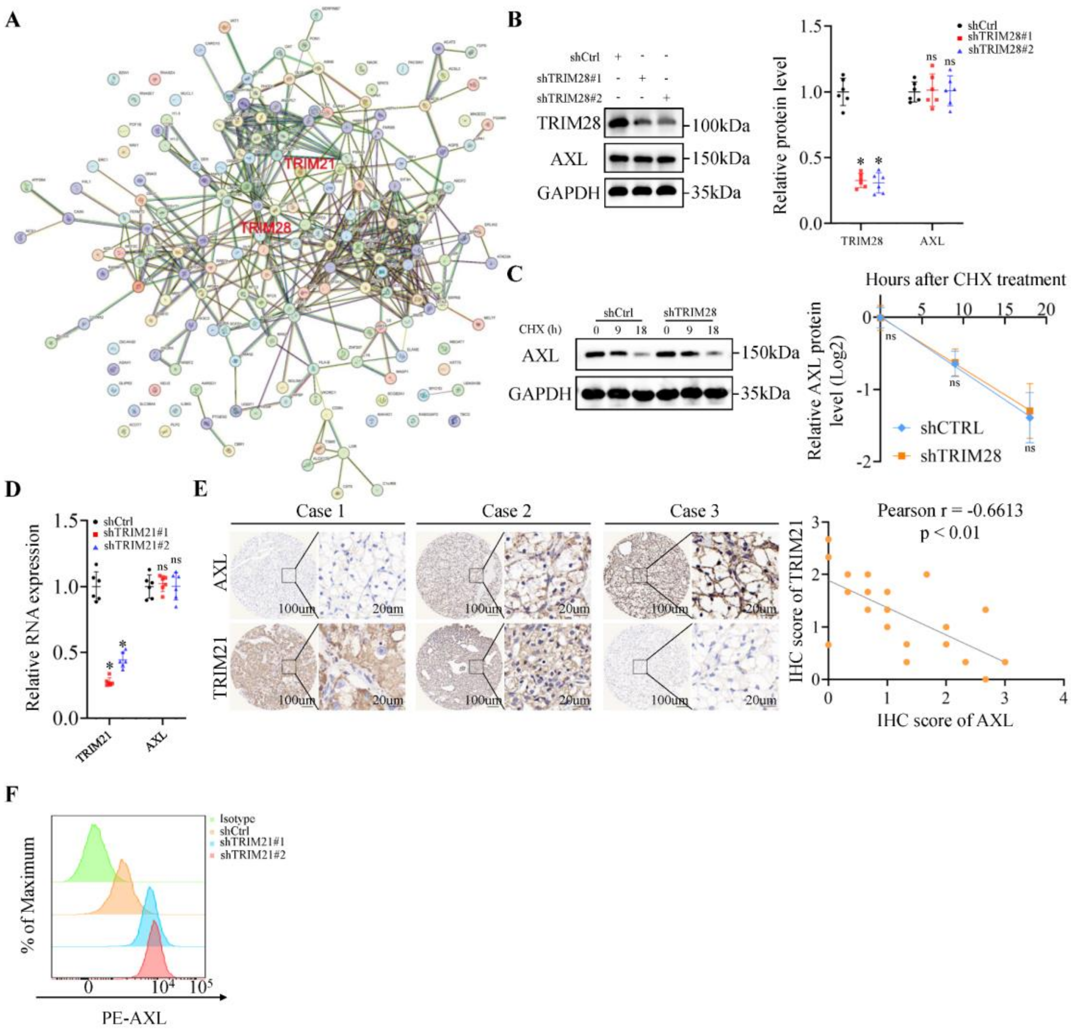


**Figure S15, related to Figure 4.** STAMBPL1 and E3 ligase TRIM21 balance the level of AXL K63-linked ubiquitination. (A) Using the STRING program to analyze the proteins potentially interacting with STAMBPL1 obtained from mass spectrometry. (B) Immunoblotting analysis of WCL derived from control and TRIM28 KD 786-O cells (n=6). (C) Immunoblotting analysis of WCL derived from control and TRIM28 KD 786-O cells treated with 100 μg/mL CHX at indicated time points (left panel). AXL band intensity was normalized to GAPDH and then to the t = 0 time point (right panel) (n=6). (D) RT-qPCR analysis of AXL gene in control and TRIM21 KD 786-O cells (n=6). (E) Tumor tissues of KIRC samples were subjected to IHC staining for AXL and

TRIM21 (left panel) (n=20). Scatter plot illustrating the correlation of the IHC scores corresponding to AXL and TRIM21 (right panel). Pearson’s correlation coefficient r with P-value are shown. (F) Flow cytometry analysis of AXL surface expression on control and TRIM21 KD 786-O cells (n=4). All data are represented as mean ± SD, and analyzed using one-way ANOVA followed by Tukey post hoc test. For the analysis in (C), an unpaired two-tailed Student′s t test was performed. For the analysis in (E), Pearson correlation test was conducted. *p<0.05; ns, not significant; Ctrl, control; CHX, cycloheximide.

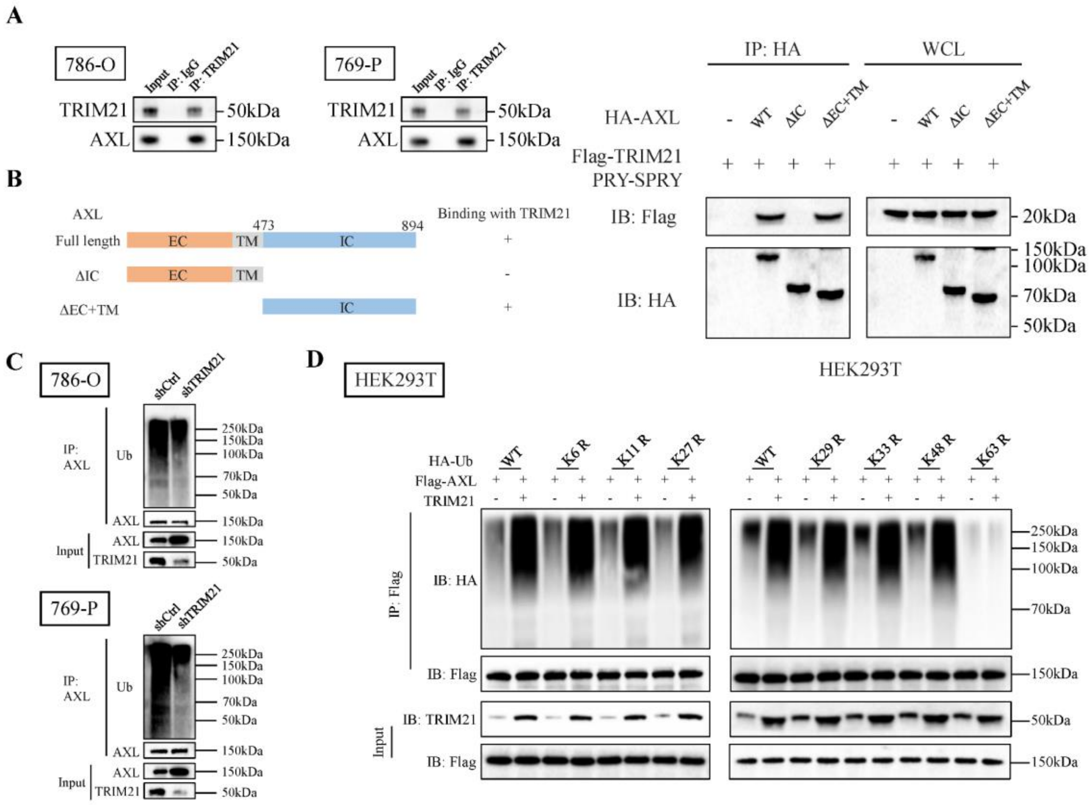


**Figure S16, related to Figure 4.** STAMBPL1 and E3 ligase TRIM21 balance the level of AXL K63-linked ubiquitination. (A) Co-IP analysis of the endogenous TRIM21/AXL proteins interaction in the 786-O cells and 769-P cells. IgG, IP control (n=3). (B) Schematic representation of full length AXL and its deletion mutants (left panel). HEK293T cells were co-transfected with Flag-TRIM21-

PRY-SPRY and HA-tagged full length AXL or its deletion mutants, and cell lysates were analyzed by IP with HA antibody and protein A/G beads followed by immunoblotting analysis with antibodies against Flag and HA (right panel) (n=3). (C) Immunoblotting analysis of WCL and anti-AXL IPs derived from lysates of control and TRIM21 KD KIRC cells (n=3). (D) HEK293T cells were transfected with the indicated plasmids, and the ubiquitination of AXL was detected by Co-IP and immunoblotting (n=3). WT, wild type.

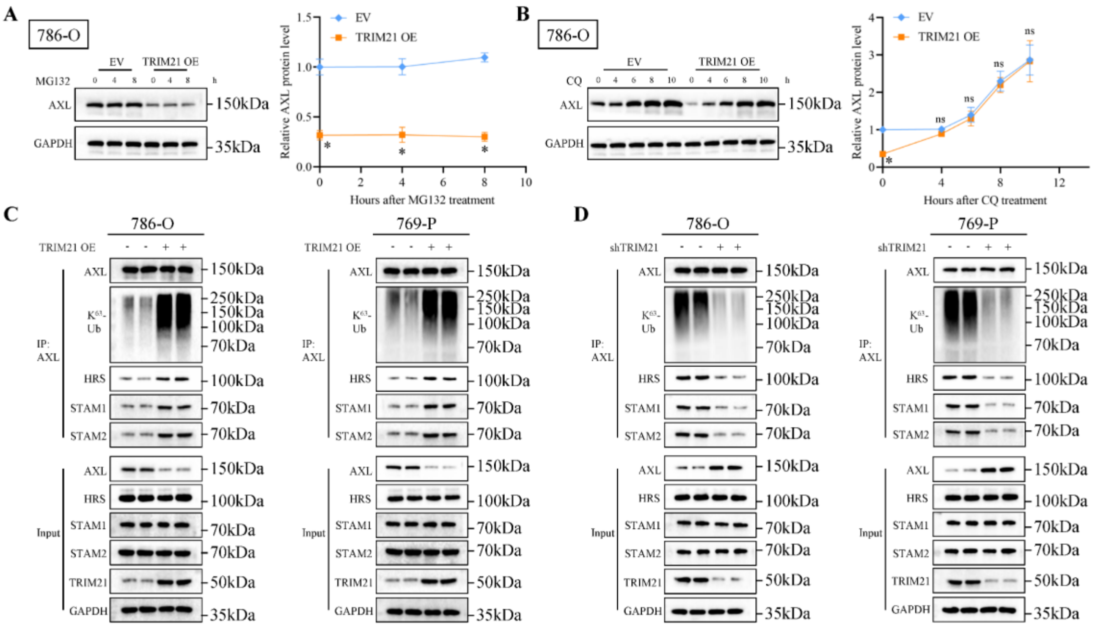


**Figure S17, related to Figure 4.** STAMBPL1 and E3 ligase TRIM21 balance the level of AXL K63-linked ubiquitination. (A, B) Control and TRIM21 OE 786-O cells were treated with 10 μM MG132 or 50 μM CQ for the indicated hours, the WCL were immunoblotted for AXL or GAPDH (left panel). AXL band intensity was normalized to GAPDH and then to the t = 0 time point (right panel) (n=3). (C) Immunoblotting analysis of WCL and anti-AXL IPs derived from lysates of KIRC cells infected with lentiviruses carrying EV or TRIM21 (n=3). (D) Immunoblotting analysis of WCL and anti-AXL IPs derived from lysates of shCtrl- or shTRIM21-treated KIRC cells (n=3). All data

are represented as mean ± SD, and analyzed using an unpaired two-tailed Student's t test. *p<0.05; ns, not significant; EV, empty vector; OE, overexpressing; CQ, chloroquine.

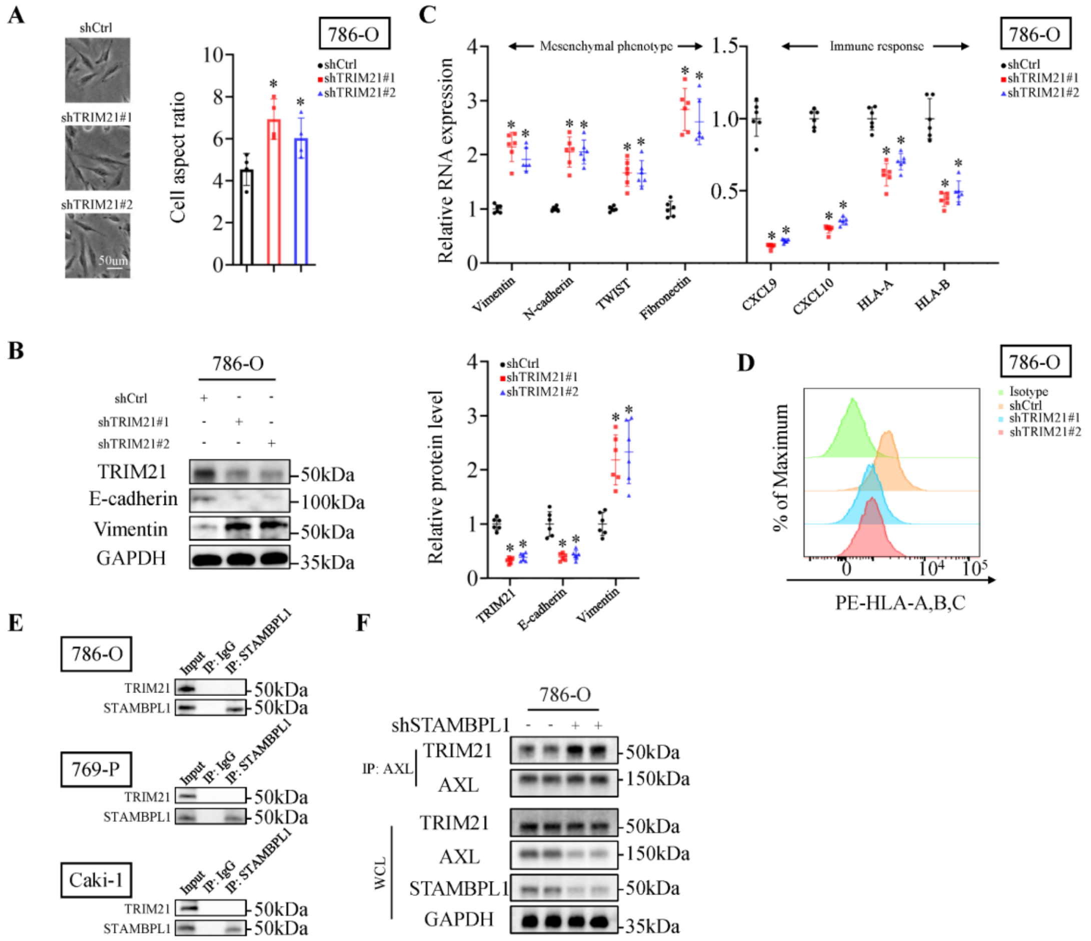


**Figure S18, related to Figure 4.** STAMBPL1 and E3 ligase TRIM21 balance the level of AXL K63-linked ubiquitination. (A) Representative brightfield images of indicated groups (left panel). Cell aspect ratio was quantified (right panel) (n=4). (B) Immunoblots and quantitative results of EMT-related proteins (n=6). (C) RT-qPCR analysis of mesenchymal and immune response genes (n=6). (D) Cell surface HLA-A/B/C expression was analyzed by flow cytometry (n=4). (E) Co-IP analysis of the endogenous STAMBPL1/TRIM21 proteins interaction in the KIRC cell lines. IgG, IP control (n=3). (F) Co-IP of TRIM21 with endogenous AXL in 786-O cells infected with

lentiviruses carrying indicated shRNAs (n=3). All data are represented as mean ± SD, and analyzed using one-way ANOVA followed by Tukey post hoc test. *p<0.05; Ctrl, control; WCL, whole-cell lysates.

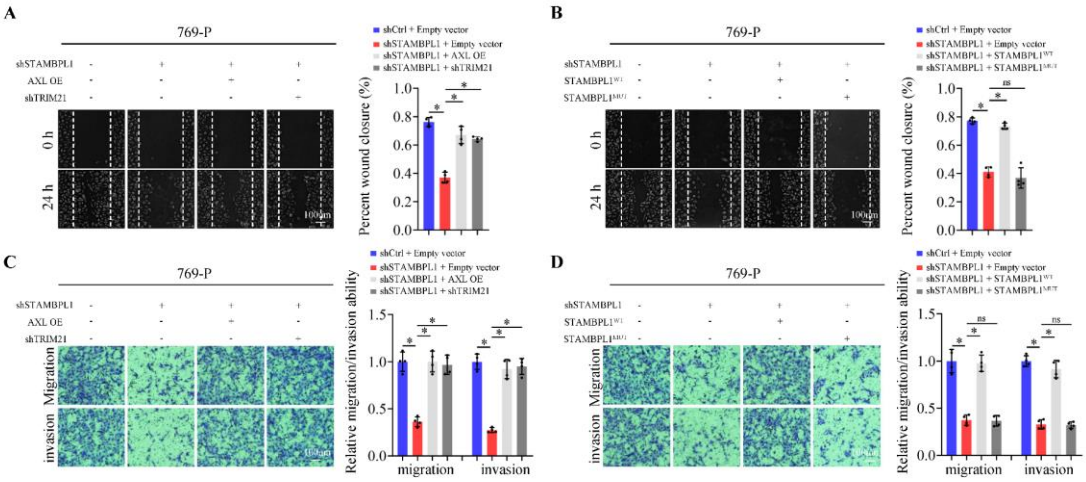


**Figure S19, related to Figure 5.** Targeting STAMBPL1 suppresses metastasis of KIRC cells. (A, B) Representative images and the quantitative results of wound healing assay (n=4). (C, D) Representative images and the quantitative results of transwell assay (n=4). All data are represented as mean ± SD, and analyzed using one-way ANOVA followed by Tukey post hoc test. *p<0.05; ns, not significant; WT, wild type; Mut, mutant; OE, overexpressing.

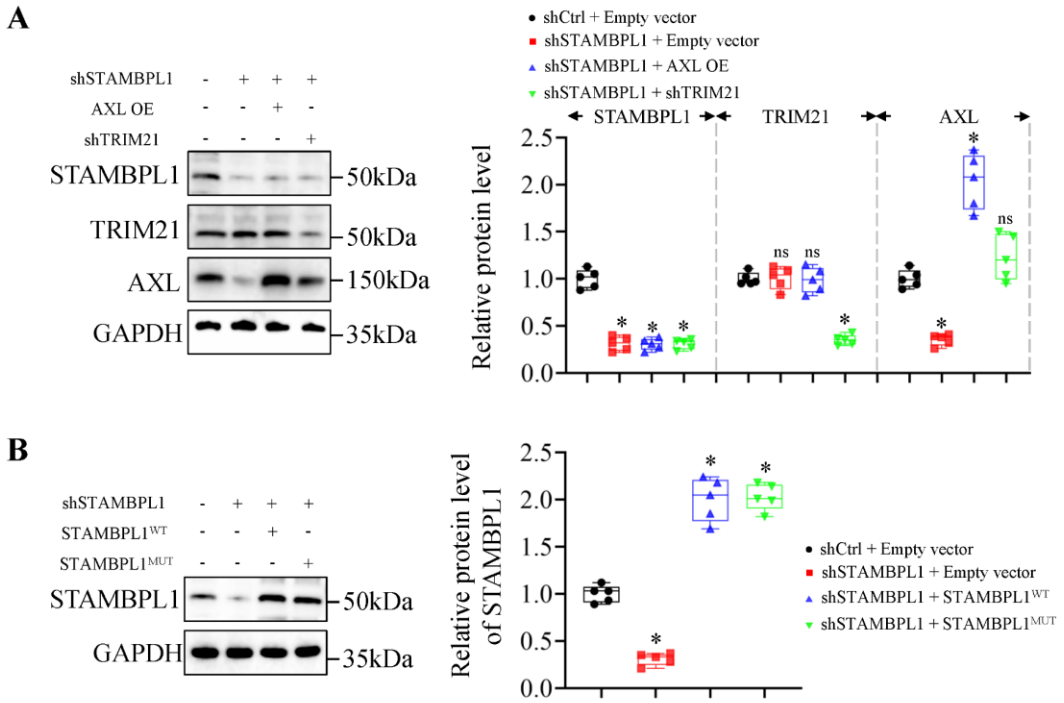


**Figure S20, related to Figure 5.** Targeting STAMBPL1 suppresses metastasis of KIRC cells. (A, B）Immunoblotting analysis of indicated proteins in excised orthotopic tumor (n=5). All data are represented as mean ± SD, and analyzed using one-way ANOVA followed by Tukey post hoc test. *$p<0.05$; ns, not significant; WT, wild type; Mut, mutant; Ctrl, control; OE, overexpressing.

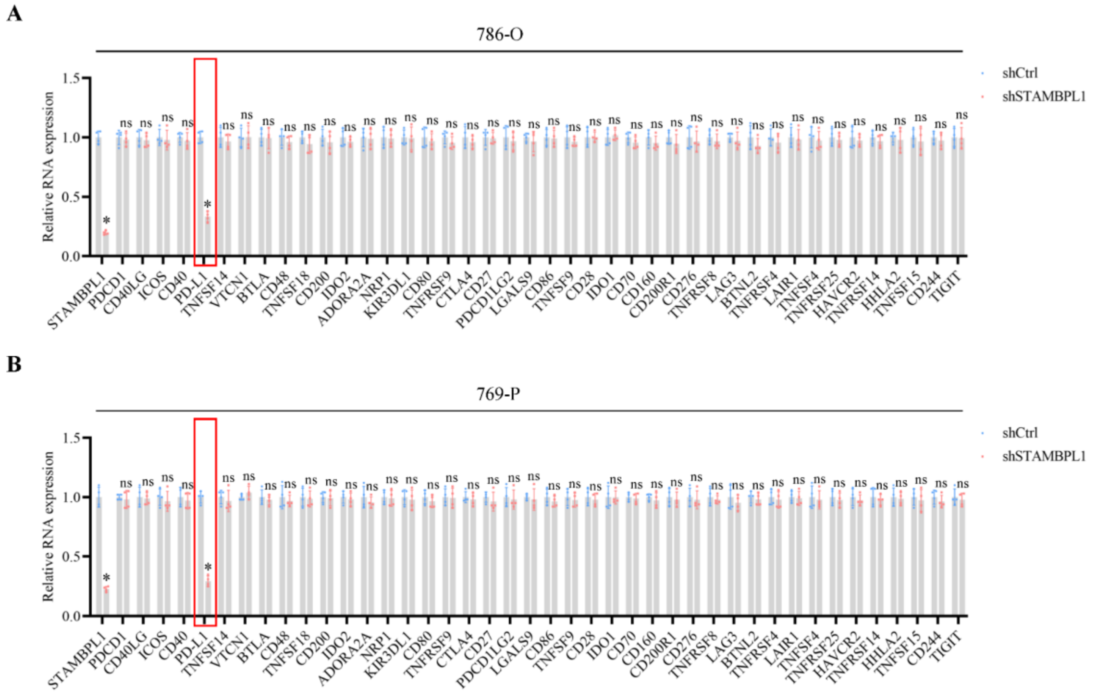


**Figure S21, related to Figure 6.** STAMBPL1 increases PD-L1 levels via stimulating p65 nuclear translocation and silencing STAMBPL1 potentiates the antitumor efficacy of PD-1 blockade. (A, B) mRNA levels of indicated immune checkpoint molecules from control and STAMBPL1 KD KIRC cells were analyzed using RT-qPCR analysis (n=4). All data are represented as mean ± SD, and analyzed using an unpaired two-tailed Student′s t test. *$p<0.05$; ns, not significant; Ctrl, control.

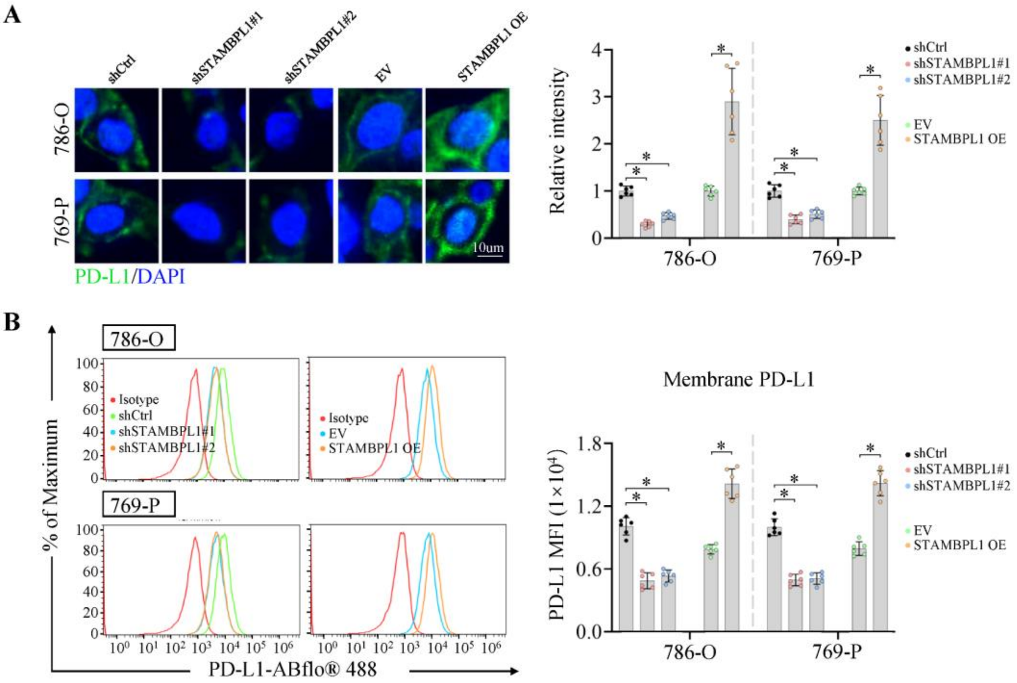


**Figure S22, related to Figure 6.** STAMBPL1 increases PD-L1 levels via stimulating p65 nuclear translocation and silencing STAMBPL1 potentiates the antitumor efficacy of PD-1 blockade. (A) IF staining was used to analyze the effect of STAMBPL1 on PD-L1 protein levels (n=6). (B) Cell surface PD-L1 expression was analyzed by flow cytometry (n=6). All data are represented as mean ± SD, and analyzed using one-way ANOVA followed by Tukey post hoc test. *$p<0.05$; Ctrl, control; EV, empty, vector, OE, overexpressing.

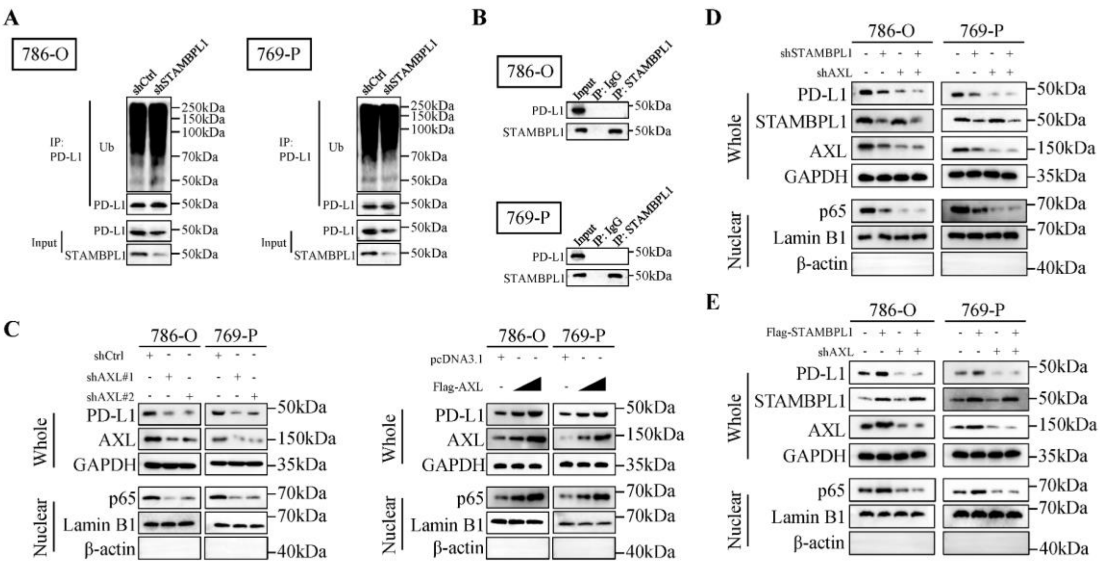


**Figure S23, related to Figure 6.** STAMBPL1 increases PD-L1 levels via stimulating p65 nuclear translocation and silencing STAMBPL1 potentiates the antitumor efficacy of PD-1 blockade. (A) Immunoblotting analysis of WCL and anti-PD-L1 IPs derived from lysates of control and STAMBPL1 KD KIRC cells (n=3). (B) Co-IP analysis of the endogenous STAMBPL1/PD-L1 proteins interaction in the 786-O cells and 769-P cells. IgG, IP control (n=3). (C-E) Immunoblotting analysis of WCL and nuclear lysates derived from KIRC cells infected with the indicated lentiviruses or/and transfected with the specified plasmids (n=3). Ctrl, control.

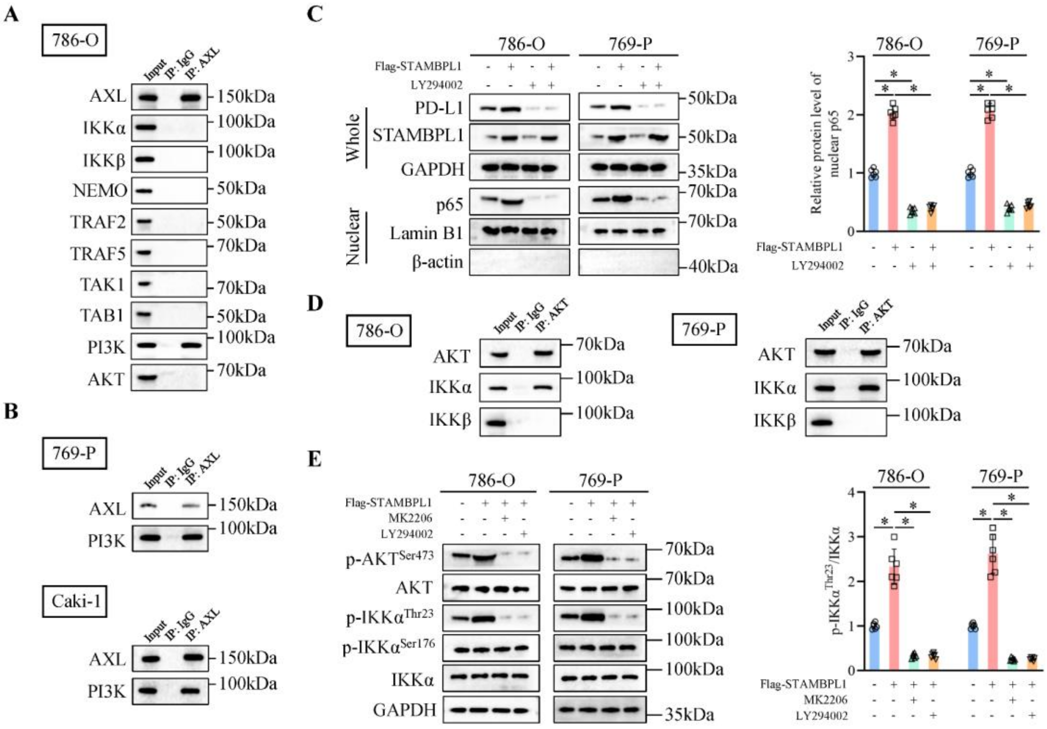


**Figure S24, related to Figure 6.** STAMBPL1 increases PD-L1 levels via stimulating p65 nuclear translocation and silencing STAMBPL1 potentiates the antitumor efficacy of PD-1 blockade. (A) Co-IP of endogenous AXL with NF-κB signaling proteins. IgG, IP control (n=3). (B) Co-IP analysis of the endogenous AXL/PI3K proteins interaction in the 769-P cells and Caki-1 cells. IgG, IP control (n=3). (C) KIRC cells transfected with indicated constructs were treated with or without LY294002 (20 μM) for 24 hours. Then cells were collected for immunoblotting analysis (n=6). (D) Cell lysates from KIRC cells were analyzed by Co-IP using antibodies against AKT, then subjected to immunoblotting analysis (n=3). (E) 786-O and 769-P cells were transfected with the indicated constructs. After treatment with LY294002 (20 μM) or MK2206 (5 μM) for 24 hours, cells were harvested for immunoblotting analysis (n=6). All data are represented as mean ± SD, and analyzed using one-way ANOVA followed by Tukey post hoc test. *$p<0.05$.

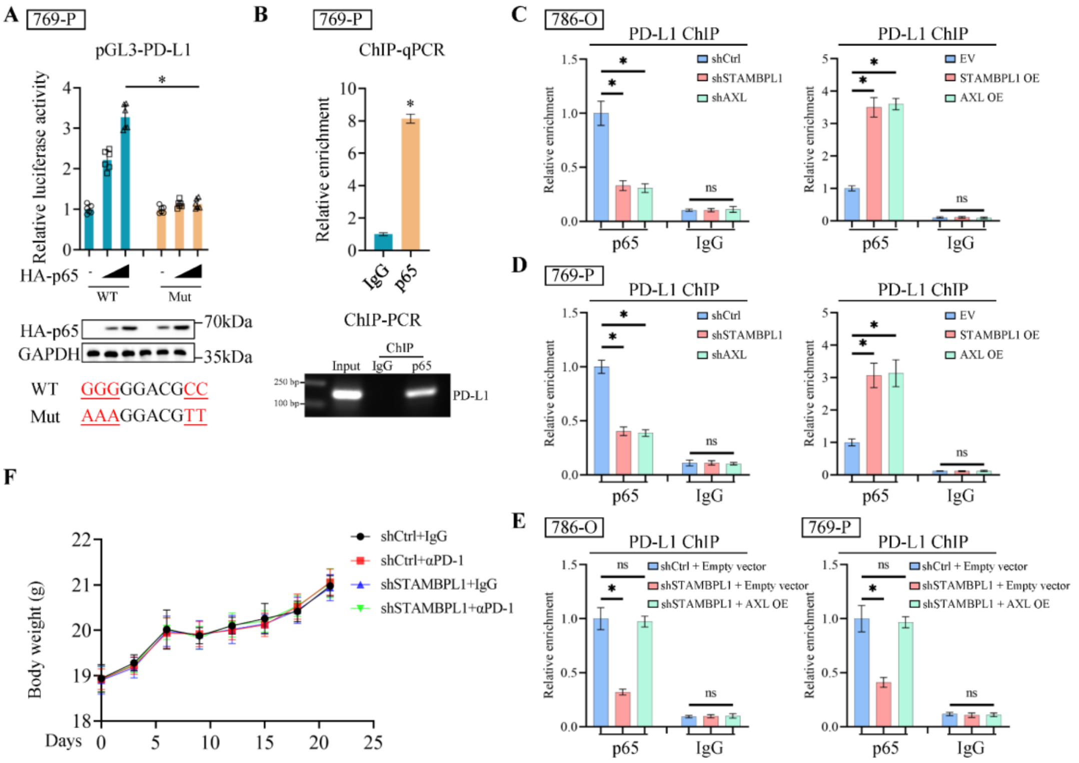


**Figure S25, related to Figure 6.** STAMBPL1 increases PD-L1 levels via stimulating p65 nuclear translocation and silencing STAMBPL1 potentiates the antitumor efficacy of PD-1 blockade. (A) Effects of p65 overexpression on relative luciferase activities of pGL3-PD-L1-WT/Mut vectors in 769-P cells (n=6) (top panel). The protein level of p65 was analyzed by immunoblotting (bottom panel). (B) ChIP-qPCR and ChIP-Semi-quantitative PCR analysis of p65 occupancy on PD-L1 promoter in 769-P cells (n=3). (C-E) ChIP-qPCR analysis of p65 occupancy on PD-L1 promoter in KIRC cells infected with the indicated lentiviruses (n=3). (F) During the experimental period, the body weight of tumor-bearing BALB/c mice was recorded every 3 days (n=5). All data are represented as mean ± SD, and analyzed using one-way ANOVA followed by Tukey post hoc test. For the analysis in (B), an unpaired two-tailed Student's t test was performed. *p<0.05; ns, not significant; Ctrl, control; WT, wild type; Mut, mutant; EV, empty, vector, OE, overexpressing.

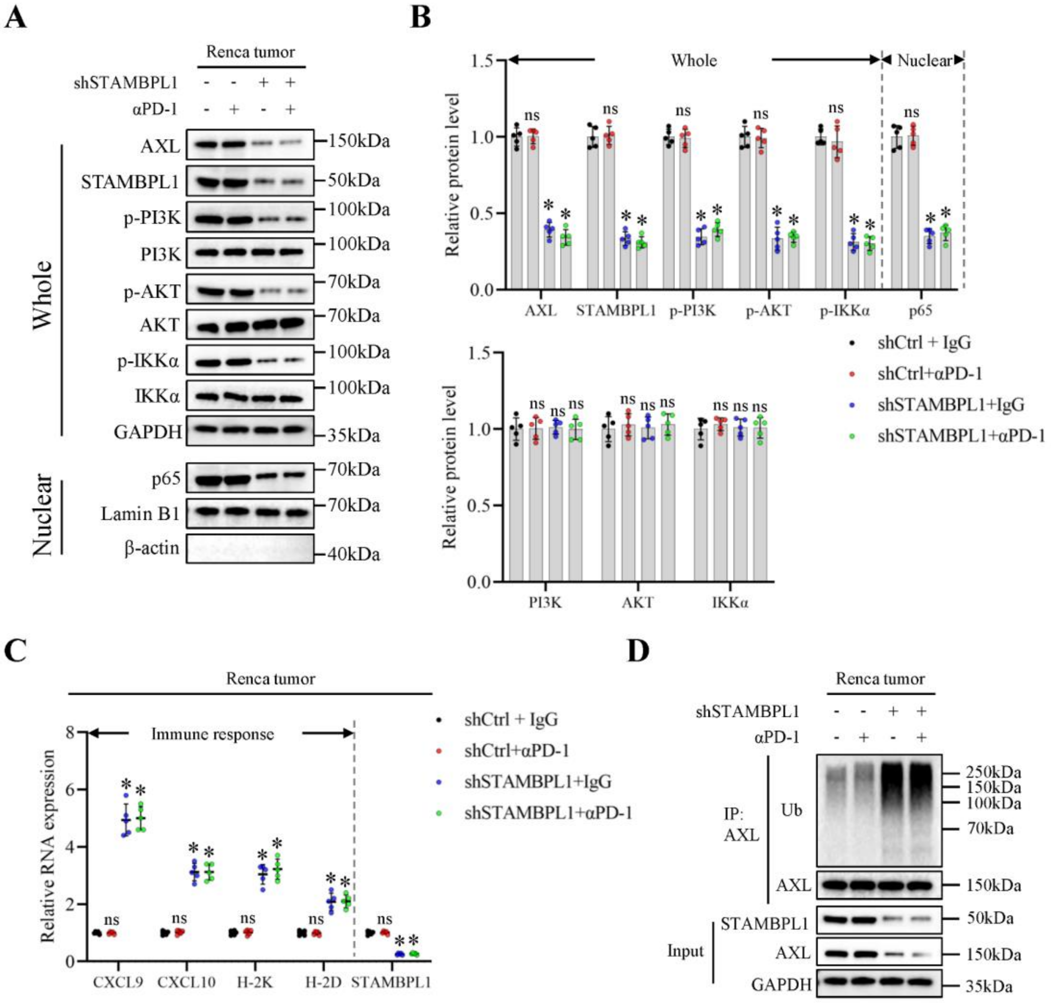


**Figure S26, related to Figure 6.** STAMBPL1 increases PD-L1 levels via stimulating p65 nuclear translocation and silencing STAMBPL1 potentiates the antitumor efficacy of PD-1 blockade. (A, B) Immunoblotting analysis of indicated proteins in excised subcutaneous Renca tumors (n=5). (C) RT-qPCR analysis of immune response genes in excised subcutaneous Renca tumors (n=5). (D) Immunoblotting analysis of WCL and anti-AXL IPs derived from lysates of excised subcutaneous Renca tumors using indicated antibodies (n=5). All data are represented as mean ± SD, and analyzed using one-way ANOVA followed by Tukey post hoc test. *$p<0.05$; ns, not significant; Ctrl, control.

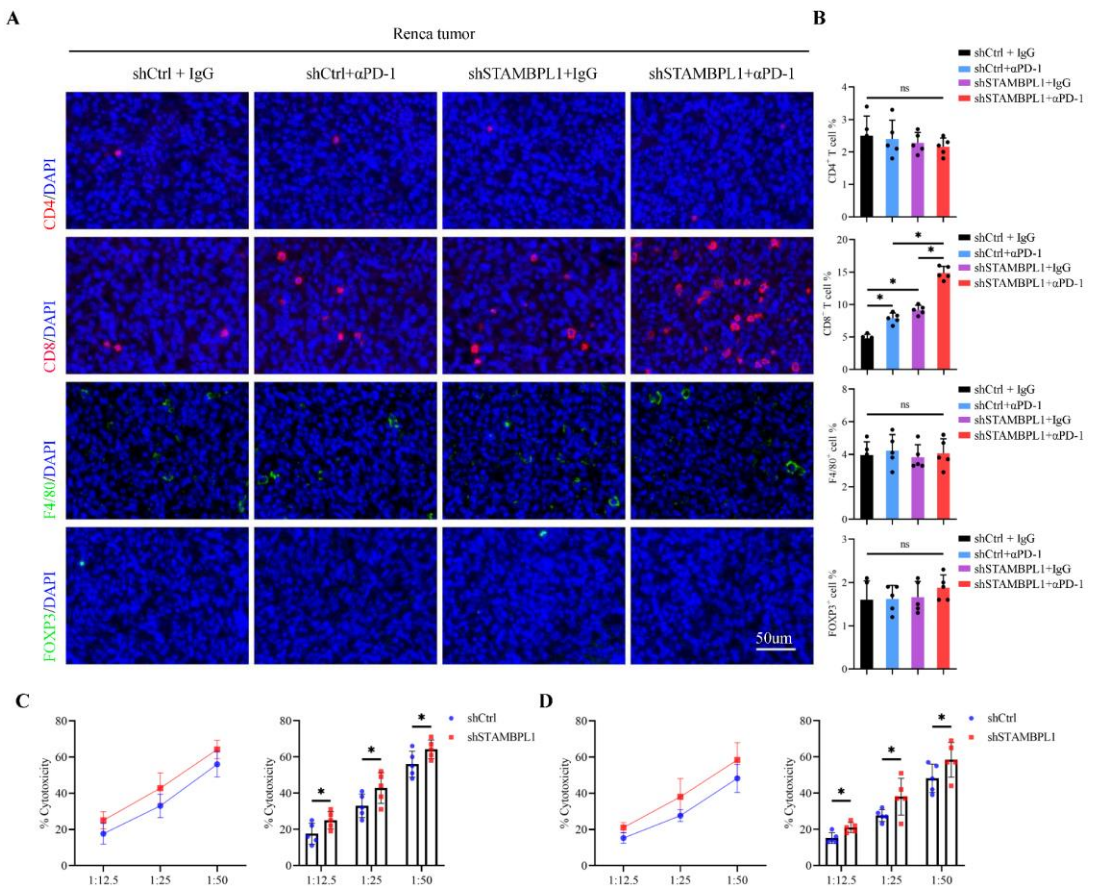


**Figure S27, related to Figure 6.** STAMBPL1 increases PD-L1 levels via stimulating p65 nuclear translocation and silencing STAMBPL1 potentiates the antitumor efficacy of PD-1 blockade. (A, B) Representative IF images (left panel) and quantification (right panel) of CD4+T cells, CD8+T cells, macrophages and Tregs in excised subcutaneous Renca tumors (n=5). CD8+T cells from healthy donors were activated for 48 hours with anti-CD3/CD28, and then cocultured with calcein-labeled KIRC cells at indicated target to effector ratios. After 4 hours, calcein release in supernatant was measured and percent cytotoxicity calculated. (C) CD8+T cells were cocultured with calcein-stained control or STAMBPL1 KD 786-O cells (n=5). (D) CD8+T cells were cocultured with calcein-stained control or STAMBPL1 KD 769-P cells (n=5). Same data visualized as an x,y plot (left panel) and dot plot (right panel). All data are represented as mean ± SD, and analyzed using an unpaired two-tailed Student′s t test. For the analysis in (B), one-way ANOVA followed by Tukey post hoc

test was performed. *p<0.05; ns, not significant; Ctrl, control.

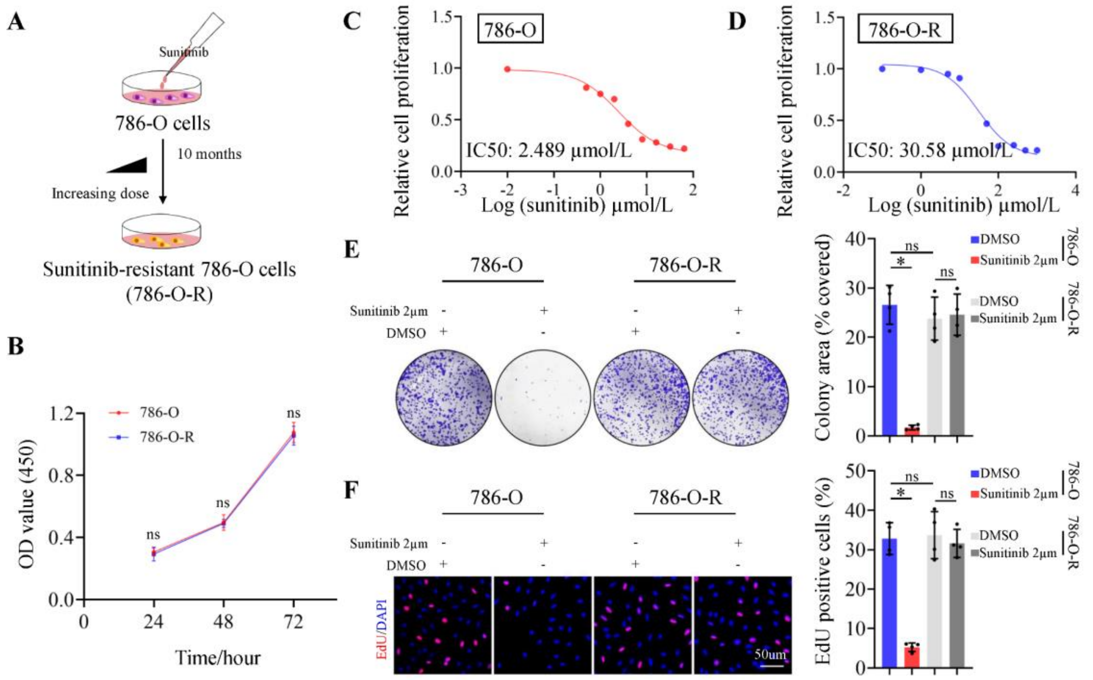


**Figure S28, related to Figure 7.** STAMBPL1 promotes sunitinib resistance in an AXL-dependent manner in KIRC. (A) The graphical representation of the sunitinib resistance model establishment. (B) CCK-8 assay for cell viability (n=3). (C, D) 786-O and 786-O-R cells were treated with a serial of concentration of sunitinib for 24 hours, and the IC50 values of sunitinib were measured using the CCK-8 assay. (E, F) Representative images and the quantitative results of colony formation and EdU assays (n=4). All data are represented as mean ± SD, and analyzed using one-way ANOVA followed by Tukey post hoc test. For the analysis in (B), an unpaired two-tailed Student′s t test was performed. *p<0.05; ns, not significant.

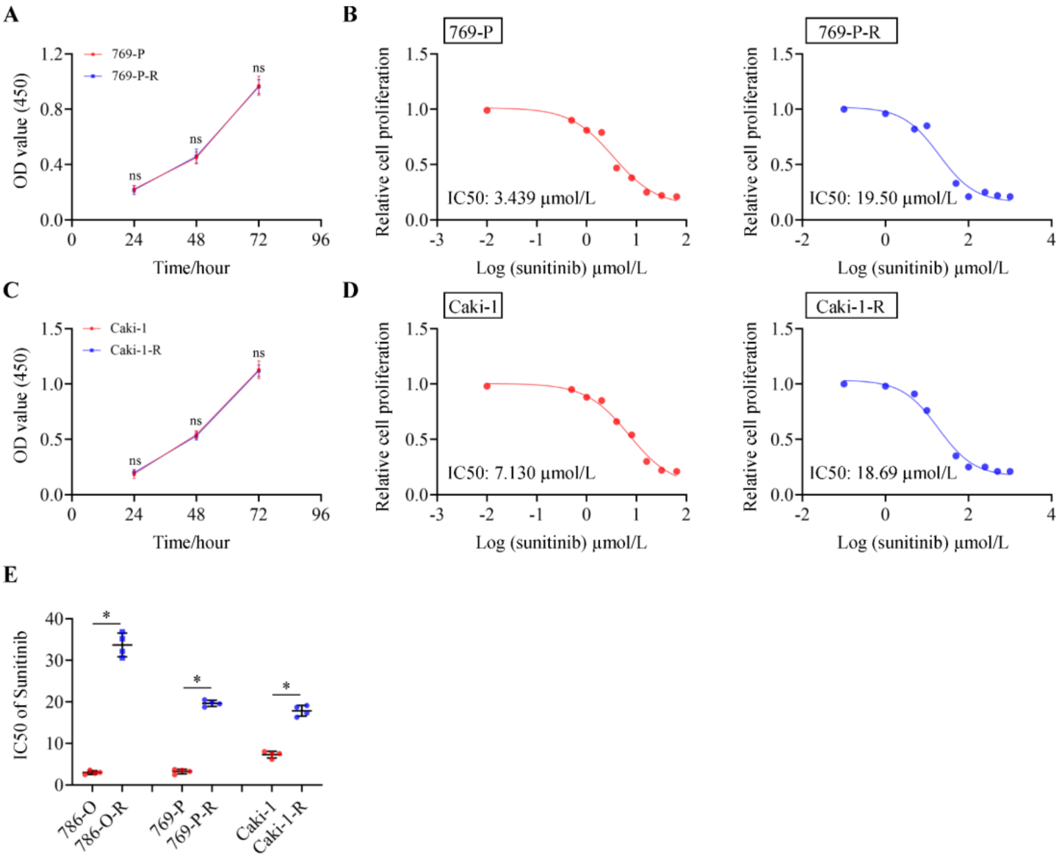


**Figure S29, related to Figure 7.** STAMBPL1 promotes sunitinib resistance in an AXL-dependent manner in KIRC. (A, C) CCK-8 assay for cell viability (n=3). (B, D)769-P, 769-P-R, Caki-1 and Caki-1-R cells were treated with a serial of concentration of sunitinib for 24 hours, and the IC50 values of sunitinib were measured using the CCK-8 assay. (E) The comparison of IC50 values of sunitinib between sunitinib-resistant KIRC cell lines and the parental cells (n=4). All data are represented as mean ± SD, and analyzed using an unpaired two-tailed Student's t test. *p<0.05; ns, not significant.

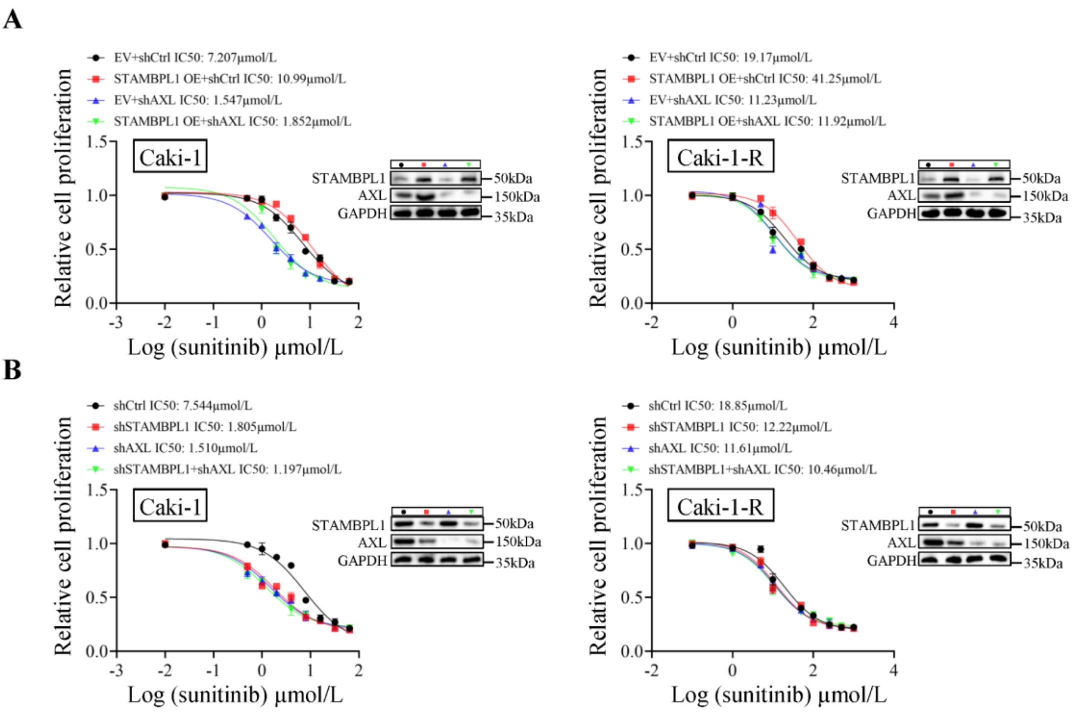


**Figure S30, related to Figure 7.** STAMBPL1 promotes sunitinib resistance in an AXL-dependent manner in KIRC. (A, B) Caki-1 and Caki-1-R cells infected with the indicated lentiviruses were treated with a serial dose of sunitinib for 24 hours and the IC50 values of sunitinib in each group were measured using the CCK-8 assay (n=3). Ctrl, control; EV, empty vector.

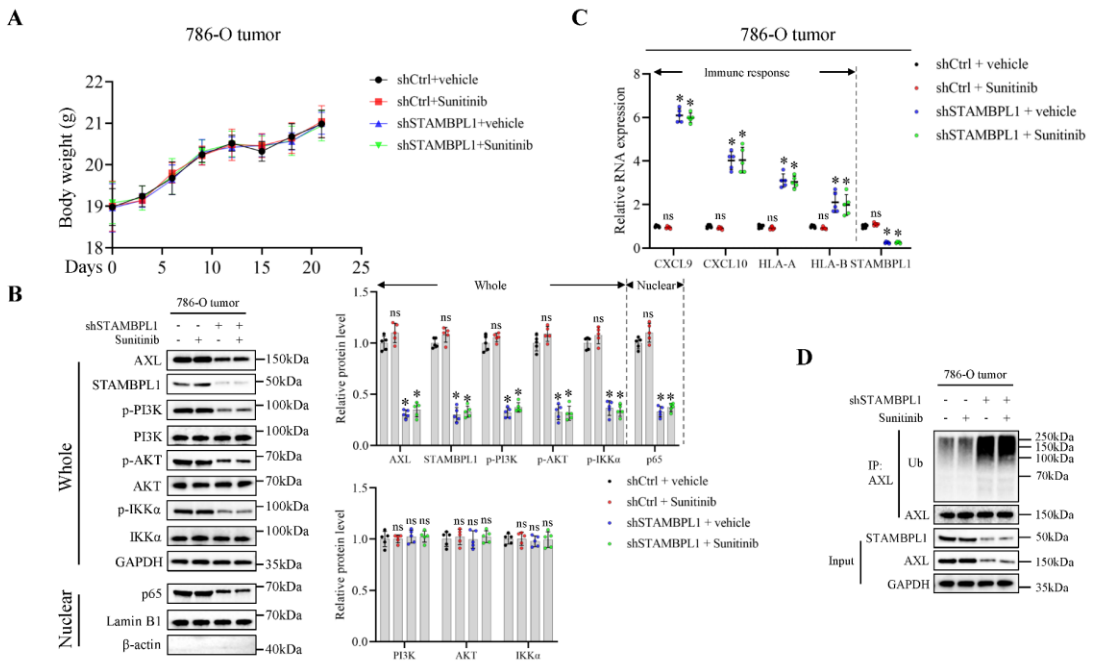


**Figure S31, related to Figure 7.** STAMBPL1 promotes sunitinib resistance in an AXL-dependent manner in KIRC. (A) During the experimental period, the body weight of tumor-bearing BALB/c nude mice was recorded every 3 days (n=5). (B) Immunoblotting analysis was performed to determine the levels of indicated proteins from tumor tissues of each group (n=5). (C) RT-qPCR analysis of immune response genes in tumor tissues (n=5). (D) Immunoblotting analysis of WCL and anti-AXL IPs derived from lysates of tumor tissues using indicated antibodies (n=5). All data are represented as mean ± SD, and analyzed using one-way ANOVA followed by Tukey post hoc test. *p<0.05; ns, not significant; Ctrl, control.

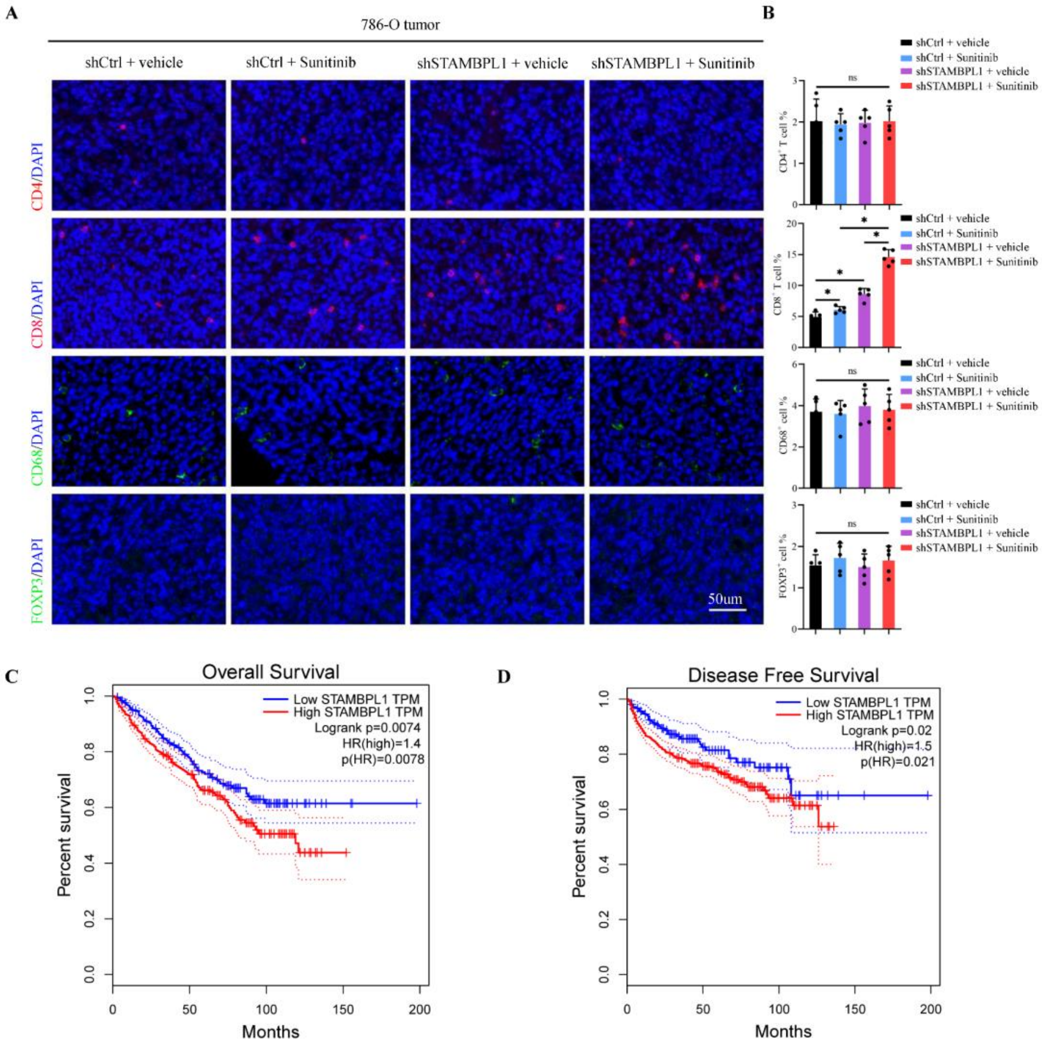


**Figure S32, related to Figure 7.** STAMBPL1 promotes sunitinib resistance in an AXL-dependent manner in KIRC. (A, B) Representative IF images (left panel) and quantification (right panel) of CD4+T cells (marked by CD4), CD8+T cells (marked by CD8), macrophages (marked by CD68) and Tregs (marked by FOXP3) in excised subcutaneous 786-O tumors (n=5). The association between STAMBPL1 and the prognosis of patients with renal cancer in TCGA was analyzed according to GEPIA database. (C) Kaplan–Meier plot of overall survival by STAMBPL1 expression. (D) Kaplan–Meier plot of disease-free survival by STAMBPL1 expression. All data are represented as mean ± SD, and analyzed using one-way ANOVA followed by Tukey post hoc test. For the analysis in (C, D), log-rank test was conducted. *p<0.05; ns, not significant; Ctrl, control.

# STAMBPL1/TRIM21 Balances AXL Stability Impacting Mesenchymal Phenotype and Immune Response in KIRC

Shiyu Huang [1, 2†], Xuke Qin [1, 2†], Shujie Fu [1, 2†], Juncheng Hu [1, 2†], Zhengyu Jiang [1, 2], Min Hu [3], Banghua Zhang[1, 2, 4], Jiachen Liu[1, 2, 5], Yujie Chen[1, 2], Minghui Wang[1, 2], Xiuheng Liu [1, 2*], Zhiyuan Chen [1, 2*], Lei Wang [1, 2*]

**Table of contents:**

Table S1……………………......................…………………………………………………36

Table S2……………………………………………….....................…………………………39

Table S3………………………………………………………….....................……………40

**Tables S1-3**

**Table S1. List of the primary antibodies used for immunoblotting (IB), immunoprecipitation (IP), immunofluorescence (IF) staining, immunohistochemical (IHC) staining, flow cytometry (FC) and ChIP assay in this study.**

| Antibodies | Source | Catalog No. | Application (dilution) |
|---|---|---|---|
| anti-STAMBPL1 | Proteintech | 27315-1-AP | IHC (1:200) |
| anti-STAMBPL1 | Abcam | ab229144 | IF (1:200) |
| anti-STAMBPL1 | Santa Cruz | sc-376526 | IB (1:200), IP (1:50) |
| anti-F-actin | Abcam | ab205 | IF (1:200) |
| anti-AXL | Proteintech | 13196-1-AP | IB (1:1000) |
| anti-AXL | CST | 8661 | IHC (1:500), IP (1:50) |
| anti-AXL | Santa Cruz | sc-166269 | IF (1:100) |
| anti-AXL | R&D Systems | FAB154P | FC (1:200) |
| anti-TYRO3 | Proteintech | 28513-1-AP | IB (1:500) |
| anti-TYRO3 | R&D Systems | FAB859P | FC (1:200) |
| anti-MERTK | Proteintech | 27900-1-AP | IB (1:500) |
| anti-MERTK | ABclonal | A26568 | FC (1:200) |
| anti-TRIM21 | Proteintech | 12108-1-AP | IB (1:5000), IHC (1:200) |
| anti-TRIM21 | CST | 92043 | IP (1:50) |
| anti-TRIM28 | Proteintech | 15202-1-AP | IB (1:1000) |
| anti-E-cadherin | Proteintech | 20874-1-AP | IB (1:20000), IF (1:200) |
| anti-Vimentin | Abcam | ab8978 | IB (1:1000), IF (1:200) |

| | | | |
|---|---|---|---|
| anti-HLA-A,B,C | Biolegend | 311406 | FC (1:200) |
| anti-Ubiquitin | Abcam | ab134953 | IB (1:1000) |
| anti-PD-L1 | CST | 13684 | IB (1:1000), IP (1:50) |
| anti-PD-L1 | Abcam | ab205921 | IF (1:200) |
| anti-PD-L1 | ABclonal | A22304 | FC (1:200) |
| anti-p65 | CST | 8242 | IB (1:1000), ChIP (1:100) |
| anti-Flag | CST | 14793 | IB (1:1000), IP (1:50) |
| anti-HA | CST | 3724 | IB (1:1000), IP (1:50) |
| anti-Lamin B1 | Proteintech | 12987-1-AP | IB (1:5000) |
| anti-β-actin | Abcam | ab8226 | IB (1:1000) |
| anti-GAPDH | Abcam | ab8245 | IB (1:1000) |
| anti-HRS | Proteintech | 10390-1-AP | IB (1:3000) |
| anti-STAM1 | Proteintech | 12434-1-AP | IB (1:2000) |
| anti-STAM2 | Proteintech | 13009-1-AP | IB (1:1000) |
| anti-AKT | Proteintech | 10176-2-AP | IB (1:2000), IP (1:50) |
| anti-p-AKT | Proteintech | 80455-1-RR | IB (1:3000) |
| anti-PI3K | CST | 4292 | IB (1:1000) |
| anti-PI3K | Abcam | ab28356 | IB (1:1000) |
| anti-PI3K | Proteintech | 25868-1-AP | IB (1:1000) |
| anti-p-PI3K | Invitrogen | PA5-118549 | IB (1:500) |
| anti-p-PI3K | CST | 4228 | IB (1:1000) |
| anti-IKKα | CST | 2682 | IB (1:1000) |

| | | | |
|---|---|---|---|
| anti-p-IKKα | CST | 2697 | IB (1:1000) |
| anti-p-IKKα | Abcam | ab138426 | IB (1:1000) |
| anti-p-IKKα | Abcam | ab38515 | IB (1:1000) |
| anti-IKKβ | CST | 8943 | IB (1:1000) |
| anti-NEMO | CST | 2685 | IB (1:1000) |
| anti-TRAF2 | Proteintech | 26846-1-AP | IB (1:500) |
| anti-TRAF5 | Proteintech | 12868-1-AP | IB (1:1000) |
| anti-TAK1 | Proteintech | 12330-2-AP | IB (1:800) |
| anti-TAB1 | Proteintech | 27566-1-AP | IB (1:500) |
| anti-TNFAIP3 | Proteintech | 23456-1-AP | IB (1:500) |
| anti-SOCS1 | CST | 55313 | IB (1:1000) |
| anti-SOCS3 | Proteintech | 14025-1-AP | IB (1:2000) |
| anti-p-AXL | CST | 5724 | IB (1:1000) |
| anti-p-AXL | CST | 96453 | IB (1:1000) |
| anti-$K^{63}$-Ub | CST | 5621 | IB (1:1000) |
| anti-CD4 | Servicebio | GB15064 | IF (1:200) |
| anti-CD8 | Servicebio | GB15068 | IF (1:200) |
| anti-F4/80 | Servicebio | GB113373 | IF (1:500) |
| anti-FOXP3 | Servicebio | GB112325 | IF (1:200) |
| anti-CD68 | Servicebio | GB113150 | IF (1:200) |
| anti-CD4 | CST | 93518 | IF (1:100) |
| anti-CD8 | Servicebio | GB12068 | IF (1:800) |

**Table S2. The sequences of shRNA used in this research.**

| GENE | Sequences (5′-3′) |
|---|---|
| shSTAMBPL1#1 (human) | GCTTCCTAACCATCGAGATTA |
| shSTAMBPL1#2 (human) | GCTGCTACTCTAAGTGCTGTT |
| shSTAMBPL1#3 (human) | GCTTGAGGTTTCTGCTTGTAA |
| shSTAMBPL1#4 (human) | CCAGAACAATTCCTTGCTGAA |
| shSTAMBPL1#1 (mouse) | CGGAGTGGAAATGGAAAGGAT |
| shSTAMBPL1#2 (mouse) | TTTCAGAAGTGACTGATATTT |
| shTRIM21#1 (human) | TGAGAAGTTGGAAGTGGAAAT |
| shTRIM21#2 (human) | TGGCATGGTCTCCTTCTACAA |
| shAXL#1 (human) | CTTTAGGTTCTTTGCTGCATT |
| shAXL#2 (human) | GCGGTCTGCATGAAGGAATTT |
| shTRIM28#1 (human) | CCTGGCTCTGTTCTCTGTCCT |
| shTRIM28#2 (human) | CTGAGACCAAACCTGTGCTTA |
| shRELA/p65#1 (human) | GCCTTAATAGTAGGGTAAGTT |
| shRELA/p65#2 (human) | CGGATTGAGGAGAAACGTAAA |
| shTNFAIP3#1 (human) | CGGCTATGACAGCCATCATTT |
| shTNFAIP3#2 (human) | GCACCGATACACACTGGAAAT |
| shHRS#1 (human) | CCGCATGAAGAGTAACCACAT |
| shHRS#1 (human) | CCTGTACTCTTCACCTGTGAA |
| shSTAM1#1 (human) | CCCTTTCCACTTTGTATCCAA |

| | |
|---|---|
| shSTAM1#1 (human) | TGTGTATCAAACTGTGGCAAA |
| shSTAM2#1 (human) | GCACGGAAAGTGAGAGCTTTA |
| shSTAM2#1 (human) | GCACCAGTGTACTCAGTCTAT |

**Table S3 Indicated primers used in RT-qPCR experiments.**

| **GENE** | **Primer sequences (5′-3′)** |
|---|---|
| STAMBPL1 *(human)* | F: GAGGATGGCGTCTGTGTATTTG |
| | R: GCTGGTAATCTCGATGGTTAGG |
| GAPDH *(human)* | F: TCCATGACAACTTTGGTATCGTG |
| | R: ACAGTCTTCTGGGTGGCAGTG |
| Vimentin *(human)* | F: TGTCCAAATCGATGTGGATGTTTC |
| | R: TTGTACCATTCTTCTGCCTCCTG |
| N-cadherin *(human)* | F: TCAGGCGTCTGTAGAGGCTT |
| | R: ATGCACATCCTTCGATAAGACTG |
| TWIST *(human)* | F: CCAGCTTGAGGGTCTGAATC |
| | R: GTCCGCAGTCTTACGAGGAG |
| Fibronectin *(human)* | F: AAACCTCGGCTTCCTCCATAA |
| | R: CGGTGGCTGTCAGTCAAAG |
| CXCL9 *(human)* | F: CCAGTAGTGAGAAAGGGTCGC |
| | R: AGGGCTTGGGGCAAATTGTT |
| CXCL10 *(human)* | F: GCCTTCGATTCTGGATTCAG |
| | R: GTGGCATTCAAGGAGTACCTC |

| | |
|---|---|
| HLA-A *(human)* | F: CGACGCCGCGAGCCAGA |
| | R: GCGATGTAATCCTTGCCGTCGTAG |
| HLA-B *(human)* | F: GACGGCAAGGATTACATCGCCCTGAA |
| | R: CACGGGCCGCCTCCCACT |
| AXL *(human)* | F: CGAAAGAAGGAGACCCGTTATG |
| | R: ATAGAGGAGGAAGCTGTGTAGG |
| TYRO3 *(human)* | F: CAGCCGGTGAAGCTCAACT |
| | R: TGGCACACCTTCTACCGTGA |
| MERTK *(human)* | F: ACCTCTGTCGAATCAAAGCCC |
| | R: CTGCACACTGGTTATGCTGAA |
| PD-L1 *(human)* | F: TGGCATTTGCTGAACGCATTT |
| | R: TGCAGCCAGGTCTAATTGTTTT |
| TRIM21 *(human)* | F: CTTGCTTCTGAGCGGAAACT |
| | R: AGGCAGATAGGGCATGTGAC |
| TNFAIP3 *(human)* | F: GATAGAAATCCCCGTCCAAGG |
| | R: CTGCCATTTCTTGTACTCATGC |
| E-cadherin *(human)* | F: TCCCAGGCGTAGACCAAGA |
| | R: ATTTTTCCCTCGACACCCGAT |
| U1 *(human)* | F: GGGAGATAACGTGACCACGAAG |
| | R: CCACAAATTATGCAGTCGAGTTTC |
| SOCS1 *(human)* | F: CACGCACTTCCGCACATTC |
| | R: TAAGGGCGAAAAAGCAGTTCC |

| | |
|---|---|
| SOCS3 *(human)* | F: CCTGCGCCTCAAGACCTTC |
| | R: GTCACTGCGCTCCAGTAGAA |
| H-2K *(mouse)* | F: GGCAATGAGCAGAGTTTCCGAG |
| | R: CCACTTCACAGCCAGAGATCAC |
| H-2D *(mouse)* | F: TGAGGAACCTGCTCGGCTACTA |
| | R: GGTCTTCGTTCAGGGCGATGTA |
| GAPDH *(mouse)* | F: AGGTCGGTGTGAACGGATTTG |
| | R: TGTAGACCATGTAGTTGAGGTCA |
| STAMBPL1 *(mouse)* | F: CGCGCCCTAAGCAAACTTG |
| | R: ACACTGCTGGTAATCTCGGTG |
| AXL *(mouse)* | F: ATGGCCGACATTGCCAGTG |
| | R: CGGTAGTAATCCCCGTTGTAGA |
| CXCL9 *(mouse)* | F: GGAGTTCGAGGAACCCTAGTG |
| | R: GGGATTTGTAGTGGATCGTGC |
| CXCL10 *(mouse)* | F: CCAAGTGCTGCCGTCATTTTC |
| | R: GGCTCGCAGGGATGATTTCAA |
